# Is the kernel–staples match a key–lock match?†
†Electronic supplementary information (ESI) available: CCDC 1587211. For ESI and crystallographic data in CIF or other electronic format see DOI: 10.1039/c7sc05019d


**DOI:** 10.1039/c7sc05019d

**Published:** 2018-01-29

**Authors:** Shengli Zhuang, Lingwen Liao, Yan Zhao, Jinyun Yuan, Chuanhao Yao, Xu Liu, Jin Li, Haiteng Deng, Jinlong Yang, Zhikun Wu

**Affiliations:** a Key Laboratory of Materials Physics , Anhui Key Laboratory of Nanomaterials and Nanotechnology , CAS Center for Excellence in Nanoscience , Institute of Solid State Physics , Chinese Academy of Sciences , Hefei , Anhui 230031 , P. R. China . Email: zkwu@issp.ac.cn; b Department of Chemistry , University of Science and Technology of China , Hefei , Anhui 230026 , P. R. China; c Institute of Physical Science and Information Technology , Anhui University , Hefei , Anhui 230601 , P. R. China; d Hefei National Laboratory for Physics Sciences at the Microscale , University of Science and Technology of China , Hefei , Anhui 230026 , P. R. China; e Tsinghua University-Peking University Joint Center for Life Sciences , School of Life Sciences , Tsinghua University , Beijing 100084 , P. R. China; f MOE Key Laboratory of Bioinformatics , School of Life Sciences , Tsinghua University , Beijing 100084 , P. R. China

## Abstract

Metal nanoclusters provide excellent references for understanding metal nanoparticle surfaces, which remain mysterious due to the difficulty of atomically precise characterization. Although some remarkable advances have been achieved for understanding the structure of metal nanoclusters, it is still unknown if the inner kernel–outer staples match is a key–lock match and how the surface staples influence some of the properties of metal nanoclusters. Herein, we have developed an acid-induction method for synthesizing a novel gold nanocluster whose composition is determined to be Au_42_(TBBT)_26_ (TBBT: 4-*tert*-butylbenzenelthiolate) by ESI-MS and single-crystal X-ray crystallography (SCXC). SCXC also reveals that Au_42_(TBBT)_26_ has an identical kernel but different staples with an existing gold nanocluster Au_44_(TBBT)_28_, indicating that the kernel–staples match is not a key–lock match and the existence of homo-ligand–homo-kernel–hetero-staples phenomenon in metal nanoclusters provides some reference for understanding the growth or transformation of metal nanoclusters. Further experiments reveal that the staples greatly contribute to the stability of gold nanoclusters and influence their photoluminescence intensity and that minute differences in the interfacial structure can lead to enhanced stability and photoluminescence.

## Abstract 2

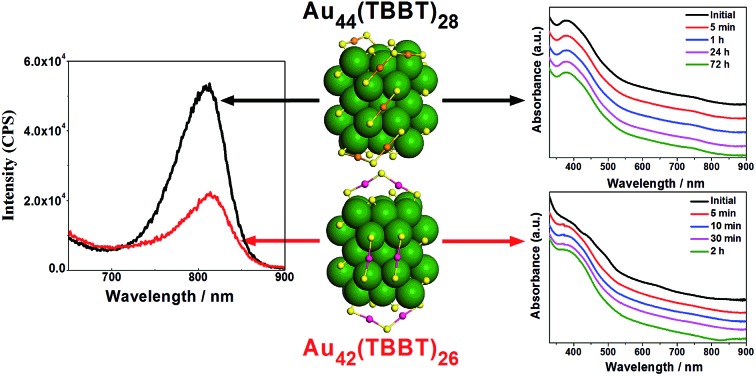
We demonstrated the existence of homo-ligand–homo-kernel–hetero-staples phenomenon in metal nanoclusters and the remarkable change in their properties by subtle interfacial structure tailoring.

## Introduction

Ligand-protected metal nanoparticles have received extensive interest not only for fundamental scientific research but also for their practical applications over the past several decades.^1^ However, the surfaces of the nanoparticles remain mysterious due to the difficulty of atomically precise characterization. The recently emergent metal nanoclusters^2^ provide excellent references for understanding metal nanoparticle surfaces because the compositions of the metal nanoclusters can be precisely determined by mass spectrometry and because the total structures of metal nanoclusters can be resolved by single-crystal X-ray crystallography (SCXC). To date, over a dozen metal nanoclusters2p,^3^ have been structurally resolved by SCXC, which reveals that metal nanoclusters consist of pure metal kernels and ligand-containing staples (herein, the bridging thiolates are also included in the staples for simplification). It is believed that the kernel is protected from aggregation or decomposition by the staples;^4^ thus, the staples should match the kernel, otherwise the nanocluster cannot be stable.

Moreover, some theoretical calculations indicate that the protecting staple motifs should fit the curvature of the kernel of the nanoclusters.^5^ Essential questions include the following: is this match a key–lock match (*i.e.*, if the metal, the thiolate and the kernel are all fixed, are the staples exclusive)? If not, will a variation of the staples lead to great change in the stability and other properties (*e.g.*, photoluminescence) of nanoclusters? These questions are important to understand the interaction between staples and the kernel, the staples' influence on the properties, and the growth (or transformation) mechanism of metal nanoclusters.^6^ To address these questions, we have developed a method to synthesize a novel gold nanocluster protected by 4-*tert*-butylbenzenelthiolate (TBBT), which has identical Au_34_ kernels but varied staples with an existing gold nanocluster-Au_44_(TBBT)_28_, and we compare the stability and photoluminescence properties between the two homo-kernelled gold nanoclusters.

## Results and discussion

The synthesis of Au_42_(TBBT)_26_ nanoclusters was according to the method described by us in our previous study.3*v* Some modifications were made to this method, and the details are provided in the experimental section. Briefly, tetraoctylammonium bromide (TOAB) was dissolved in ethyl acetate in a tri-neck flask, and HAuCl_4_·4H_2_O was added to the solution. After the solution was stirred for 10 min, 4-*tert*-butylbenzenelthiol was added. When the color of the solution changed from deep red to yellow, an acetic acid-water mixture was added, and then, a cold water solution of NaBH_4_ was added all at once immediately. Please note that without the addition of acid, the Au_42_(TBBT)_26_ nanoclusters cannot be obtained, and very recently, we synthesized a rare aliphatic ligand-stabilized gold nanocluster with an fcc structure by similar protocols except that acetic acid was replaced by nitric acid in the reaction, indicating that these synthesis protocols are not exclusive and can be developed to a method (tentatively dubbed acid-induction synthesis method) for synthesizing novel nanoclusters that are otherwise difficult to obtain. Herein, acetic acid could also be replaced by other acids (nitric acid, hydrochloric acid, *etc.*), however, it showed better reproducibility compared with the other investigated acids in this work. Acetic acid played at least two roles: on one hand, acetic acid increased the hydrolysis of NaBH_4_ and strengthened the reduction of NaBH_4_;^7^ on the other hand, it could weaken the interaction between Au and thiolate, reducing the reactivity of thiolate. Thus, the attack of acid probably influenced the kinetics and thermodynamics of the formation of nanocluster products^8^ similar to that in a previous work reported by Xie *et al.*^9^ who added a base instead to alter the reaction kinetics and thermodynamics. The growth of gold nanoclusters in the reaction was allowed to proceed for 2 h for enhancement in size.^10^ The crude product was collected and thoroughly washed with methanol, and Au_42_(TBBT)_26_ nanoclusters were separated by preparative thin-layer chromatography (PTLC).3j,^11^

The precise composition of the Au_42_(TBBT)_26_ nanocluster was determined by electrospray ionization mass spectrometry (ESI-MS).3g,3k,^8^,^12^ To impart charges, cesium acetate (CsOAC) was added to the nanocluster solution to form cationic cluster adducts with Cs^+^. The ESI-MS spectrum showed intense peaks centered at *m*/*z* 6418.44 (see Fig. S1†), corresponding to the [Au_42_(TBBT)_26_Cs_2_]^2+^ species (calculated: 6418.17 Da, deviation: 0.27 Da). Because the charge number was equal to the number of adducted Cs^+^ ions, the Au_42_(TBBT)_26_ nanoclusters were proposed to be charge-neutral, which is supported by the fact that no sound signals were found in the mass spectrum without the addition of CsOAC and that the counter ions were not found by SCXC. Dark rhombic single crystals were formed in a mixed solution of toluene and acetonitrile after 3 days. The total structure of Au_42_(TBBT)_26_ was resolved by SCXC. The Au_42_(TBBT)_26_ crystal structure adopts the triclinic space group P-1. Similar to its counterpart, Au_44_(TBBT)_28_,3*p* Au_42_(TBBT)_26_ has a pair of enantiomeric nanoclusters in the unit cell. The left-handed isomer was chosen to explore the details of the atom packing structure of Au_42_(TBBT)_26_ and Au_44_(TBBT)_28_.

**Fig. 1:**
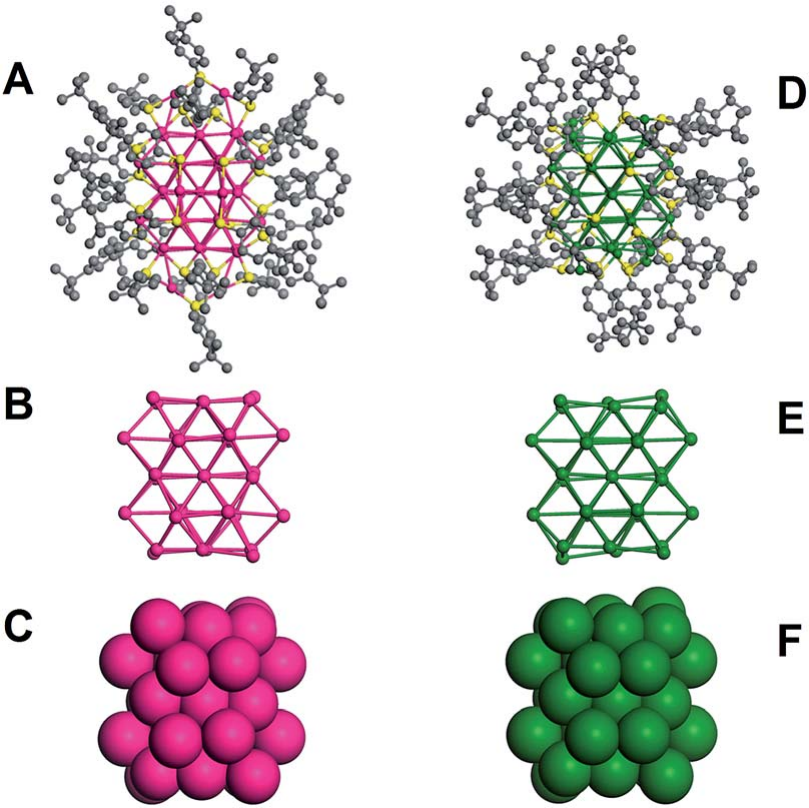
Total structure and Au_34_ kernels of Au_42_(TBBT)_26_ (A–C) and Au_44_(TBBT)_28_ (D–F).

**Fig. 2:**
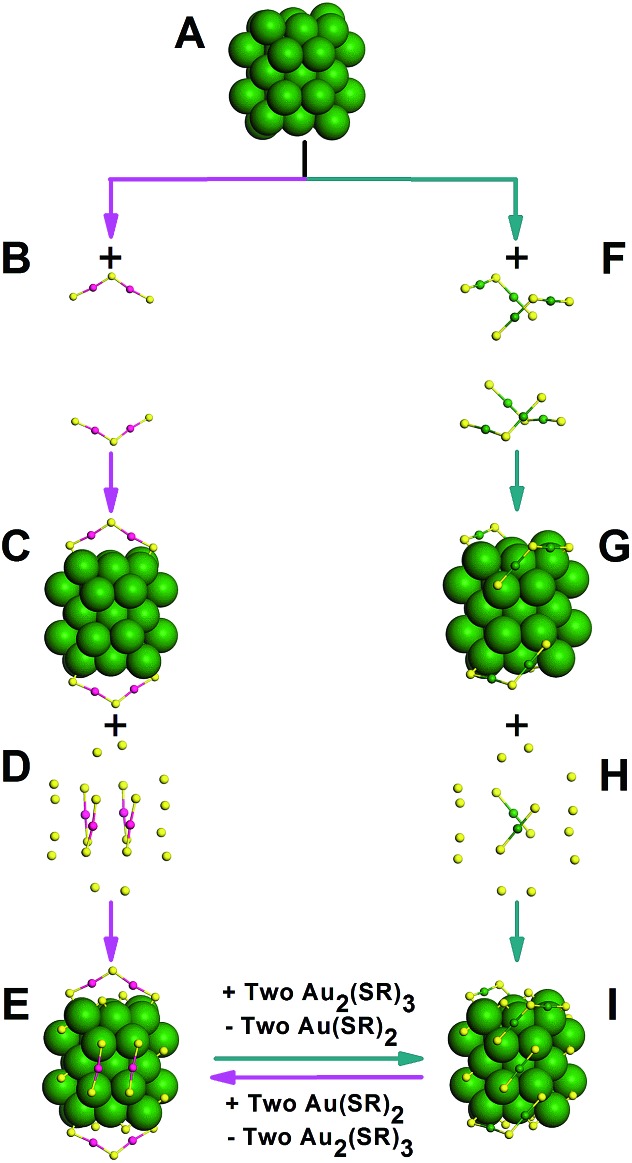
Surface staples comparison of Au_42_(TBBT)_26_ and Au_44_(TBBT)_28_: Au_34_ kernel (A); illustrating the anatomy of two Au_2_(TBBT)_3_, four Au(TBBT)_2_ and 12 TBBT capped on the kernel of Au_42_(TBBT)_26_ (from B to E); illustrating the anatomy of four Au_2_(TBBT)_3_, two Au(TBBT)_2_ and 12 TBBT capped on the kernel of Au_44_(TBBT)_28_ (from F to I). For clarity, C and H are omitted. Color labels: yellow = S; others = Au.

The total structure of Au_42_(TBBT)_26_ is shown in Fig. 1A. The kernel of Au_42_(TBBT)_26_ is composed of four cuboctahedra, which are formed by 34 gold atoms, as shown in Fig. 1B, and it is identical to the kernel of Au_44_(TBBT)_28_ (ref. 3p) (see Fig. 1E; the total structure of Au_44_(TBBT)_28_ is shown in Fig. 1D). Please note that there are some other kernel assignments on the basis of different views.3*p* In another view, the two kernels adopt identical FCC packing as shown in Fig. 1C and F. However, although the two gold nanoclusters have identical Au_34_ kernels and protecting thiolate (TBBT), they have different staples. To better illustrate this, we give a detailed structural interpretation of the staples' species and adsorption sites (see Fig. 2).

**Fig. 3:**
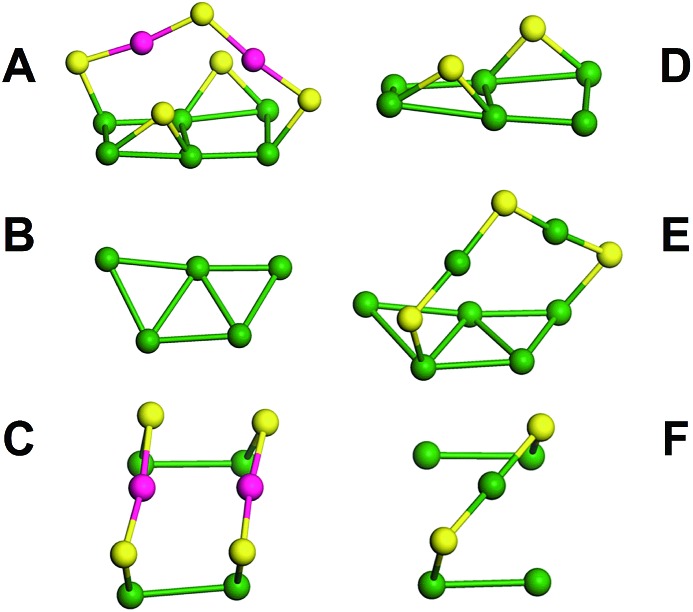
Staples and bridging thiolates on the crystalline facets of Au_42_(TBBT)_26_ and Au_44_(TBBT)_28_; bridging thiolates, a dimeric staple and monomeric staple on the {100}, {111} and {110} facets of Au_42_(TBBT)_26_, respectively (A, B and C); bridging thiolates, a dimeric staple and monomeric staple on the {100}, {111} and {110} facets of Au_44_(TBBT)_28_, respectively (D, E, and F). Color labels: yellow = S; others = Au.

The kernel of Au_44_(TBBT)_28_ is protected by four Au_2_(TBBT)_3_ dimers at the top and bottom and two Au(TBBT)_2_ monomers at the waist (Fig. 2F–I).3*p* In contrast, there are two Au_2_(TBBT)_3_ dimers at the top and bottom of the Au_42_(TBBT)_26_ nanocluster and four Au(TBBT)_2_ monomers at the waist (Fig. 2B–E). Of note, there are also 12 bridging thiolates for every nanocluster (see Fig. 2D and H). In addition to quantitative differences in the staples, the surface arrangement of the staples is varied as mentioned above. In Au_42_(TBBT)_26_, the bridging thiolates and dimeric staples anchor the {100} facets (Fig. 3A), and each of the monomer staples anchor each {110} facet (Fig. 3C), with no staples anchoring the {111} facet (Fig. 3B). However, in Au_44_(TBBT)_28_, the {100}, {111} and {110} facets are protected by bridging thiolates, a dimeric staple and monomeric staple, respectively (Fig. 3D–F). Please note that each {111} facet is protected by one dimeric staple and each {100} square is protected by one bridging thiolate in the magic cluster series including Au_28_(TBB)_20_, Au_36_(TBBT)_24_, Au_44_(TBBT)_28_ and Au_52_(TBBT)_32_.3*p*

The average Au–S bond length of the staples in Au_42_(TBBT)_26_ (2.31 Å) is slightly shorter than that in Au_44_(TBBT)_28_ (2.33 Å).3*p* The average Au–S–Au and S–Au–S bond angles of the staples in Au_42_(TBBT)_26_ (112.6° and 171.2°, respectively) are different from those in Au_44_(TBBT)_28_ (100.1° and 171.8°, respectively). Please note that the bridging thiolates are not considered in the above comparisons.

**Fig. 4:**
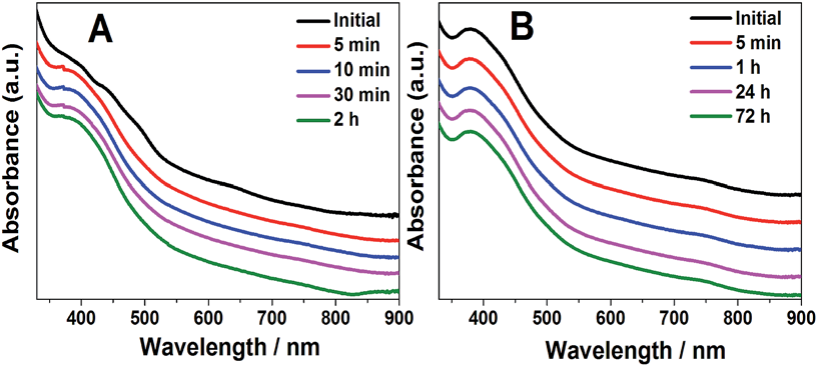
Thermostability of Au_42_(TBBT)_26_ (A) and Au_44_(TBBT)_28_ (B).

Based on the above discussion, Au_42_(TBBT)_26_ has an identical Au_34_ kernel but different staples, which indicates that the kernel–staples match is not a key–lock match and that the homo-ligand–homo-kernel–hetero-staples phenomenon exists in metal nanoclusters, which has not been previously reported to the best of our knowledge. Of note, it is known that the hetro-ligand–homo-kernel–hetero-stapled metal nanoclusters have been known for some time.3f,12b,^13^ The search for the homo-ligand–homo-kernel–hetero-staples phenomenon in metal nanoclusters indicates that the protecting staples on the kernel have some flexibility, which may provide some reference for understanding the growth or transformation of metal nanoclusters.^6^ The pair of homo-ligand–homo-kernel–hetero-stapled nanoclusters Au_42_(TBBT)_26_ and Au_44_(TBBT)_28_ is ideal materials to investigate the influence that the staples have on nanocluster properties without interferences from some other factors (*e.g.* ligands). As discussed above, it is believed that the staples protect the kernels from aggregation or decomposition. This is indeed indicated by our stability experiments, which reveal that Au_44_(TBB)_28_ in toluene is stable for at least 72 hours under 80 °C monitored by UV/vis/NIR spectrometry, whereas the less-stapled Au_42_(TBBT)_26_ is not stable for even 10 min under similar conditions (see Fig. 4). It is worth noting that there are some other perspectives to interpret the stability of metal nanoclusters.2a,^14^

**Fig. 5:**
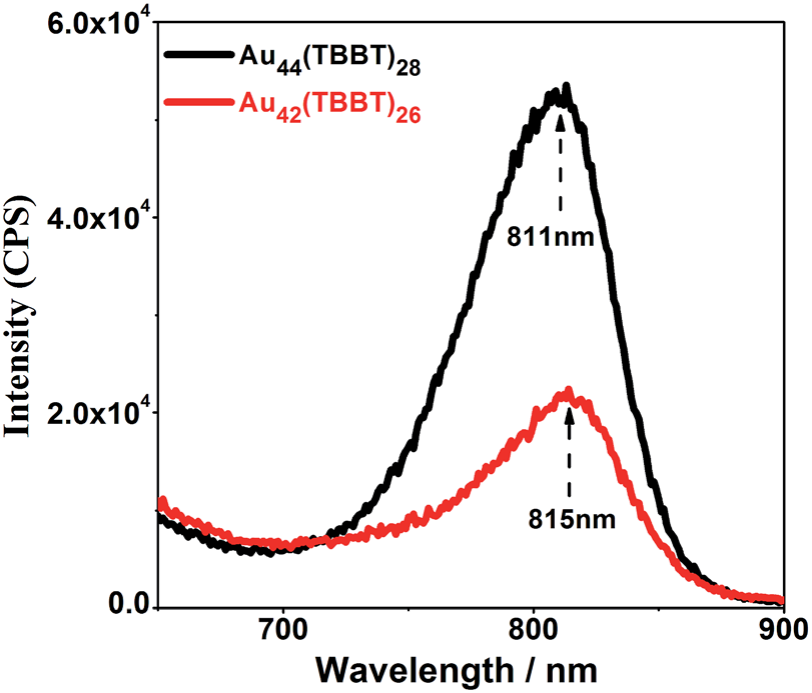
Photoluminescence spectra of Au_44_(TBBT)_28_ and Au_42_(TBBT)_26_.

Photoluminescence is an intriguing phenomenon, and the photoluminescence of gold/silver nanoclusters has recently received increasing attention not only for fundamental scientific research but also for practical applications in a large range of fields such as sensors2i,2l,^15^ and biomedicine.2d,15f,^16^ For fundamental scientific research, studies on the photoluminescence mechanism of gold/silver nanoclusters are of major importance, and there have been some pre-eminent contributions from different groups. For instance, Murray's group^17^ found that the luminescence intensity of Au_25_(SR)_18_ increases linearly with the increasing number of polar ligands on the nanoclusters. Additionally, Wu and Jin^18^ suggested that surface ligands can influence the photoluminescence by charge transfer and direct delocalized electron donation. Moreover, Xie *et al.*^19^ found that aggregation-induced emission (AIE) also exists in gold nanoclusters, and Lee's group^20^ revealed that the rigidity of the Au(i)-thiolate shell (staple) is beneficial to the emission of gold nanoclusters. Recently, our group16*g* discovered that both interlocked Au_4_(SR)_5_ staples and strengthened interactions between the kernel and the thiolates contribute to the enhanced photoluminescence of Au_24_(SR)_20_ nanoclusters. Even more recently, theoretical calculations conducted by Aikens and colleagues showed that the photoluminescence of gaseous Au_25_(SR)_18_^–^ originates from kernel-based electron transitions rather than charge-transfer or semi-ring states.^21^ Despite these distinguished contributions, the understanding of the photoluminescence mechanism of gold/silver nanoclusters is far from complete, and a major puzzle lies in the real roles of the kernel and staples in emission. This issue is challenging because the kernels and staples cannot be isolated and investigated separately. Thus, the finding of homo-ligand–homo-kernel–hetero-stapled gold nanoclusters might provide an opportunity to understand this issue. Although homo-kernelled, Au_42_(TBBT)_26_ and Au_44_(TBBT)_28_ (ref. 3p) have only slight difference in staples and show big difference in emission intensities: the more-stapled Au_44_(TBBT)_28_ exhibits ∼2 times stronger photoluminescence than the less-stapled Au_42_(TBBT)_26_ (see Fig. 5), indicating that the staples greatly contribute to the photoluminescence intensity of metal nanoclusters, which is also supported by the previous observations that ligands12c,^18^ and solvents3*t* have important influence on the photoluminescence intensities of metal nanoclusters. The two nanoclusters have different excitation spectra, however, they have a close maximum excitation wavelength at ∼470 nm (Fig. S2†). They have also similar emission profiles when excited at 514 nm although their emission intensities are pre-eminently different (Fig. 5). In addition, the photoluminescence lifetimes of Au_44_(TBBT)_28_ (15.59 ns (15.23%) and 71.99 ns (84.77%)) are close to those of Au_42_(TBBT)_26_ (14.96 ns (13.10%) and 89.00 ns (86.90%)) (see Fig. S3†). Taken together, these results indicate that the two nanoclusters have a similar photoluminescence mechanism. Please note that a high Au(i)/Au(0) ratio in the nanoclusters cannot be the cause of stronger photoluminescence observed in our research because Au_42_(TBBT)_26_ has a higher Au(i)/Au(0) ratio than Au_44_(TBBT)_28_ (see Fig. S4†) but less photoluminescence intensity than that of Au_44_(TBBT)_28_.^19^

## Conclusions

In summary, an acid-induction method was developed to synthesize a novel gold nanocluster Au_42_(TBBT)_26_ whose composition was determined by using ESI-MS and SCXC and whose structure was resolved by SCXC. The as-obtained gold nanocluster had an identical Au_34_ kernel but reduced staples compared with those of the existing Au_44_(TBBT)_28_ nanocluster, indicating that the kernel–staples match was not a key–lock match and the existence of homo-ligand–homo-kernel–hetero-staples phenomenon in metal nanoclusters. The two Au-SR units' reduction led to a decreased stability of Au_42_(TBBT)_26_ compared with that of Au_44_(TBBT)_28_, thus experimentally confirming the importance of the staples in protecting the kernels of metal nanoclusters from aggregation or decomposition. Furthermore, the reduced staples resulted in a photoluminescence decrease by ∼2 times, indicating that the staples greatly influenced the photoluminescence intensity of metal nanoclusters. The novelty and significance of this work lie in the following: (i) an acid-induction method was developed to synthesize a novel gold nanocluster Au_42_(TBBT)_26_; (ii) the structure of Au_42_(TBBT)_26_ was resolved by SCXC; (iii) the structure comparison between Au_44_(TBBT)_28_ and Au_42_(TBBT)_26_ indicated the existence of homo-ligand–homo-kernel–hetero-staples phenomenon in metal nanoclusters and that the kernel–staples match is not a key–lock match, which might have some important implication for understanding the growth or transformation of metal nanoclusters; (iv) it experimentally confirmed that the staples stabilize the gold nanoclusters; and (v) it was first unambiguously demonstrated that the staples greatly influence the photoluminescence intensity without interferences from other factors (*e.g.* ligands), which may have important implications on the understanding of the photoluminescence mechanism of metal nanoclusters. It is expected that our work will trigger more studies on the synthesis, structures and properties of metal nanoclusters and have important implications on understanding the kernel–staples interaction, staples-properties correlation and growth (or transformation) mechanism of metal nanoparticles.

## Conflicts of interest

There are no conflicts to declare.

## Supplementary Material

Supplementary information

Crystal structure data
