# Supplementary material for: Is the kernel–staples match a key–lock match?
Source: Chem Sci. 2018 Jan 29;9(9):2437–42. doi: 10.1039/c7sc05019d (PMC5914134; doi:10.1039/c7sc05019d)
Supplement: Supplementary file 1 [file SC-009-C7SC05019D-s001.pdf]

Electronic Supplementary Material (ESI) for Chemical Science.
This journal is © The Royal Society of Chemistry 2018

Supporting Information

Is the kernel-staples match a key-lock match?

Shengli Zhuang,[+a,b] Lingwen Liao,[+a] Yan Zhao,[+a,b] Jinyun Yuan,[c] Chuanhao Yao,[a] Xu Liu,[a,b] Jin Li,[d] Haiteng Deng,[e] Jinlong Yang[c] and Zhikun Wu*[a]

[a] Key Laboratory of Materials Physics, Anhui Key Laboratory of Nanomaterials and Nanotechnology, Institute of Solid State Physics, Chinese Academy of Sciences, Hefei, Anhui 230031, P.R.China

Institute of Physical Science and Information Technology, Anhui University, Hefei, Anhui 230601, P. R. China

E-mail: zkwu@issp.ac.cn

[b] Department of Chemistry, University of Science and Technology of China, Hefei, Anhui 230026, P.R.China

[c] Hefei National Laboratory for Physics Sciences at the Microscale, University of Science and Technology of China, Hefei, Anhui 230026, P.R. China

[d] Tsinghua University-Peking University Joint Center for Life Sciences, School of Life Sciences, Tsinghua University, Beijing 100084, P.R.China

[e] MOE Key Laboratory of Bioinformatics, School of Life Sciences, Tsinghua University, Beijing 100084, P.R.China

[+] S. Zhuang, L. Liao and Y. Zhao contributed equally to this work.

1. Experimental

1.1 Chemicals. All chemicals are commercially available and were used as received. Tetrachloroauric(III) acid ($HAuCl_4 \cdot 4H_2O$, >99.99% metals basis), tetraoctylammonium bromide (TOAB, ≥98%), 4-tert-butylbenzenethiol (TBBTH, 97%), and sodium borohydride ($NaBH_4$) were purchased from Sigma-Aldrich. Solvents: ethyl acetate (THF, 99.0%), toluene (C_7H_8, 99.5%), acetonitrile (CH_3CN, 99.0%), methanol (CH_3OH, 99.5%), dichloromethane (CH_2Cl_2, 99.0%), petroleum ether (AR), and acetic acid (AR, 99.50%) were purchased from Sinopharm Chemical Reagent Co., ltd. The water used in all experiments was ultrapure (resistivity 18.2 MΩ cm), produced by a Milli-Q NANO pure water system.

1.2 Synthesis of $Au_{42}(TBBT)_{26}$. Tetraoctylammonium bromide (TOAB, 200 mg) was dissolved in 15 mL of ethyl acetate in a tri-neck flask, and $HAuCl_4 \cdot 4H_2O$ (100 mg, 0.243 mmol) was added to the solution. After the solution was stirred for 10 mins, 200 μL of 4-tert-butylbenzenethiol (TBBTH, 6.1 equivs./mol of Au) was added. The solution was kept under constant stirring (900 rpm) until the solution color changed from deep red to yellow, acetic acid-water (200 μL of acetic acid dissolved in 800 μL of water) was added, and then a freshly made cold water solution of $NaBH_4$ (130 mg, 14.1 equivs./mol of Au) was added all at once. The growth of gold nanoclusters was allowed to proceed for 2 h. After removing the precipitates, the concentrated solution was added to cold water, and the crude products were collected and thoroughly washed with methanol. The $Au_{42}(TBBT)_{26}$ nanoclusters were separated by preparative thin-layer chromatography (PTLC, CH_2Cl_2–petroleum ether as developing solvent). The whole process was performed at room temperature without avoiding light or air. Synthesis of $Au_{44}(TBB)_{28}$ nanocluster was referred to the previous work[1].

1.3 Characterization. A Waters Q-TOF mass spectrometer with a Z-spray source was used to collect the electrospray ionization (ESI) mass spectra. The source temperature was kept at 70 °C, the spray voltage was adjusted to 2.20 kV and the cone voltage was kept at 60 V. Nanoclusters weredissolved in toluene (~0.5 mg/mL) and then diluted (1:1 v/v) with methanol solution, which contained 50 mM CsOAc. The prepared sample was directly infused into the chamber at 5 μL/min. The UV/vis/NIR absorption spectra were collected by a UV2600 spectrophotometer in the range of 190-900 nm at room temperature. The single-crystal X-ray diffraction data were collected on a Bruker D_8 VENTURE CMOS photon 100 diffractometer with helios mx multilayer monochromator Mo Kα radiation (λ = 1.54178 Å). X-ray Photoelectron Spectroscopy (XPS) measurements were conducted on an ESCALAB 250Xi XPS spectrometer (Thermo Scientific, America), using a monochromatized Al Kα source and equipped with an Ar^+ ion sputtering gun. All binding energies were calibrated using the C (1s) carbon peak (284.8 eV). The fluorescent properties of the clusters in THF were recorded on Fluorolog-3-21 (Horiba Jobin Yvon, Japan) with a xenon lamp as the excitation source, the step and dwell times were set at 1 nm and 0.1 s, respectively, and the excitation wavelength was kept at 514 nm, with a slit of 10 nm. The luminescence decay profile was obtained under a lifetime fluorescence

spectrometer (DeltaFlex, Horiba Jobin Yvon, France).

1.4 Reference

(1) C. Zeng, Y. Chen, K. Iida, K. Nobusada, K. Kirschbaum, K. J. Lambright and R. Jin, *J Am Chem Soc*, 2016, 138, 3950-3953.

2. Figures

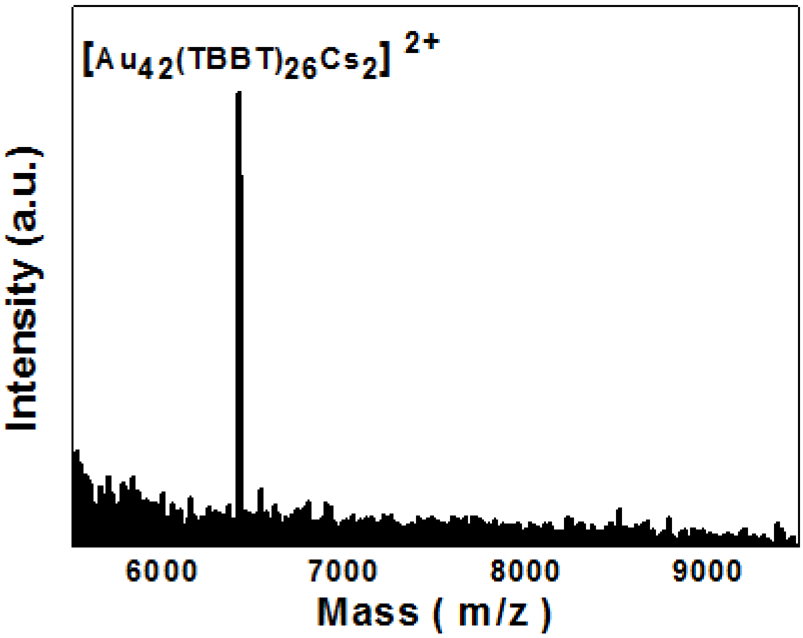

Figure S1. ESI mass spectrum of $Au_{42}(TBBT)_{26}$.

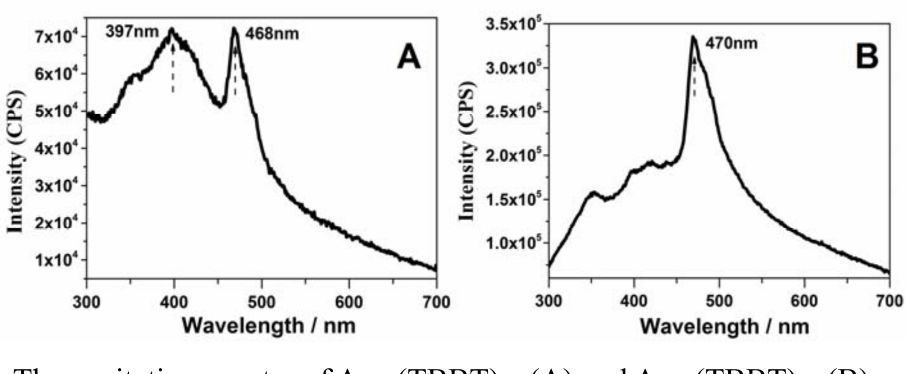

Figure S2. The excitation spectra of $Au_{42}(TBBT)_{26}$ (A) and $Au_{44}(TBBT)_{28}$ (B).

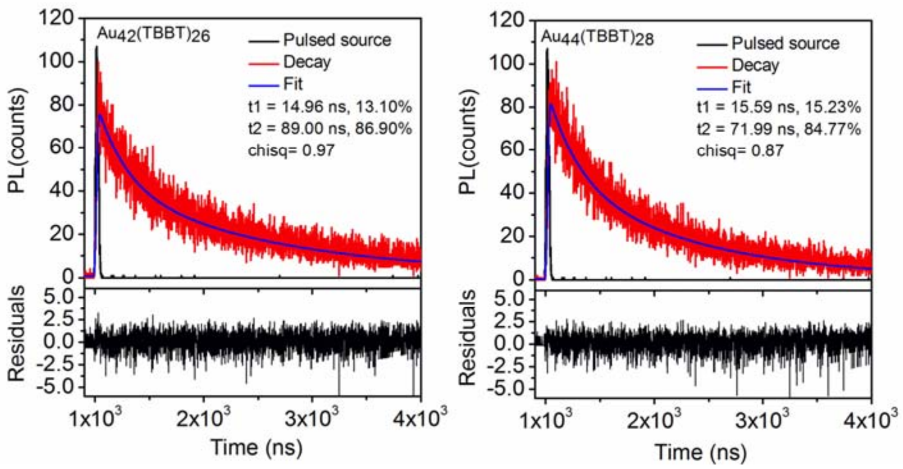

Figure S3. Fluorescence decay profiles of $Au_{42}(TBBT)_{26}$ and $Au_{44}(TBBT)_{28}$ nanoclusters.

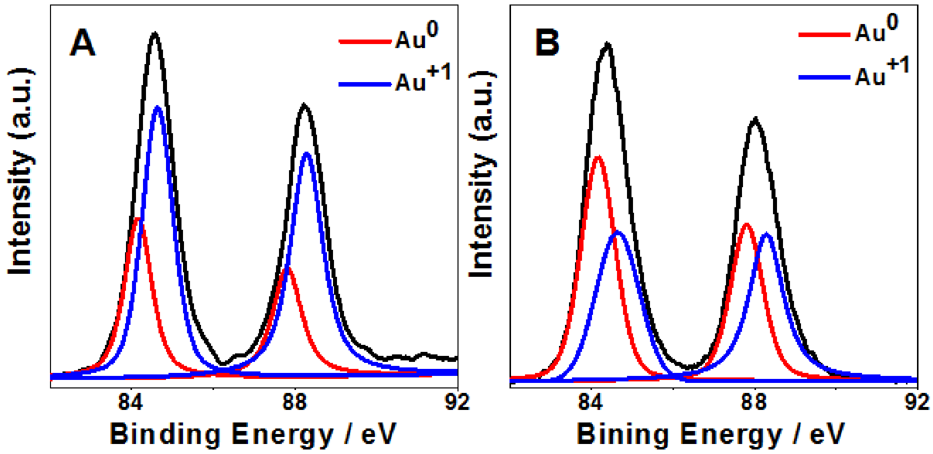

Figure S4. XPS spectra of Au_{4f} for $Au_{42}(TBBT)_{26}$ (A) and $Au_{44}(TBBT)_{28}$ (B).

3. Tables

Table 1. Crystal data and structure refinement for $Au_{42}(TBBT)_{26}$.

Identification code	$Au_{42}(TBBT)_{26}$	
Empirical formula	$C_{260} H_{338} Au_{42} S_{26}$	
Formula weight	12569.44	
Temperature	173 K	
Wavelength	0.71073 Å	
Crystal system	Triclinic	
Space group	P - 1	
Unit cell dimensions	a = 24.328(2) Å	α= 67.732(3)°.
	b = 24.845(3) Å	β= 76.940(2)°.
	c = 32.217(3) Å	γ = 84.496(3)°.
Volume	17553(3) $Å^3$	
Z	2	
Density (calculated)	2.378 Mg/m^3	
Absorption coefficient	17.661 mm^{-1}	
F(000)	11264	
Crystal size	0.12 x 0.15 x 0.15 mm^3	
Theta range for data collection	2.05 to 28.40°.	
Index ranges	-32<=h<=30, -33<=k<=30, -42<=l<=27	
Reflections collected	158151	
Independent reflections	86773 [R(int) = 0.0708]	
Completeness to theta = 25.242°	99.1 %	
Absorption correction	Semi-empirical from equivalents	
Max. and min. transmission	0.7456 and 0.233	
Refinement method	Full-matrix-block least-squares on F^2	
Data / restraints / parameters	86773 / 1707 / 3029	
Goodness-of-fit on F^2	0.976	
Final R indices [I>2sigma(I)]	R1 = 0.0619, wR_2 = 0.1493	
R indices (all data)	R1 = 0.1144, wR_2 = 0.1764	
Extinction coefficient	n/a	
Largest diff. peak and hole	4.854 and -5.691 e.$Å^{-3}$	

Table 2. Atomic coordinates (x 10^4) and equivalent isotropic displacement parameters ($Å^2$x 10^3) for $Au_{42}(TBBT)_{26}$. U(eq) is defined as one third of the trace of the orthogonalized U^{ij} tensor.

	x	y	z	U(eq)
Au(1)	2151(1)	4738(1)	2435(1)	17
Au(2)	2218(1)	5624(1)	2823(1)	21
Au(3)	3097(1)	4697(1)	2819(1)	16
Au(4)	3846(1)	4726(1)	2041(1)	18
Au(5)	2846(1)	4867(1)	1618(1)	19
Au(6)	1974(1)	5707(1)	1724(1)	24
Au(7)	2963(1)	5666(1)	1986(1)	19
Au(8)	4124(1)	5780(1)	2148(1)	22
Au(9)	4155(1)	4757(1)	3243(1)	22
Au(10)	3247(1)	5621(1)	3018(1)	22
Au(11)	2430(1)	4764(1)	3604(1)	20
Au(12)	3154(1)	3735(1)	3725(1)	18
Au(13)	3082(1)	2912(1)	3350(1)	16
Au(14)	3058(1)	3827(1)	2433(1)	14
Au(15)	2162(1)	3787(1)	2022(1)	19
Au(16)	1359(1)	3870(1)	2817(1)	19
Au(17)	1416(1)	4647(1)	3227(1)	19
Au(18)	1043(1)	5732(1)	2576(1)	24
Au(19)	1132(1)	4820(1)	1939(1)	24
Au(20)	2238(1)	3792(1)	3292(1)	15
Au(21)	2239(1)	2930(1)	2861(1)	16
Au(22)	1410(1)	2797(1)	3620(1)	20
Au(23)	1620(1)	1966(1)	3189(1)	21
Au(24)	2247(1)	1979(1)	3785(1)	22
Au(25)	2324(1)	2912(1)	4126(1)	19
Au(26)	1749(1)	3750(1)	4551(1)	23
Au(27)	3035(1)	1879(1)	4457(1)	23
Au(28)	4118(1)	2897(1)	3820(1)	23
Au(29)	3796(1)	2003(1)	3537(1)	22
Au(30)	3092(1)	2008(1)	2992(1)	20
Au(31)	3907(1)	2833(1)	2629(1)	20
Au(32)	3939(1)	3871(1)	2858(1)	18
Au(33)	4507(1)	3881(1)	1642(1)	24

Au(34)	2384(1)	1858(1)	2366(1)	24
Au(35)	2995(1)	2953(1)	2090(1)	19
Au(36)	3473(1)	3815(1)	1164(1)	22
Au(37)	1304(1)	2829(1)	2352(1)	23
Au(38)	3499(1)	698(1)	3951(1)	25
Au(39)	2013(1)	630(1)	3723(1)	25
Au(40)	767(1)	3710(1)	4061(1)	24
Au(41)	2972(1)	6955(1)	2489(1)	29
Au(42)	2000(1)	7010(1)	1695(1)	31
S(1)	3575(2)	6483(2)	1700(1)	20
S(2)	4769(2)	5201(2)	2557(1)	26
S(3)	1517(2)	6374(2)	2726(1)	24
S(4)	474(2)	5197(2)	2401(1)	27
S(5)	1757(2)	4496(2)	1431(1)	24
S(6)	1906(2)	4723(2)	4336(1)	25
S(7)	1636(2)	2755(2)	4831(1)	24
S(8)	3613(2)	2475(2)	4564(1)	22
S(9)	4696(2)	3227(2)	3103(1)	26
S(10)	4602(2)	2884(2)	1963(1)	23
S(11)	4501(2)	4890(2)	1324(1)	22
S(12)	2937(2)	1202(2)	2819(1)	23
S(13)	3392(2)	4812(2)	918(1)	23
S(14)	3598(2)	2814(2)	1429(1)	24
S(15)	1818(2)	2392(2)	1864(1)	26
S(16)	682(2)	3236(2)	2812(1)	23
S(17)	4276(2)	1179(2)	3944(1)	25
S(18)	2754(2)	116(2)	4049(1)	31
S(19)	1250(2)	1036(2)	3390(1)	25
S(20)	2449(2)	1208(2)	4445(1)	23
S(21)	619(2)	2740(2)	4231(1)	25
S(22)	769(2)	4708(2)	3888(1)	25
S(23)	3590(2)	4361(2)	3966(1)	22
S(24)	3560(2)	6502(2)	2995(1)	27
S(25)	2416(2)	7536(2)	1994(1)	32
S(26)	1549(2)	6592(2)	1338(1)	30
C(1)	1158(5)	6366(6)	3281(4)	16
C(2)	707(6)	6771(7)	3301(5)	25
C(3)	425(8)	6787(9)	3726(6)	43

C(4)	580(7)	6422(8)	4120(5)	33
C(5)	1024(7)	6050(8)	4103(6)	33
C(6)	1324(7)	6045(8)	3678(5)	30
C(7)	209(8)	6435(10)	4594(7)	50
C(8)	466(8)	6061(9)	4977(6)	52
C(9)	-352(9)	6221(11)	4648(8)	75
C(10)	193(13)	7046(14)	4603(10)	117
C(11)	1389(6)	4192(7)	1164(5)	23
C(12)	906(7)	4441(9)	1024(6)	40
C(13)	664(9)	4226(10)	765(7)	54
C(14)	892(9)	3759(10)	650(7)	55
C(15)	1385(7)	3515(9)	789(6)	40
C(16)	1624(6)	3725(7)	1050(5)	28
C(17)	595(11)	3471(13)	369(9)	77
C(18)	23(11)	3182(13)	715(9)	98
C(19)	408(11)	3941(12)	1(8)	92
C(20)	862(16)	2933(17)	311(13)	166
C(21)	88(7)	5751(8)	2040(6)	31
C(22)	173(9)	5903(10)	1567(7)	55
C(23)	-161(9)	6354(11)	1310(8)	69
C(24)	-575(15)	6627(15)	1530(12)	141
C(25)	-671(9)	6473(10)	1997(7)	59
C(26)	-325(9)	6041(10)	2259(8)	63
C(27)	-950(11)	7072(12)	1237(9)	156
C(28)	-634(12)	7434(15)	760(10)	174
C(29)	-1427(13)	6811(13)	1142(11)	164
C(30)	-1231(14)	7502(14)	1452(10)	179
C(31)	2411(6)	4750(7)	4653(5)	20
C(32)	2448(6)	4318(7)	5067(5)	26
C(33)	2834(6)	4368(8)	5314(6)	32
C(34)	3198(6)	4830(7)	5151(5)	26
C(35)	3164(7)	5268(8)	4723(6)	36
C(36)	2765(6)	5230(7)	4485(5)	22
C(37)	3645(7)	4888(8)	5390(6)	38
C(38)	3553(7)	5450(8)	5492(6)	37
C(39)	3642(7)	4381(8)	5854(6)	41
C(40)	4223(7)	4904(9)	5092(6)	43
C(41)	1922(6)	2490(7)	5347(5)	20

C(42)	2203(6)	1980(8)	5478(5)	30
C(43)	2361(6)	1761(8)	5910(5)	30
C(44)	2277(7)	2065(8)	6187(5)	31
C(45)	1975(8)	2582(9)	6060(6)	45
C(46)	1801(7)	2789(8)	5654(6)	37
C(47)	2480(9)	1816(10)	6643(7)	53
C(48)	2292(10)	2162(11)	6939(8)	78
C(49)	3110(10)	1810(13)	6538(9)	93
C(50)	2269(11)	1216(12)	6904(9)	95
C(51)	5246(6)	3647(7)	3091(5)	19
C(52)	5581(7)	3964(8)	2663(5)	33
C(53)	6030(7)	4284(8)	2631(6)	32
C(54)	6177(6)	4304(7)	3014(5)	26
C(55)	5847(7)	4006(8)	3424(6)	35
C(56)	5395(7)	3685(8)	3466(6)	34
C(57)	6672(8)	4659(9)	2989(6)	45
C(58)	6962(8)	4981(9)	2499(6)	53
C(59)	6474(8)	5114(9)	3199(7)	52
C(60)	7097(8)	4244(10)	3236(7)	60
C(61)	5178(6)	5141(7)	1334(5)	24
C(62)	5623(6)	4756(8)	1440(5)	28
C(63)	6150(6)	4983(7)	1363(5)	28
C(64)	6276(7)	5552(8)	1160(5)	29
C(65)	5784(6)	5930(7)	1084(5)	26
C(66)	5268(7)	5709(8)	1164(5)	29
C(67)	6850(8)	5813(9)	1018(6)	44
C(68)	6982(8)	6113(9)	471(6)	50
C(69)	7299(7)	5327(9)	1127(6)	47
C(70)	6906(8)	6231(10)	1232(7)	58
C(71)	5295(6)	2824(7)	2075(5)	21
C(72)	5744(7)	3120(8)	1732(6)	36
C(73)	6269(6)	3062(7)	1800(5)	27
C(74)	6408(7)	2741(9)	2223(6)	45
C(75)	5976(7)	2406(8)	2564(6)	37
C(76)	5432(6)	2445(7)	2491(5)	24
C(77)	7007(7)	2689(9)	2296(6)	58
C(78)	7132(10)	2165(10)	2700(8)	88
C(79)	7400(10)	2531(12)	1915(8)	93

C(80)	7158(11)	3258(10)	2283(10)	104
C(81)	2890(6)	5061(7)	559(5)	21
C(82)	2727(7)	5653(8)	453(5)	31
C(83)	2336(7)	5895(8)	183(6)	37
C(84)	2087(6)	5537(8)	21(5)	28
C(85)	2247(6)	4974(7)	119(5)	28
C(86)	2668(6)	4720(8)	393(5)	29
C(87)	1629(9)	5825(11)	-269(8)	63
C(88)	1383(8)	5380(9)	-406(7)	52
C(89)	1150(9)	6045(11)	36(8)	68
C(90)	1920(10)	6319(11)	-710(8)	73
C(91)	3211(6)	2517(7)	1160(5)	21
C(92)	2977(6)	2867(8)	789(5)	32
C(93)	2723(7)	2582(8)	578(6)	36
C(94)	2715(7)	1999(8)	693(6)	41
C(95)	2947(7)	1650(8)	1074(5)	32
C(96)	3196(6)	1926(8)	1297(5)	30
C(97)	2472(8)	1697(8)	440(6)	54
C(98)	1954(9)	1364(11)	736(8)	87
C(99)	2911(11)	1298(12)	280(10)	112
C(100)	2293(11)	2123(11)	4(7)	92
C(101)	176(6)	3659(7)	2488(5)	18
C(102)	162(7)	3631(8)	2073(6)	39
C(103)	-284(7)	3963(9)	1856(6)	45
C(104)	-668(7)	4304(8)	2034(5)	32
C(105)	-619(7)	4288(8)	2460(5)	32
C(106)	-223(7)	3985(8)	2691(6)	36
C(107)	-1129(7)	4633(8)	1777(6)	35
C(108)	-842(7)	5023(9)	1274(6)	48
C(109)	-1431(7)	5077(9)	1979(6)	47
C(110)	-1530(8)	4216(10)	1786(7)	54
C(111)	1362(7)	1849(8)	1856(6)	35
C(112)	1471(10)	1263(12)	2053(8)	75
C(113)	1116(10)	904(13)	2023(8)	80
C(114)	679(11)	1071(13)	1788(9)	83
C(115)	591(8)	1633(10)	1602(7)	49
C(116)	935(8)	2077(10)	1628(6)	50
C(117)	302(13)	658(15)	1752(11)	100

C(118)	-92(13)	895(14)	1438(10)	127
C(119)	48(13)	210(15)	2232(10)	124
C(120)	679(13)	237(14)	1579(11)	127
C(121)	3504(6)	1078(7)	2394(5)	27
C(122)	3987(6)	1371(7)	2221(5)	23
C(123)	4402(7)	1269(7)	1889(5)	30
C(124)	4328(7)	824(8)	1737(6)	34
C(125)	3832(7)	540(8)	1910(6)	37
C(126)	3406(7)	650(8)	2238(5)	33
C(127)	4746(8)	708(9)	1330(6)	43
C(128)	5317(9)	1050(11)	1216(7)	68
C(129)	4887(9)	66(9)	1469(7)	58
C(130)	4474(9)	916(10)	923(6)	59
C(131)	655(6)	990(7)	3860(5)	19
C(132)	130(6)	882(8)	3801(6)	33
C(133)	-338(7)	820(8)	4163(5)	34
C(134)	-290(7)	900(8)	4549(6)	38
C(135)	236(7)	1001(8)	4605(6)	35
C(136)	704(7)	1041(7)	4255(5)	30
C(137)	-817(7)	832(9)	4946(6)	40
C(138)	-653(9)	552(10)	5408(7)	60
C(139)	-1258(9)	476(11)	4925(8)	71
C(140)	-1025(10)	1432(11)	4896(8)	88
C(141)	3008(6)	-332(7)	3717(5)	24
C(142)	3523(6)	-186(7)	3390(5)	24
C(143)	3738(7)	-581(8)	3153(6)	33
C(144)	3434(7)	-1044(8)	3223(6)	38
C(145)	2939(8)	-1164(10)	3529(7)	55
C(146)	2736(8)	-817(9)	3780(6)	46
C(147)	3683(8)	-1479(10)	2964(7)	51
C(148)	3227(9)	-1864(10)	2990(7)	64
C(149)	3869(9)	-1091(10)	2455(6)	59
C(150)	4163(9)	-1830(11)	3181(8)	71
C(151)	4829(6)	1020(7)	3537(5)	19
C(152)	5367(6)	921(7)	3621(5)	27
C(153)	5806(8)	795(8)	3322(6)	41
C(154)	5731(8)	746(9)	2915(6)	44
C(155)	5173(7)	840(8)	2833(6)	39

C(156)	4738(7)	1000(8)	3133(5)	34
C(157)	6200(9)	577(11)	2588(7)	58
C(158)	6280(10)	1114(11)	2132(7)	75
C(159)	6741(9)	470(11)	2732(8)	79
C(160)	6033(11)	47(12)	2530(9)	94
C(161)	4095(6)	2012(7)	4882(5)	21
C(162)	4655(6)	1931(7)	4680(5)	30
C(163)	5016(7)	1570(8)	4961(6)	39
C(164)	4834(7)	1279(8)	5430(6)	34
C(165)	4258(7)	1373(8)	5631(6)	42
C(166)	3911(6)	1732(7)	5348(5)	24
C(167)	5251(7)	906(8)	5722(6)	40
C(168)	5482(9)	440(10)	5520(7)	58
C(169)	5729(8)	1305(9)	5694(7)	55
C(170)	4975(8)	611(9)	6220(6)	47
C(171)	1871(6)	1095(7)	4932(5)	28
C(172)	1947(6)	658(7)	5338(5)	26
C(173)	1546(8)	593(9)	5737(6)	46
C(174)	1065(7)	952(8)	5741(6)	38
C(175)	1007(7)	1360(8)	5330(5)	31
C(176)	1399(7)	1458(8)	4923(6)	34
C(177)	623(8)	924(10)	6193(7)	48
C(178)	198(9)	1359(11)	6141(8)	75
C(179)	315(10)	356(11)	6356(8)	83
C(180)	942(11)	884(13)	6539(9)	103
C(181)	102(6)	4941(7)	3739(5)	21
C(182)	-112(7)	5463(8)	3741(6)	39
C(183)	-646(7)	5646(9)	3664(6)	43
C(184)	-963(7)	5324(8)	3548(6)	33
C(185)	-739(7)	4792(8)	3512(5)	33
C(186)	-223(6)	4617(8)	3608(5)	30
C(187)	-1592(8)	5459(9)	3505(6)	44
C(188)	-1985(8)	5033(9)	3904(6)	53
C(189)	-1702(9)	5445(10)	3057(7)	64
C(190)	-1756(8)	6081(9)	3514(7)	59
C(191)	5(6)	2749(7)	4021(5)	22
C(192)	-43(7)	2389(8)	3796(5)	32
C(193)	-540(6)	2372(7)	3653(5)	29

C(194)	-999(8)	2707(9)	3722(6)	47
C(195)	-951(7)	3066(8)	3951(6)	40
C(196)	-459(6)	3079(7)	4105(5)	26
C(197)	-1540(9)	2685(11)	3560(7)	58
C(198)	-1399(10)	2812(12)	3055(8)	81
C(199)	-1763(11)	2090(12)	3753(9)	94
C(200)	-1982(11)	3122(13)	3658(9)	99
C(201)	4027(6)	3991(6)	4366(4)	16
C(202)	3773(7)	3605(7)	4785(5)	28
C(203)	4072(6)	3312(7)	5125(5)	24
C(204)	4644(6)	3417(7)	5052(5)	25
C(205)	4900(6)	3796(7)	4628(5)	28
C(206)	4590(6)	4086(7)	4293(5)	29
C(207)	5010(7)	3109(8)	5426(5)	32
C(208)	5149(7)	3562(8)	5594(6)	36
C(209)	5544(7)	2870(9)	5200(6)	43
C(210)	4681(7)	2615(8)	5837(6)	43
C(211)	1945(6)	6945(8)	764(5)	30
C(212)	2526(7)	6943(8)	649(5)	31
C(213)	2818(7)	7231(8)	219(5)	32
C(214)	2532(8)	7533(9)	-130(6)	46
C(215)	1945(8)	7525(9)	-15(7)	50
C(216)	1647(7)	7209(8)	432(5)	32
C(217)	2889(9)	7854(11)	-627(8)	63
C(218)	3234(10)	7451(11)	-800(8)	84
C(219)	2488(11)	8160(13)	-950(9)	98
C(220)	3236(10)	8324(12)	-619(8)	87
C(221)	2920(6)	7998(7)	1538(5)	27
C(222)	2991(9)	8021(10)	1090(7)	57
C(223)	3380(9)	8402(10)	747(7)	59
C(224)	3677(11)	8770(12)	827(9)	89
C(225)	3640(8)	8743(9)	1279(7)	52
C(226)	3232(7)	8368(8)	1618(6)	39
C(227)	4082(10)	9193(11)	424(8)	102
C(228)	3879(13)	9483(14)	-21(9)	126
C(229)	4240(13)	9704(13)	527(11)	126
C(230)	3198(6)	6587(7)	3505(5)	22
C(231)	3514(7)	6689(8)	3793(5)	32

C(232)	3265(8)	6807(9)	4164(6)	44
C(233)	2674(7)	6870(8)	4283(6)	34
C(234)	2347(8)	6781(9)	3992(6)	42
C(235)	2613(6)	6677(7)	3607(5)	27
C(236)	2379(9)	7001(10)	4708(7)	57
C(237)	2080(10)	6418(11)	5069(8)	78
C(238)	2778(11)	7145(13)	4926(9)	97
C(239)	1922(11)	7456(13)	4609(10)	105
C(240)	5147(6)	5728(7)	2622(5)	24
C(241)	5040(7)	5887(8)	3002(6)	33
C(242)	5346(6)	6324(7)	3017(5)	29
C(243)	5768(7)	6614(8)	2659(6)	37
C(244)	5871(7)	6461(9)	2270(6)	40
C(245)	5588(7)	6028(8)	2262(6)	37
C(246)	6095(9)	7077(10)	2683(7)	53
C(247)	6427(12)	6807(14)	3053(10)	124
C(248)	6507(11)	7390(13)	2222(9)	102
C(249)	5721(13)	7525(15)	2778(11)	132
C(250)	3928(6)	6606(8)	1122(5)	30
C(251)	3869(5)	6234(6)	892(4)	17
C(252)	4183(6)	6326(7)	468(5)	25
C(253)	4576(6)	6779(7)	242(5)	28
C(254)	4623(7)	7121(8)	465(5)	30
C(255)	4303(6)	7052(8)	897(5)	32
C(256)	4982(8)	6858(9)	-227(6)	46
C(257)	4889(10)	6361(11)	-375(8)	71
C(258)	4917(14)	7393(15)	-569(11)	150
C(259)	5567(11)	6727(14)	-163(10)	113
C(260)	4634(11)	8877(13)	336(10)	117

Table 3. Bond lengths [Å] and angles [°] for $Au_{42}(TBBT)_{26}$.

Au(1)-Au(2)	2.9463(9)
Au(1)-Au(3)	2.8265(7)
Au(1)-Au(5)	2.7095(7)
Au(1)-Au(6)	2.7013(8)
Au(1)-Au(7)	2.8940(8)
Au(1)-Au(14)	3.0056(8)
Au(1)-Au(15)	3.1157(9)
Au(1)-Au(16)	2.7525(8)
Au(1)-Au(17)	2.7076(7)
Au(1)-Au(19)	3.1898(8)
Au(1)-Au(20)	2.9047(7)
Au(2)-Au(3)	2.9896(8)
Au(2)-Au(7)	2.8601(8)
Au(2)-Au(10)	2.7156(8)
Au(2)-Au(11)	2.7340(8)
Au(2)-Au(17)	2.9590(9)
Au(2)-Au(18)	3.0950(8)
Au(2)-S(3)	2.379(4)
Au(3)-Au(4)	2.7269(7)
Au(3)-Au(7)	2.9080(8)
Au(3)-Au(9)	3.2136(8)
Au(3)-Au(10)	2.6805(9)
Au(3)-Au(11)	2.7320(8)
Au(3)-Au(12)	3.0132(8)
Au(3)-Au(14)	2.8923(9)
Au(3)-Au(20)	2.9349(8)
Au(3)-Au(32)	2.7477(8)
Au(4)-Au(5)	2.9795(7)
Au(4)-Au(7)	2.9998(8)
Au(4)-Au(8)	2.9223(10)
Au(4)-Au(14)	2.8027(8)
Au(4)-Au(32)	2.7253(8)
Au(4)-Au(33)	3.0179(9)
Au(4)-S(11)	2.407(3)
Au(5)-Au(6)	2.8855(9)
Au(5)-Au(7)	2.7322(9)

Au(5)-Au(14)	3.0074(8)
Au(5)-Au(15)	2.9824(9)
Au(5)-S(13)	2.392(4)
Au(6)-Au(7)	2.7034(8)
Au(6)-Au(18)	3.1568(8)
Au(6)-Au(19)	2.9336(9)
Au(6)-Au(42)	3.2100(11)
Au(6)-S(26)	2.352(4)
Au(7)-Au(8)	3.0422(8)
Au(7)-S(1)	2.392(4)
Au(8)-Au(10)	3.0307(8)
Au(8)-S(1)	2.329(4)
Au(8)-S(2)	2.311(4)
Au(9)-Au(10)	2.9394(8)
Au(9)-Au(12)	3.3574(9)
Au(9)-Au(32)	3.0409(9)
Au(9)-S(2)	2.303(4)
Au(9)-S(23)	2.315(4)
Au(10)-Au(11)	2.8494(9)
Au(10)-Au(41)	3.1853(10)
Au(10)-S(24)	2.356(5)
Au(11)-Au(12)	2.9119(8)
Au(11)-Au(17)	3.0687(8)
Au(11)-Au(20)	3.0568(9)
Au(11)-Au(26)	3.3304(9)
Au(11)-S(6)	2.383(4)
Au(12)-Au(13)	2.7753(9)
Au(12)-Au(20)	2.8519(7)
Au(12)-Au(25)	2.7477(9)
Au(12)-Au(28)	2.9720(8)
Au(12)-Au(32)	2.9260(8)
Au(12)-S(23)	2.398(4)
Au(13)-Au(14)	2.9781(7)
Au(13)-Au(20)	2.8323(8)
Au(13)-Au(21)	2.8414(7)
Au(13)-Au(24)	2.9353(9)
Au(13)-Au(25)	2.7504(7)
Au(13)-Au(28)	3.2118(8)

Au(13)-Au(29)	2.6792(8)
Au(13)-Au(30)	2.8848(9)
Au(13)-Au(31)	2.7624(8)
Au(13)-Au(32)	3.0235(9)
Au(14)-Au(15)	2.8212(7)
Au(14)-Au(20)	2.9987(7)
Au(14)-Au(21)	2.8498(8)
Au(14)-Au(31)	3.0334(8)
Au(14)-Au(32)	2.8237(7)
Au(14)-Au(35)	2.8136(9)
Au(15)-Au(16)	2.9119(8)
Au(15)-Au(21)	2.7710(8)
Au(15)-Au(35)	2.7382(8)
Au(15)-Au(37)	3.0313(9)
Au(15)-S(5)	2.381(4)
Au(16)-Au(17)	2.7488(9)
Au(16)-Au(19)	3.0428(8)
Au(16)-Au(20)	2.8497(7)
Au(16)-Au(21)	2.9985(8)
Au(16)-Au(22)	2.9450(8)
Au(16)-S(16)	2.393(4)
Au(17)-Au(18)	2.9294(9)
Au(17)-Au(20)	2.7572(8)
Au(17)-Au(40)	3.0302(9)
Au(17)-S(22)	2.392(4)
Au(18)-S(3)	2.300(4)
Au(18)-S(4)	2.297(4)
Au(19)-S(4)	2.314(4)
Au(19)-S(5)	2.297(4)
Au(20)-Au(21)	2.9591(9)
Au(20)-Au(22)	3.0497(9)
Au(20)-Au(25)	2.7860(8)
Au(21)-Au(22)	2.7277(7)
Au(21)-Au(23)	2.6781(9)
Au(21)-Au(24)	3.0198(8)
Au(21)-Au(30)	2.9106(8)
Au(21)-Au(35)	2.7202(7)
Au(21)-Au(37)	3.1690(8)

Au(22)-Au(23)	2.8451(9)
Au(22)-Au(24)	2.7265(8)
Au(22)-Au(25)	3.1309(8)
Au(22)-Au(40)	3.2196(9)
Au(22)-S(21)	2.394(4)
Au(23)-Au(24)	2.7188(8)
Au(23)-Au(34)	2.9627(8)
Au(23)-Au(37)	2.9495(8)
Au(23)-Au(39)	3.2705(9)
Au(23)-S(19)	2.359(4)
Au(24)-Au(25)	2.9528(9)
Au(24)-Au(27)	3.1324(8)
Au(24)-Au(30)	2.8764(8)
Au(24)-S(20)	2.374(4)
Au(25)-Au(26)	2.9848(9)
Au(25)-Au(27)	2.9367(8)
Au(25)-S(7)	2.417(4)
Au(26)-Au(40)	3.1762(8)
Au(26)-S(6)	2.294(5)
Au(26)-S(7)	2.309(4)
Au(27)-Au(29)	3.0425(8)
Au(27)-S(8)	2.300(4)
Au(27)-S(20)	2.313(4)
Au(28)-Au(29)	2.9276(10)
Au(28)-Au(32)	3.2220(8)
Au(28)-S(8)	2.315(4)
Au(28)-S(9)	2.302(4)
Au(29)-Au(30)	2.7133(8)
Au(29)-Au(31)	2.8382(8)
Au(29)-Au(38)	3.0898(10)
Au(29)-S(17)	2.339(4)
Au(30)-Au(31)	2.7229(9)
Au(30)-Au(34)	3.0724(8)
Au(30)-Au(35)	3.0066(8)
Au(30)-S(12)	2.355(4)
Au(31)-Au(32)	2.9510(9)
Au(31)-Au(33)	3.3731(9)
Au(31)-Au(35)	3.0412(8)

Au(31)-S(10)	2.381(4)
Au(32)-S(9)	2.388(4)
Au(33)-Au(36)	3.2799(8)
Au(33)-S(10)	2.307(4)
Au(33)-S(11)	2.321(4)
Au(34)-Au(35)	2.9582(10)
Au(34)-S(12)	2.294(4)
Au(34)-S(15)	2.302(4)
Au(35)-Au(36)	2.9854(8)
Au(35)-S(14)	2.420(4)
Au(36)-S(13)	2.299(4)
Au(36)-S(14)	2.318(4)
Au(37)-S(15)	2.313(4)
Au(37)-S(16)	2.311(4)
Au(38)-S(17)	2.323(4)
Au(38)-S(18)	2.318(5)
Au(39)-S(18)	2.319(4)
Au(39)-S(19)	2.310(4)
Au(40)-S(21)	2.305(5)
Au(40)-S(22)	2.328(5)
Au(41)-S(24)	2.308(4)
Au(41)-S(25)	2.306(4)
Au(42)-S(25)	2.301(5)
Au(42)-S(26)	2.304(5)
S(1)-C(250)	1.789(16)
S(2)-C(240)	1.767(17)
S(3)-C(1)	1.800(13)
S(4)-C(21)	1.778(16)
S(5)-C(11)	1.757(15)
S(6)-C(31)	1.787(14)
S(7)-C(41)	1.815(14)
S(8)-C(161)	1.772(14)
S(9)-C(51)	1.759(16)
S(10)-C(71)	1.782(14)
S(11)-C(61)	1.828(15)
S(12)-C(121)	1.804(16)
S(13)-C(81)	1.777(14)
S(14)-C(91)	1.779(15)

S(15)-C(111)	1.838(19)
S(16)-C(101)	1.792(13)
S(17)-C(151)	1.783(15)
S(18)-C(141)	1.800(16)
S(19)-C(131)	1.821(14)
S(20)-C(171)	1.807(16)
S(21)-C(191)	1.770(14)
S(22)-C(181)	1.776(14)
S(23)-C(201)	1.779(13)
S(24)-C(230)	1.758(15)
S(25)-C(221)	1.777(16)
S(26)-C(211)	1.800(16)
C(1)-C(2)	1.422(19)
C(1)-C(6)	1.354(19)
C(2)-H(2)	0.9300
C(2)-C(3)	1.40(2)
C(3)-H(3)	0.9300
C(3)-C(4)	1.36(2)
C(4)-C(5)	1.36(2)
C(4)-C(7)	1.60(2)
C(5)-H(5)	0.9300
C(5)-C(6)	1.40(2)
C(6)-H(6)	0.9300
C(7)-C(8)	1.47(2)
C(7)-C(9)	1.46(3)
C(7)-C(10)	1.53(3)
C(8)-H(8A)	0.9600
C(8)-H(8B)	0.9600
C(8)-H(8C)	0.9600
C(9)-H(9A)	0.9600
C(9)-H(9B)	0.9600
C(9)-H(9C)	0.9600
C(10)-H(10A)	0.9600
C(10)-H(10B)	0.9600
C(10)-H(10C)	0.9600
C(11)-C(12)	1.36(2)
C(11)-C(16)	1.38(2)
C(12)-H(12)	0.9300

C(12)-C(13)	1.39(3)
C(13)-H(13)	0.9300
C(13)-C(14)	1.38(3)
C(14)-C(15)	1.38(2)
C(14)-C(17)	1.65(3)
C(15)-H(15)	0.9300
C(15)-C(16)	1.39(2)
C(16)-H(16)	0.9300
C(17)-C(18)	1.61(3)
C(17)-C(19)	1.44(3)
C(17)-C(20)	1.49(4)
C(18)-H(18A)	0.9600
C(18)-H(18B)	0.9600
C(18)-H(18C)	0.9600
C(19)-H(19A)	0.9600
C(19)-H(19B)	0.9600
C(19)-H(19C)	0.9600
C(20)-H(20A)	0.9600
C(20)-H(20B)	0.9600
C(20)-H(20C)	0.9600
C(21)-C(22)	1.39(2)
C(21)-C(26)	1.40(3)
C(22)-H(22)	0.9300
C(22)-C(23)	1.42(3)
C(23)-H(23)	0.9300
C(23)-C(24)	1.37(4)
C(24)-C(25)	1.37(4)
C(24)-C(27)	1.533(14)
C(25)-H(25)	0.9300
C(25)-C(26)	1.43(3)
C(26)-H(26)	0.9300
C(27)-C(28)	1.521(14)
C(27)-C(29)	1.523(13)
C(27)-C(30)	1.514(14)
C(28)-H(28A)	0.9600
C(28)-H(28B)	0.9600
C(28)-H(28C)	0.9600
C(29)-H(29A)	0.9600

C(29)-H(29B)	0.9600
C(29)-H(29C)	0.9600
C(30)-H(30A)	0.9600
C(30)-H(30B)	0.9600
C(30)-H(30C)	0.9600
C(31)-C(32)	1.375(19)
C(31)-C(36)	1.40(2)
C(32)-H(32)	0.9300
C(32)-C(33)	1.40(2)
C(33)-H(33)	0.9300
C(33)-C(34)	1.38(2)
C(34)-C(35)	1.41(2)
C(34)-C(37)	1.51(2)
C(35)-H(35)	0.9300
C(35)-C(36)	1.39(2)
C(36)-H(36)	0.9300
C(37)-C(38)	1.54(2)
C(37)-C(39)	1.55(2)
C(37)-C(40)	1.51(2)
C(38)-H(38A)	0.9600
C(38)-H(38B)	0.9600
C(38)-H(38C)	0.9600
C(39)-H(39A)	0.9600
C(39)-H(39B)	0.9600
C(39)-H(39C)	0.9600
C(40)-H(40A)	0.9600
C(40)-H(40B)	0.9600
C(40)-H(40C)	0.9600
C(41)-C(42)	1.34(2)
C(41)-C(46)	1.41(2)
C(42)-H(42)	0.9300
C(42)-C(43)	1.42(2)
C(43)-H(43)	0.9300
C(43)-C(44)	1.34(2)
C(44)-C(45)	1.38(2)
C(44)-C(47)	1.54(2)
C(45)-H(45)	0.9300
C(45)-C(46)	1.36(2)

C(46)-H(46)	0.9300
C(47)-C(48)	1.49(3)
C(47)-C(49)	1.49(3)
C(47)-C(50)	1.48(3)
C(48)-H(48A)	0.9600
C(48)-H(48B)	0.9600
C(48)-H(48C)	0.9600
C(49)-H(49A)	0.9600
C(49)-H(49B)	0.9600
C(49)-H(49C)	0.9600
C(50)-H(50A)	0.9600
C(50)-H(50B)	0.9600
C(50)-H(50C)	0.9600
C(51)-C(52)	1.41(2)
C(51)-C(56)	1.37(2)
C(52)-H(52)	0.9300
C(52)-C(53)	1.38(2)
C(53)-H(53)	0.9300
C(53)-C(54)	1.38(2)
C(54)-C(55)	1.35(2)
C(54)-C(57)	1.53(3)
C(55)-H(55)	0.9300
C(55)-C(56)	1.37(2)
C(56)-H(56)	0.9300
C(57)-C(58)	1.51(3)
C(57)-C(59)	1.51(3)
C(57)-C(60)	1.52(2)
C(58)-H(58A)	0.9600
C(58)-H(58B)	0.9600
C(58)-H(58C)	0.9600
C(59)-H(59A)	0.9600
C(59)-H(59B)	0.9600
C(59)-H(59C)	0.9600
C(60)-H(60A)	0.9600
C(60)-H(60B)	0.9600
C(60)-H(60C)	0.9600
C(61)-C(62)	1.387(19)
C(61)-C(66)	1.33(2)

C(62)-H(62)	0.9300
C(62)-C(63)	1.38(2)
C(63)-H(63)	0.9300
C(63)-C(64)	1.34(2)
C(64)-C(65)	1.46(2)
C(64)-C(67)	1.49(2)
C(65)-H(65)	0.9300
C(65)-C(66)	1.35(2)
C(66)-H(66)	0.9300
C(67)-C(68)	1.60(2)
C(67)-C(69)	1.54(2)
C(67)-C(70)	1.48(3)
C(68)-H(68A)	0.9600
C(68)-H(68B)	0.9600
C(68)-H(68C)	0.9600
C(69)-H(69A)	0.9600
C(69)-H(69B)	0.9600
C(69)-H(69C)	0.9600
C(70)-H(70A)	0.9600
C(70)-H(70B)	0.9600
C(70)-H(70C)	0.9600
C(71)-C(72)	1.40(2)
C(71)-C(76)	1.407(19)
C(72)-H(72)	0.9300
C(72)-C(73)	1.33(2)
C(73)-H(73)	0.9300
C(73)-C(74)	1.40(2)
C(74)-C(75)	1.40(2)
C(74)-C(77)	1.513(16)
C(75)-H(75)	0.9300
C(75)-C(76)	1.38(2)
C(76)-H(76)	0.9300
C(77)-C(78)	1.516(17)
C(77)-C(79)	1.529(17)
C(77)-C(80)	1.479(17)
C(78)-H(78A)	0.9600
C(78)-H(78B)	0.9600
C(78)-H(78C)	0.9600

C(79)-H(79A)	0.9600
C(79)-H(79B)	0.9600
C(79)-H(79C)	0.9600
C(80)-H(80A)	0.9600
C(80)-H(80B)	0.9600
C(80)-H(80C)	0.9600
C(81)-C(82)	1.42(2)
C(81)-C(86)	1.37(2)
C(82)-H(82)	0.9300
C(82)-C(83)	1.37(2)
C(83)-H(83)	0.9300
C(83)-C(84)	1.42(2)
C(84)-C(85)	1.35(2)
C(84)-C(87)	1.56(2)
C(85)-H(85)	0.9300
C(85)-C(86)	1.445(19)
C(86)-H(86)	0.9300
C(87)-C(88)	1.55(3)
C(87)-C(89)	1.55(3)
C(87)-C(90)	1.55(3)
C(88)-H(88A)	0.9600
C(88)-H(88B)	0.9600
C(88)-H(88C)	0.9600
C(89)-H(89A)	0.9600
C(89)-H(89B)	0.9600
C(89)-H(89C)	0.9600
C(90)-H(90A)	0.9600
C(90)-H(90B)	0.9600
C(90)-H(90C)	0.9600
C(91)-C(92)	1.39(2)
C(91)-C(96)	1.37(2)
C(92)-H(92)	0.9300
C(92)-C(93)	1.41(2)
C(93)-H(93)	0.9300
C(93)-C(94)	1.35(2)
C(94)-C(95)	1.41(2)
C(94)-C(97)	1.530(16)
C(95)-H(95)	0.9300

C(95)-C(96)	1.41(2)
C(96)-H(96)	0.9300
C(97)-C(98)	1.497(17)
C(97)-C(99)	1.527(17)
C(97)-C(100)	1.532(16)
C(98)-H(98A)	0.9600
C(98)-H(98B)	0.9600
C(98)-H(98C)	0.9600
C(99)-H(99A)	0.9600
C(99)-H(99B)	0.9600
C(99)-H(99C)	0.9600
C(100)-H(10D)	0.9600
C(100)-H(10E)	0.9600
C(100)-H(10F)	0.9600
C(101)-C(102)	1.37(2)
C(101)-C(106)	1.41(2)
C(102)-H(102)	0.9300
C(102)-C(103)	1.44(2)
C(103)-H(103)	0.9300
C(103)-C(104)	1.37(2)
C(104)-C(105)	1.39(2)
C(104)-C(107)	1.53(2)
C(105)-H(105)	0.9300
C(105)-C(106)	1.35(2)
C(106)-H(106)	0.9300
C(107)-C(108)	1.57(2)
C(107)-C(109)	1.53(2)
C(107)-C(110)	1.48(3)
C(108)-H(10G)	0.9600
C(108)-H(10H)	0.9600
C(108)-H(10I)	0.9600
C(109)-H(10J)	0.9600
C(109)-H(10K)	0.9600
C(109)-H(10L)	0.9600
C(110)-H(11A)	0.9600
C(110)-H(11B)	0.9600
C(110)-H(11C)	0.9600
C(111)-C(112)	1.38(3)

C(111)-C(116)	1.36(2)
C(112)-H(112)	0.9300
C(112)-C(113)	1.34(3)
C(113)-H(113)	0.9300
C(113)-C(114)	1.38(3)
C(114)-C(115)	1.31(3)
C(114)-C(117)	1.49(4)
C(115)-H(115)	0.9300
C(115)-C(116)	1.49(3)
C(116)-H(116)	0.9300
C(117)-C(118)	1.47(3)
C(117)-C(119)	1.55(4)
C(117)-C(120)	1.51(4)
C(118)-H(11D)	0.9600
C(118)-H(11E)	0.9600
C(118)-H(11F)	0.9600
C(119)-H(11G)	0.9600
C(119)-H(11H)	0.9600
C(119)-H(11I)	0.9600
C(120)-H(12A)	0.9600
C(120)-H(12B)	0.9600
C(120)-H(12C)	0.9600
C(121)-C(122)	1.33(2)
C(121)-C(126)	1.40(2)
C(122)-H(122)	0.9300
C(122)-C(123)	1.38(2)
C(123)-H(123)	0.9300
C(123)-C(124)	1.41(2)
C(124)-C(125)	1.35(2)
C(124)-C(127)	1.57(2)
C(125)-H(125)	0.9300
C(125)-C(126)	1.39(2)
C(126)-H(126)	0.9300
C(127)-C(128)	1.60(3)
C(127)-C(129)	1.51(3)
C(127)-C(130)	1.50(2)
C(128)-H(12D)	0.9600
C(128)-H(12E)	0.9600

C(128)-H(12F)	0.9600
C(129)-H(12G)	0.9600
C(129)-H(12H)	0.9600
C(129)-H(12I)	0.9600
C(130)-H(13A)	0.9600
C(130)-H(13B)	0.9600
C(130)-H(13C)	0.9600
C(131)-C(132)	1.40(2)
C(131)-C(136)	1.36(2)
C(132)-H(132)	0.9300
C(132)-C(133)	1.41(2)
C(133)-H(133)	0.9300
C(133)-C(134)	1.36(2)
C(134)-C(135)	1.39(2)
C(134)-C(137)	1.56(2)
C(135)-H(135)	0.9300
C(135)-C(136)	1.39(2)
C(136)-H(136)	0.9300
C(137)-C(138)	1.51(2)
C(137)-C(139)	1.48(3)
C(137)-C(140)	1.49(3)
C(138)-H(13D)	0.9600
C(138)-H(13E)	0.9600
C(138)-H(13F)	0.9600
C(139)-H(13G)	0.9600
C(139)-H(13H)	0.9600
C(139)-H(13I)	0.9600
C(140)-H(14A)	0.9600
C(140)-H(14B)	0.9600
C(140)-H(14C)	0.9600
C(141)-C(142)	1.42(2)
C(141)-C(146)	1.36(2)
C(142)-H(142)	0.9300
C(142)-C(143)	1.45(2)
C(143)-H(143)	0.9300
C(143)-C(144)	1.35(3)
C(144)-C(145)	1.35(2)
C(144)-C(147)	1.60(3)

C(145)-H(145)	0.9300
C(145)-C(146)	1.38(3)
C(146)-H(146)	0.9300
C(147)-C(148)	1.50(3)
C(147)-C(149)	1.54(3)
C(147)-C(150)	1.53(3)
C(148)-H(14D)	0.9600
C(148)-H(14E)	0.9600
C(148)-H(14F)	0.9600
C(149)-H(14G)	0.9600
C(149)-H(14H)	0.9600
C(149)-H(14I)	0.9600
C(150)-H(15A)	0.9600
C(150)-H(15B)	0.9600
C(150)-H(15C)	0.9600
C(151)-C(152)	1.377(19)
C(151)-C(156)	1.39(2)
C(152)-H(152)	0.9300
C(152)-C(153)	1.37(2)
C(153)-H(153)	0.9300
C(153)-C(154)	1.41(2)
C(154)-C(155)	1.42(2)
C(154)-C(157)	1.51(3)
C(155)-H(155)	0.9300
C(155)-C(156)	1.40(2)
C(156)-H(156)	0.9300
C(157)-C(158)	1.55(3)
C(157)-C(159)	1.46(3)
C(157)-C(160)	1.50(3)
C(158)-H(15D)	0.9600
C(158)-H(15E)	0.9600
C(158)-H(15F)	0.9600
C(159)-H(15G)	0.9600
C(159)-H(15H)	0.9600
C(159)-H(15I)	0.9600
C(160)-H(16A)	0.9600
C(160)-H(16B)	0.9600
C(160)-H(16C)	0.9600

C(161)-C(162)	1.40(2)
C(161)-C(166)	1.379(19)
C(162)-H(162)	0.9300
C(162)-C(163)	1.41(2)
C(163)-H(163)	0.9300
C(163)-C(164)	1.39(2)
C(164)-C(165)	1.44(2)
C(164)-C(167)	1.54(2)
C(165)-H(165)	0.9300
C(165)-C(166)	1.39(2)
C(166)-H(166)	0.9300
C(167)-C(168)	1.54(3)
C(167)-C(169)	1.57(3)
C(167)-C(170)	1.51(2)
C(168)-H(16D)	0.9600
C(168)-H(16E)	0.9600
C(168)-H(16F)	0.9600
C(169)-H(16G)	0.9600
C(169)-H(16H)	0.9600
C(169)-H(16I)	0.9600
C(170)-H(17A)	0.9600
C(170)-H(17B)	0.9600
C(170)-H(17C)	0.9600
C(171)-C(172)	1.38(2)
C(171)-C(176)	1.39(2)
C(172)-H(172)	0.9300
C(172)-C(173)	1.39(2)
C(173)-H(173)	0.9300
C(173)-C(174)	1.40(2)
C(174)-C(175)	1.36(2)
C(174)-C(177)	1.58(3)
C(175)-H(175)	0.9300
C(175)-C(176)	1.39(2)
C(176)-H(176)	0.9300
C(177)-C(178)	1.41(3)
C(177)-C(179)	1.51(3)
C(177)-C(180)	1.47(3)
C(178)-H(17D)	0.9600

C(178)-H(17E)	0.9600
C(178)-H(17F)	0.9600
C(179)-H(17G)	0.9600
C(179)-H(17H)	0.9600
C(179)-H(17I)	0.9600
C(180)-H(18D)	0.9600
C(180)-H(18E)	0.9600
C(180)-H(18F)	0.9600
C(181)-C(182)	1.35(2)
C(181)-C(186)	1.41(2)
C(182)-H(182)	0.9300
C(182)-C(183)	1.38(2)
C(183)-H(183)	0.9300
C(183)-C(184)	1.36(2)
C(184)-C(185)	1.42(2)
C(184)-C(187)	1.56(2)
C(185)-H(185)	0.9300
C(185)-C(186)	1.35(2)
C(186)-H(186)	0.9300
C(187)-C(188)	1.52(3)
C(187)-C(189)	1.54(3)
C(187)-C(190)	1.57(3)
C(188)-H(18G)	0.9600
C(188)-H(18H)	0.9600
C(188)-H(18I)	0.9600
C(189)-H(18J)	0.9600
C(189)-H(18K)	0.9600
C(189)-H(18L)	0.9600
C(190)-H(19D)	0.9600
C(190)-H(19E)	0.9600
C(190)-H(19F)	0.9600
C(191)-C(192)	1.37(2)
C(191)-C(196)	1.370(19)
C(192)-H(192)	0.9300
C(192)-C(193)	1.40(2)
C(193)-H(193)	0.9300
C(193)-C(194)	1.36(2)
C(194)-C(195)	1.38(3)

C(194)-C(197)	1.54(3)
C(195)-H(195)	0.9300
C(195)-C(196)	1.40(2)
C(196)-H(196)	0.9300
C(197)-C(198)	1.50(3)
C(197)-C(199)	1.47(3)
C(197)-C(200)	1.52(3)
C(198)-H(19G)	0.9600
C(198)-H(19H)	0.9600
C(198)-H(19I)	0.9600
C(199)-H(19J)	0.9600
C(199)-H(19K)	0.9600
C(199)-H(19L)	0.9600
C(200)-H(20D)	0.9600
C(200)-H(20E)	0.9600
C(200)-H(20F)	0.9600
C(201)-C(202)	1.37(2)
C(201)-C(206)	1.36(2)
C(202)-H(202)	0.9300
C(202)-C(203)	1.383(19)
C(203)-H(203)	0.9300
C(203)-C(204)	1.39(2)
C(204)-C(205)	1.38(2)
C(204)-C(207)	1.58(2)
C(205)-H(205)	0.9300
C(205)-C(206)	1.391(19)
C(206)-H(206)	0.9300
C(207)-C(208)	1.51(2)
C(207)-C(209)	1.52(2)
C(207)-C(210)	1.54(2)
C(208)-H(20G)	0.9600
C(208)-H(20H)	0.9600
C(208)-H(20I)	0.9600
C(209)-H(20J)	0.9600
C(209)-H(20K)	0.9600
C(209)-H(20L)	0.9600
C(210)-H(21A)	0.9600
C(210)-H(21B)	0.9600

C(210)-H(21C)	0.9600
C(211)-C(212)	1.38(2)
C(211)-C(216)	1.35(2)
C(212)-H(212)	0.9300
C(212)-C(213)	1.35(2)
C(213)-H(213)	0.9300
C(213)-C(214)	1.39(2)
C(214)-C(215)	1.39(2)
C(214)-C(217)	1.58(3)
C(215)-H(215)	0.9300
C(215)-C(216)	1.41(2)
C(216)-H(216)	0.9300
C(217)-C(218)	1.44(3)
C(217)-C(219)	1.53(3)
C(217)-C(220)	1.51(3)
C(218)-H(21D)	0.9600
C(218)-H(21E)	0.9600
C(218)-H(21F)	0.9600
C(219)-H(21G)	0.9600
C(219)-H(21H)	0.9600
C(219)-H(21I)	0.9600
C(220)-H(22A)	0.9600
C(220)-H(22B)	0.9600
C(220)-H(22C)	0.9600
C(221)-C(222)	1.40(2)
C(221)-C(226)	1.37(2)
C(222)-H(222)	0.9300
C(222)-C(223)	1.38(3)
C(223)-H(223)	0.9300
C(223)-C(224)	1.35(3)
C(224)-C(225)	1.41(3)
C(224)-C(227)	1.532(13)
C(225)-H(225)	0.9300
C(225)-C(226)	1.40(3)
C(226)-H(226)	0.9300
C(227)-C(228)	1.513(13)
C(227)-C(229)	1.529(14)
C(227)-C(260)	1.515(13)

C(228)-H(22D)	0.9600
C(228)-H(22E)	0.9600
C(228)-H(22F)	0.9600
C(229)-H(22G)	0.9600
C(229)-H(22H)	0.9600
C(229)-H(22I)	0.9600
C(230)-C(231)	1.43(2)
C(230)-C(235)	1.402(19)
C(231)-H(231)	0.9300
C(231)-C(232)	1.34(2)
C(232)-H(232)	0.9300
C(232)-C(233)	1.41(2)
C(233)-C(234)	1.45(2)
C(233)-C(236)	1.53(3)
C(234)-H(234)	0.9300
C(234)-C(235)	1.37(2)
C(235)-H(235)	0.9300
C(236)-C(237)	1.59(3)
C(236)-C(238)	1.46(3)
C(236)-C(239)	1.50(3)
C(237)-H(23A)	0.9600
C(237)-H(23B)	0.9600
C(237)-H(23C)	0.9600
C(238)-H(23D)	0.9600
C(238)-H(23E)	0.9600
C(238)-H(23F)	0.9600
C(239)-H(23G)	0.9600
C(239)-H(23H)	0.9600
C(239)-H(23I)	0.9600
C(240)-C(241)	1.39(2)
C(240)-C(245)	1.41(2)
C(241)-H(241)	0.9300
C(241)-C(242)	1.39(2)
C(242)-H(242)	0.9300
C(242)-C(243)	1.37(2)
C(243)-C(244)	1.41(2)
C(243)-C(246)	1.49(3)
C(244)-H(244)	0.9300

C(244)-C(245)	1.34(3)
C(245)-H(245)	0.9300
C(246)-C(247)	1.50(3)
C(246)-C(248)	1.56(3)
C(246)-C(249)	1.45(3)
C(247)-H(24A)	0.9600
C(247)-H(24B)	0.9600
C(247)-H(24C)	0.9600
C(248)-H(24D)	0.9600
C(248)-H(24E)	0.9600
C(248)-H(24F)	0.9600
C(249)-H(24G)	0.9600
C(249)-H(24H)	0.9600
C(249)-H(24I)	0.9600
C(250)-C(251)	1.42(2)
C(250)-C(255)	1.37(2)
C(251)-H(251)	0.9300
C(251)-C(252)	1.353(19)
C(252)-H(252)	0.9300
C(252)-C(253)	1.41(2)
C(253)-C(254)	1.33(2)
C(253)-C(256)	1.56(2)
C(254)-H(254)	0.9300
C(254)-C(255)	1.39(2)
C(255)-H(255)	0.9300
C(256)-C(257)	1.53(3)
C(256)-C(258)	1.39(3)
C(256)-C(259)	1.47(3)
C(257)-H(25A)	0.9600
C(257)-H(25B)	0.9600
C(257)-H(25C)	0.9600
C(258)-H(25D)	0.9600
C(258)-H(25E)	0.9600
C(258)-H(25F)	0.9600
C(259)-H(25G)	0.9600
C(259)-H(25H)	0.9600
C(259)-H(25I)	0.9600
C(260)-H(26A)	0.9600

C(260)-H(26B)	0.9600
C(260)-H(26C)	0.9600
Au(2)-Au(1)-Au(14)	121.70(2)
Au(2)-Au(1)-Au(15)	176.35(2)
Au(2)-Au(1)-Au(19)	118.36(2)
Au(3)-Au(1)-Au(2)	62.34(2)
Au(3)-Au(1)-Au(7)	61.095(19)
Au(3)-Au(1)-Au(14)	59.366(19)
Au(3)-Au(1)-Au(15)	114.22(2)
Au(3)-Au(1)-Au(19)	176.39(2)
Au(3)-Au(1)-Au(20)	61.588(19)
Au(5)-Au(1)-Au(2)	116.81(3)
Au(5)-Au(1)-Au(3)	89.89(2)
Au(5)-Au(1)-Au(7)	58.25(2)
Au(5)-Au(1)-Au(14)	63.251(19)
Au(5)-Au(1)-Au(15)	61.12(2)
Au(5)-Au(1)-Au(16)	119.57(3)
Au(5)-Au(1)-Au(19)	86.69(2)
Au(5)-Au(1)-Au(20)	124.20(3)
Au(6)-Au(1)-Au(2)	80.28(3)
Au(6)-Au(1)-Au(3)	118.31(3)
Au(6)-Au(1)-Au(5)	64.45(2)
Au(6)-Au(1)-Au(7)	57.66(2)
Au(6)-Au(1)-Au(14)	127.63(2)
Au(6)-Au(1)-Au(15)	100.92(2)
Au(6)-Au(1)-Au(16)	119.55(2)
Au(6)-Au(1)-Au(17)	112.68(3)
Au(6)-Au(1)-Au(19)	59.04(2)
Au(6)-Au(1)-Au(20)	170.85(3)
Au(7)-Au(1)-Au(2)	58.64(2)
Au(7)-Au(1)-Au(14)	91.83(2)
Au(7)-Au(1)-Au(15)	119.05(2)
Au(7)-Au(1)-Au(19)	115.91(2)
Au(7)-Au(1)-Au(20)	122.61(2)
Au(14)-Au(1)-Au(15)	54.853(18)
Au(14)-Au(1)-Au(19)	119.76(3)
Au(15)-Au(1)-Au(19)	64.988(19)

Au(16)-Au(1)-Au(2)	123.34(2)
Au(16)-Au(1)-Au(3)	121.87(2)
Au(16)-Au(1)-Au(7)	176.79(2)
Au(16)-Au(1)-Au(14)	88.98(2)
Au(16)-Au(1)-Au(15)	59.11(2)
Au(16)-Au(1)-Au(19)	61.076(19)
Au(16)-Au(1)-Au(20)	60.421(19)
Au(17)-Au(1)-Au(2)	62.95(2)
Au(17)-Au(1)-Au(3)	92.58(2)
Au(17)-Au(1)-Au(5)	176.93(3)
Au(17)-Au(1)-Au(7)	121.58(3)
Au(17)-Au(1)-Au(14)	119.65(2)
Au(17)-Au(1)-Au(15)	119.30(3)
Au(17)-Au(1)-Au(16)	60.45(2)
Au(17)-Au(1)-Au(19)	90.81(2)
Au(17)-Au(1)-Au(20)	58.723(19)
Au(20)-Au(1)-Au(2)	92.26(2)
Au(20)-Au(1)-Au(14)	60.950(18)
Au(20)-Au(1)-Au(15)	86.85(2)
Au(20)-Au(1)-Au(19)	121.47(2)
Au(1)-Au(2)-Au(3)	56.866(19)
Au(1)-Au(2)-Au(17)	54.581(19)
Au(1)-Au(2)-Au(18)	71.86(2)
Au(3)-Au(2)-Au(18)	128.15(3)
Au(7)-Au(2)-Au(1)	59.77(2)
Au(7)-Au(2)-Au(3)	59.571(19)
Au(7)-Au(2)-Au(17)	114.35(3)
Au(7)-Au(2)-Au(18)	102.28(2)
Au(10)-Au(2)-Au(1)	111.68(3)
Au(10)-Au(2)-Au(3)	55.80(2)
Au(10)-Au(2)-Au(7)	77.85(2)
Au(10)-Au(2)-Au(11)	63.05(2)
Au(10)-Au(2)-Au(17)	126.26(3)
Au(10)-Au(2)-Au(18)	175.56(3)
Au(11)-Au(2)-Au(1)	89.48(3)
Au(11)-Au(2)-Au(3)	56.809(19)
Au(11)-Au(2)-Au(7)	116.11(3)
Au(11)-Au(2)-Au(17)	65.10(2)

Au(11)-Au(2)-Au(18)	120.31(3)
Au(17)-Au(2)-Au(3)	84.53(2)
Au(17)-Au(2)-Au(18)	57.83(2)
S(3)-Au(2)-Au(1)	117.43(10)
S(3)-Au(2)-Au(3)	172.47(9)
S(3)-Au(2)-Au(7)	113.81(9)
S(3)-Au(2)-Au(10)	128.32(11)
S(3)-Au(2)-Au(11)	130.05(9)
S(3)-Au(2)-Au(17)	95.81(10)
S(3)-Au(2)-Au(18)	47.49(10)
Au(1)-Au(3)-Au(2)	60.79(2)
Au(1)-Au(3)-Au(7)	60.597(19)
Au(1)-Au(3)-Au(9)	175.54(3)
Au(1)-Au(3)-Au(12)	117.76(2)
Au(1)-Au(3)-Au(14)	63.400(19)
Au(1)-Au(3)-Au(20)	60.517(19)
Au(2)-Au(3)-Au(9)	115.08(2)
Au(2)-Au(3)-Au(12)	117.25(2)
Au(4)-Au(3)-Au(1)	93.14(2)
Au(4)-Au(3)-Au(2)	122.20(2)
Au(4)-Au(3)-Au(7)	64.23(2)
Au(4)-Au(3)-Au(9)	87.85(2)
Au(4)-Au(3)-Au(11)	172.93(3)
Au(4)-Au(3)-Au(12)	120.51(2)
Au(4)-Au(3)-Au(14)	59.75(2)
Au(4)-Au(3)-Au(20)	121.69(3)
Au(4)-Au(3)-Au(32)	59.710(19)
Au(7)-Au(3)-Au(2)	57.998(18)
Au(7)-Au(3)-Au(9)	116.19(2)
Au(7)-Au(3)-Au(12)	175.25(3)
Au(7)-Au(3)-Au(20)	121.05(2)
Au(10)-Au(3)-Au(1)	116.63(3)
Au(10)-Au(3)-Au(2)	56.92(2)
Au(10)-Au(3)-Au(4)	109.76(3)
Au(10)-Au(3)-Au(7)	77.57(2)
Au(10)-Au(3)-Au(9)	59.00(2)
Au(10)-Au(3)-Au(11)	63.52(2)
Au(10)-Au(3)-Au(12)	100.00(2)

Au(10)-Au(3)-Au(14)	169.00(3)
Au(10)-Au(3)-Au(20)	128.46(3)
Au(10)-Au(3)-Au(32)	119.11(3)
Au(11)-Au(3)-Au(1)	92.06(2)
Au(11)-Au(3)-Au(2)	56.874(19)
Au(11)-Au(3)-Au(7)	114.61(3)
Au(11)-Au(3)-Au(9)	86.60(2)
Au(11)-Au(3)-Au(12)	60.67(2)
Au(11)-Au(3)-Au(14)	127.09(3)
Au(11)-Au(3)-Au(20)	65.17(2)
Au(11)-Au(3)-Au(32)	120.69(2)
Au(12)-Au(3)-Au(9)	65.165(19)
Au(14)-Au(3)-Au(2)	124.19(2)
Au(14)-Au(3)-Au(7)	93.89(2)
Au(14)-Au(3)-Au(9)	120.67(2)
Au(14)-Au(3)-Au(12)	89.00(2)
Au(14)-Au(3)-Au(20)	61.94(2)
Au(20)-Au(3)-Au(2)	90.78(2)
Au(20)-Au(3)-Au(9)	122.43(2)
Au(20)-Au(3)-Au(12)	57.282(18)
Au(32)-Au(3)-Au(1)	123.40(3)
Au(32)-Au(3)-Au(2)	175.74(3)
Au(32)-Au(3)-Au(7)	123.90(2)
Au(32)-Au(3)-Au(9)	60.75(2)
Au(32)-Au(3)-Au(12)	60.842(19)
Au(32)-Au(3)-Au(14)	60.02(2)
Au(32)-Au(3)-Au(20)	91.05(2)
Au(3)-Au(4)-Au(5)	86.43(2)
Au(3)-Au(4)-Au(7)	60.814(19)
Au(3)-Au(4)-Au(8)	77.74(2)
Au(3)-Au(4)-Au(14)	63.06(2)
Au(3)-Au(4)-Au(33)	138.50(3)
Au(5)-Au(4)-Au(7)	54.379(19)
Au(5)-Au(4)-Au(33)	97.81(2)
Au(7)-Au(4)-Au(33)	149.11(2)
Au(8)-Au(4)-Au(5)	113.58(3)
Au(8)-Au(4)-Au(7)	61.81(2)
Au(8)-Au(4)-Au(33)	134.68(3)

Au(14)-Au(4)-Au(5)	62.592(19)
Au(14)-Au(4)-Au(7)	93.78(2)
Au(14)-Au(4)-Au(8)	140.60(3)
Au(14)-Au(4)-Au(33)	82.32(2)
Au(32)-Au(4)-Au(3)	60.52(2)
Au(32)-Au(4)-Au(5)	123.20(3)
Au(32)-Au(4)-Au(7)	121.29(2)
Au(32)-Au(4)-Au(8)	103.34(3)
Au(32)-Au(4)-Au(14)	61.42(2)
Au(32)-Au(4)-Au(33)	83.69(2)
S(11)-Au(4)-Au(3)	172.42(10)
S(11)-Au(4)-Au(5)	92.89(9)
S(11)-Au(4)-Au(7)	112.84(9)
S(11)-Au(4)-Au(8)	95.67(10)
S(11)-Au(4)-Au(14)	123.14(10)
S(11)-Au(4)-Au(32)	125.44(9)
S(11)-Au(4)-Au(33)	49.08(10)
Au(1)-Au(5)-Au(4)	90.18(2)
Au(1)-Au(5)-Au(6)	57.63(2)
Au(1)-Au(5)-Au(7)	64.25(2)
Au(1)-Au(5)-Au(14)	63.183(19)
Au(1)-Au(5)-Au(15)	66.17(2)
Au(4)-Au(5)-Au(14)	55.825(18)
Au(4)-Au(5)-Au(15)	111.56(2)
Au(6)-Au(5)-Au(4)	120.02(3)
Au(6)-Au(5)-Au(14)	120.75(2)
Au(6)-Au(5)-Au(15)	99.94(2)
Au(7)-Au(5)-Au(4)	63.19(2)
Au(7)-Au(5)-Au(6)	57.45(2)
Au(7)-Au(5)-Au(14)	95.08(2)
Au(7)-Au(5)-Au(15)	130.02(2)
Au(15)-Au(5)-Au(14)	56.197(18)
S(13)-Au(5)-Au(1)	169.33(11)
S(13)-Au(5)-Au(4)	93.82(9)
S(13)-Au(5)-Au(6)	127.46(9)
S(13)-Au(5)-Au(7)	126.30(11)
S(13)-Au(5)-Au(14)	111.34(10)
S(13)-Au(5)-Au(15)	103.16(11)

Au(1)-Au(6)-Au(5)	57.91(2)
Au(1)-Au(6)-Au(7)	64.75(2)
Au(1)-Au(6)-Au(18)	74.08(2)
Au(1)-Au(6)-Au(19)	68.81(2)
Au(1)-Au(6)-Au(42)	124.78(3)
Au(5)-Au(6)-Au(18)	131.94(2)
Au(5)-Au(6)-Au(19)	88.60(3)
Au(5)-Au(6)-Au(42)	133.17(3)
Au(7)-Au(6)-Au(5)	58.42(2)
Au(7)-Au(6)-Au(18)	104.42(2)
Au(7)-Au(6)-Au(19)	132.44(3)
Au(7)-Au(6)-Au(42)	80.10(2)
Au(18)-Au(6)-Au(42)	75.42(2)
Au(19)-Au(6)-Au(18)	71.27(2)
Au(19)-Au(6)-Au(42)	138.02(3)
S(26)-Au(6)-Au(1)	157.63(10)
S(26)-Au(6)-Au(5)	144.46(10)
S(26)-Au(6)-Au(7)	122.00(11)
S(26)-Au(6)-Au(18)	83.56(10)
S(26)-Au(6)-Au(19)	104.88(11)
S(26)-Au(6)-Au(42)	45.81(11)
Au(1)-Au(7)-Au(3)	58.308(19)
Au(1)-Au(7)-Au(4)	86.36(2)
Au(1)-Au(7)-Au(8)	130.90(2)
Au(2)-Au(7)-Au(1)	61.60(2)
Au(2)-Au(7)-Au(3)	62.43(2)
Au(2)-Au(7)-Au(4)	117.35(2)
Au(2)-Au(7)-Au(8)	103.62(2)
Au(3)-Au(7)-Au(4)	54.951(18)
Au(3)-Au(7)-Au(8)	73.18(2)
Au(4)-Au(7)-Au(8)	57.85(2)
Au(5)-Au(7)-Au(1)	57.49(2)
Au(5)-Au(7)-Au(2)	119.01(3)
Au(5)-Au(7)-Au(3)	87.76(3)
Au(5)-Au(7)-Au(4)	62.43(2)
Au(5)-Au(7)-Au(8)	117.45(3)
Au(6)-Au(7)-Au(1)	57.59(2)
Au(6)-Au(7)-Au(2)	81.83(2)

Au(6)-Au(7)-Au(3)	115.48(3)
Au(6)-Au(7)-Au(4)	125.85(3)
Au(6)-Au(7)-Au(5)	64.12(2)
Au(6)-Au(7)-Au(8)	171.33(3)
S(1)-Au(7)-Au(1)	171.86(9)
S(1)-Au(7)-Au(2)	110.27(9)
S(1)-Au(7)-Au(3)	119.35(8)
S(1)-Au(7)-Au(4)	98.40(9)
S(1)-Au(7)-Au(5)	130.61(9)
S(1)-Au(7)-Au(6)	122.97(9)
S(1)-Au(7)-Au(8)	48.98(8)
Au(4)-Au(8)-Au(7)	60.35(2)
Au(4)-Au(8)-Au(10)	95.95(2)
Au(10)-Au(8)-Au(7)	70.50(2)
S(1)-Au(8)-Au(4)	102.11(10)
S(1)-Au(8)-Au(7)	50.80(10)
S(1)-Au(8)-Au(10)	92.21(9)
S(2)-Au(8)-Au(4)	85.72(11)
S(2)-Au(8)-Au(7)	138.36(11)
S(2)-Au(8)-Au(10)	91.70(10)
S(2)-Au(8)-S(1)	170.83(15)
Au(3)-Au(9)-Au(12)	54.535(17)
Au(10)-Au(9)-Au(3)	51.418(19)
Au(10)-Au(9)-Au(12)	87.55(2)
Au(10)-Au(9)-Au(32)	102.98(2)
Au(32)-Au(9)-Au(3)	52.031(17)
Au(32)-Au(9)-Au(12)	54.153(18)
S(2)-Au(9)-Au(3)	97.06(10)
S(2)-Au(9)-Au(10)	94.22(10)
S(2)-Au(9)-Au(12)	140.57(11)
S(2)-Au(9)-Au(32)	87.33(11)
S(2)-Au(9)-S(23)	173.77(14)
S(23)-Au(9)-Au(3)	88.37(9)
S(23)-Au(9)-Au(10)	86.78(9)
S(23)-Au(9)-Au(12)	45.57(10)
S(23)-Au(9)-Au(32)	98.45(11)
Au(2)-Au(10)-Au(8)	107.59(2)
Au(2)-Au(10)-Au(9)	135.55(3)

Au(2)-Au(10)-Au(11)	58.79(2)
Au(2)-Au(10)-Au(41)	74.97(2)
Au(3)-Au(10)-Au(2)	67.29(2)
Au(3)-Au(10)-Au(8)	76.55(2)
Au(3)-Au(10)-Au(9)	69.58(2)
Au(3)-Au(10)-Au(11)	59.12(2)
Au(3)-Au(10)-Au(41)	128.53(3)
Au(8)-Au(10)-Au(41)	83.20(2)
Au(9)-Au(10)-Au(8)	71.61(2)
Au(9)-Au(10)-Au(41)	144.77(3)
Au(11)-Au(10)-Au(8)	135.62(3)
Au(11)-Au(10)-Au(9)	89.97(3)
Au(11)-Au(10)-Au(41)	125.07(3)
S(24)-Au(10)-Au(2)	117.50(10)
S(24)-Au(10)-Au(3)	163.99(10)
S(24)-Au(10)-Au(8)	87.50(9)
S(24)-Au(10)-Au(9)	106.93(10)
S(24)-Au(10)-Au(11)	136.87(9)
S(24)-Au(10)-Au(41)	46.30(9)
Au(2)-Au(11)-Au(10)	58.16(2)
Au(2)-Au(11)-Au(12)	130.38(3)
Au(2)-Au(11)-Au(17)	61.00(2)
Au(2)-Au(11)-Au(20)	93.34(2)
Au(2)-Au(11)-Au(26)	139.88(2)
Au(3)-Au(11)-Au(2)	66.32(2)
Au(3)-Au(11)-Au(10)	57.36(2)
Au(3)-Au(11)-Au(12)	64.45(2)
Au(3)-Au(11)-Au(17)	87.02(2)
Au(3)-Au(11)-Au(20)	60.62(2)
Au(3)-Au(11)-Au(26)	131.81(3)
Au(10)-Au(11)-Au(12)	98.57(2)
Au(10)-Au(11)-Au(17)	117.59(2)
Au(10)-Au(11)-Au(20)	117.79(2)
Au(10)-Au(11)-Au(26)	159.65(2)
Au(12)-Au(11)-Au(17)	110.21(3)
Au(12)-Au(11)-Au(20)	57.022(19)
Au(12)-Au(11)-Au(26)	75.52(2)
Au(17)-Au(11)-Au(26)	82.52(2)

Au(20)-Au(11)-Au(17)	53.502(18)
Au(20)-Au(11)-Au(26)	75.76(2)
S(6)-Au(11)-Au(2)	120.34(10)
S(6)-Au(11)-Au(3)	173.34(10)
S(6)-Au(11)-Au(10)	125.11(11)
S(6)-Au(11)-Au(12)	108.94(10)
S(6)-Au(11)-Au(17)	96.33(9)
S(6)-Au(11)-Au(20)	117.06(11)
S(6)-Au(11)-Au(26)	43.52(11)
Au(3)-Au(12)-Au(9)	60.300(18)
Au(11)-Au(12)-Au(3)	54.882(18)
Au(11)-Au(12)-Au(9)	81.17(2)
Au(11)-Au(12)-Au(28)	165.26(3)
Au(11)-Au(12)-Au(32)	109.31(2)
Au(13)-Au(12)-Au(3)	90.24(2)
Au(13)-Au(12)-Au(9)	121.23(2)
Au(13)-Au(12)-Au(11)	123.98(2)
Au(13)-Au(12)-Au(20)	60.423(19)
Au(13)-Au(12)-Au(28)	67.85(2)
Au(13)-Au(12)-Au(32)	63.99(2)
Au(20)-Au(12)-Au(3)	59.978(18)
Au(20)-Au(12)-Au(9)	120.26(2)
Au(20)-Au(12)-Au(11)	64.05(2)
Au(20)-Au(12)-Au(28)	128.25(3)
Au(20)-Au(12)-Au(32)	89.19(2)
Au(25)-Au(12)-Au(3)	119.62(2)
Au(25)-Au(12)-Au(9)	178.95(3)
Au(25)-Au(12)-Au(11)	97.93(2)
Au(25)-Au(12)-Au(13)	59.73(2)
Au(25)-Au(12)-Au(20)	59.64(2)
Au(25)-Au(12)-Au(28)	95.98(3)
Au(25)-Au(12)-Au(32)	123.52(3)
Au(28)-Au(12)-Au(3)	121.02(2)
Au(28)-Au(12)-Au(9)	84.86(2)
Au(32)-Au(12)-Au(3)	55.090(18)
Au(32)-Au(12)-Au(9)	57.396(19)
Au(32)-Au(12)-Au(28)	66.219(19)
S(23)-Au(12)-Au(3)	91.72(9)

S(23)-Au(12)-Au(9)	43.59(9)
S(23)-Au(12)-Au(11)	74.02(9)
S(23)-Au(12)-Au(13)	158.02(9)
S(23)-Au(12)-Au(20)	137.68(9)
S(23)-Au(12)-Au(25)	135.67(9)
S(23)-Au(12)-Au(28)	92.57(9)
S(23)-Au(12)-Au(32)	99.70(9)
Au(12)-Au(13)-Au(14)	91.97(2)
Au(12)-Au(13)-Au(20)	61.13(2)
Au(12)-Au(13)-Au(21)	123.91(2)
Au(12)-Au(13)-Au(24)	122.03(2)
Au(12)-Au(13)-Au(28)	58.988(19)
Au(12)-Au(13)-Au(30)	175.00(2)
Au(12)-Au(13)-Au(32)	60.43(2)
Au(14)-Au(13)-Au(28)	118.17(2)
Au(14)-Au(13)-Au(32)	56.127(17)
Au(20)-Au(13)-Au(14)	62.082(18)
Au(20)-Au(13)-Au(21)	62.87(2)
Au(20)-Au(13)-Au(24)	92.54(2)
Au(20)-Au(13)-Au(28)	120.09(3)
Au(20)-Au(13)-Au(30)	123.86(2)
Au(20)-Au(13)-Au(32)	87.64(2)
Au(21)-Au(13)-Au(14)	58.585(18)
Au(21)-Au(13)-Au(24)	63.012(19)
Au(21)-Au(13)-Au(28)	174.74(2)
Au(21)-Au(13)-Au(30)	61.10(2)
Au(21)-Au(13)-Au(32)	114.70(2)
Au(24)-Au(13)-Au(14)	121.59(2)
Au(24)-Au(13)-Au(28)	120.00(2)
Au(24)-Au(13)-Au(32)	177.19(2)
Au(25)-Au(13)-Au(12)	59.64(2)
Au(25)-Au(13)-Au(14)	121.93(2)
Au(25)-Au(13)-Au(20)	59.851(19)
Au(25)-Au(13)-Au(21)	94.59(2)
Au(25)-Au(13)-Au(24)	62.48(2)
Au(25)-Au(13)-Au(28)	90.67(2)
Au(25)-Au(13)-Au(30)	121.66(3)
Au(25)-Au(13)-Au(31)	174.21(3)

Au(25)-Au(13)-Au(32)	119.88(3)
Au(29)-Au(13)-Au(12)	116.79(3)
Au(29)-Au(13)-Au(14)	126.48(2)
Au(29)-Au(13)-Au(20)	171.35(3)
Au(29)-Au(13)-Au(21)	119.04(3)
Au(29)-Au(13)-Au(24)	81.62(2)
Au(29)-Au(13)-Au(25)	111.59(2)
Au(29)-Au(13)-Au(28)	58.77(2)
Au(29)-Au(13)-Au(30)	58.23(2)
Au(29)-Au(13)-Au(31)	62.85(2)
Au(29)-Au(13)-Au(32)	98.51(2)
Au(30)-Au(13)-Au(14)	91.05(2)
Au(30)-Au(13)-Au(24)	59.23(2)
Au(30)-Au(13)-Au(28)	116.03(2)
Au(30)-Au(13)-Au(32)	118.46(2)
Au(31)-Au(13)-Au(12)	120.54(3)
Au(31)-Au(13)-Au(14)	63.67(2)
Au(31)-Au(13)-Au(20)	125.74(2)
Au(31)-Au(13)-Au(21)	89.82(2)
Au(31)-Au(13)-Au(24)	116.70(3)
Au(31)-Au(13)-Au(28)	84.95(2)
Au(31)-Au(13)-Au(30)	57.60(2)
Au(31)-Au(13)-Au(32)	61.13(2)
Au(32)-Au(13)-Au(28)	62.140(18)
Au(1)-Au(14)-Au(5)	53.566(17)
Au(1)-Au(14)-Au(31)	168.98(2)
Au(3)-Au(14)-Au(1)	57.234(19)
Au(3)-Au(14)-Au(5)	83.04(2)
Au(3)-Au(14)-Au(13)	88.73(2)
Au(3)-Au(14)-Au(20)	59.729(19)
Au(3)-Au(14)-Au(31)	117.16(2)
Au(4)-Au(14)-Au(1)	87.90(2)
Au(4)-Au(14)-Au(3)	57.19(2)
Au(4)-Au(14)-Au(5)	61.583(19)
Au(4)-Au(14)-Au(13)	120.59(2)
Au(4)-Au(14)-Au(15)	122.47(2)
Au(4)-Au(14)-Au(20)	116.92(3)
Au(4)-Au(14)-Au(21)	177.64(3)

Au(4)-Au(14)-Au(31)	96.51(2)
Au(4)-Au(14)-Au(32)	57.942(19)
Au(4)-Au(14)-Au(35)	124.94(2)
Au(5)-Au(14)-Au(31)	137.24(2)
Au(13)-Au(14)-Au(1)	114.43(2)
Au(13)-Au(14)-Au(5)	167.92(2)
Au(13)-Au(14)-Au(20)	56.572(18)
Au(13)-Au(14)-Au(31)	54.704(17)
Au(15)-Au(14)-Au(1)	64.56(2)
Au(15)-Au(14)-Au(3)	121.79(2)
Au(15)-Au(14)-Au(5)	61.45(2)
Au(15)-Au(14)-Au(13)	116.77(2)
Au(15)-Au(14)-Au(20)	90.68(2)
Au(15)-Au(14)-Au(21)	58.498(19)
Au(15)-Au(14)-Au(31)	120.24(3)
Au(15)-Au(14)-Au(32)	178.94(3)
Au(20)-Au(14)-Au(1)	57.864(17)
Au(20)-Au(14)-Au(5)	111.43(2)
Au(20)-Au(14)-Au(31)	111.27(2)
Au(21)-Au(14)-Au(1)	90.77(2)
Au(21)-Au(14)-Au(3)	120.45(2)
Au(21)-Au(14)-Au(5)	118.99(2)
Au(21)-Au(14)-Au(13)	58.309(18)
Au(21)-Au(14)-Au(20)	60.726(19)
Au(21)-Au(14)-Au(31)	84.46(2)
Au(32)-Au(14)-Au(1)	114.66(3)
Au(32)-Au(14)-Au(3)	57.447(19)
Au(32)-Au(14)-Au(5)	118.79(3)
Au(32)-Au(14)-Au(13)	62.750(19)
Au(32)-Au(14)-Au(20)	88.28(2)
Au(32)-Au(14)-Au(21)	121.05(2)
Au(32)-Au(14)-Au(31)	60.38(2)
Au(35)-Au(14)-Au(1)	122.62(2)
Au(35)-Au(14)-Au(3)	177.75(3)
Au(35)-Au(14)-Au(5)	98.63(2)
Au(35)-Au(14)-Au(13)	89.39(2)
Au(35)-Au(14)-Au(15)	58.15(2)
Au(35)-Au(14)-Au(20)	118.13(2)

Au(35)-Au(14)-Au(21)	57.41(2)
Au(35)-Au(14)-Au(31)	62.55(2)
Au(35)-Au(14)-Au(32)	122.58(2)
Au(5)-Au(15)-Au(1)	52.705(19)
Au(5)-Au(15)-Au(37)	169.71(2)
Au(14)-Au(15)-Au(1)	60.588(19)
Au(14)-Au(15)-Au(5)	62.349(19)
Au(14)-Au(15)-Au(16)	89.57(2)
Au(14)-Au(15)-Au(37)	127.03(2)
Au(16)-Au(15)-Au(1)	54.215(19)
Au(16)-Au(15)-Au(5)	106.40(3)
Au(16)-Au(15)-Au(37)	71.44(2)
Au(21)-Au(15)-Au(1)	90.00(2)
Au(21)-Au(15)-Au(5)	122.58(2)
Au(21)-Au(15)-Au(14)	61.266(19)
Au(21)-Au(15)-Au(16)	63.64(2)
Au(21)-Au(15)-Au(37)	66.03(2)
Au(35)-Au(15)-Au(1)	121.29(2)
Au(35)-Au(15)-Au(5)	100.97(2)
Au(35)-Au(15)-Au(14)	60.79(2)
Au(35)-Au(15)-Au(16)	122.71(2)
Au(35)-Au(15)-Au(21)	59.172(19)
Au(35)-Au(15)-Au(37)	88.35(3)
Au(37)-Au(15)-Au(1)	125.60(2)
S(5)-Au(15)-Au(1)	87.47(11)
S(5)-Au(15)-Au(5)	70.57(10)
S(5)-Au(15)-Au(14)	132.65(10)
S(5)-Au(15)-Au(16)	99.18(9)
S(5)-Au(15)-Au(21)	159.88(9)
S(5)-Au(15)-Au(35)	137.52(10)
S(5)-Au(15)-Au(37)	99.61(10)
Au(1)-Au(16)-Au(15)	66.67(2)
Au(1)-Au(16)-Au(19)	66.57(2)
Au(1)-Au(16)-Au(20)	62.434(19)
Au(1)-Au(16)-Au(21)	92.84(2)
Au(1)-Au(16)-Au(22)	125.55(2)
Au(15)-Au(16)-Au(19)	69.32(2)
Au(15)-Au(16)-Au(21)	55.894(18)

Au(15)-Au(16)-Au(22)	109.64(3)
Au(17)-Au(16)-Au(1)	58.97(2)
Au(17)-Au(16)-Au(15)	125.34(3)
Au(17)-Au(16)-Au(19)	93.21(3)
Au(17)-Au(16)-Au(20)	58.98(2)
Au(17)-Au(16)-Au(21)	119.70(2)
Au(17)-Au(16)-Au(22)	97.47(2)
Au(20)-Au(16)-Au(15)	91.91(2)
Au(20)-Au(16)-Au(19)	128.97(3)
Au(20)-Au(16)-Au(21)	60.729(19)
Au(20)-Au(16)-Au(22)	63.487(19)
Au(21)-Au(16)-Au(19)	125.11(2)
Au(22)-Au(16)-Au(19)	167.04(3)
Au(22)-Au(16)-Au(21)	54.629(18)
S(16)-Au(16)-Au(1)	155.81(8)
S(16)-Au(16)-Au(15)	95.28(9)
S(16)-Au(16)-Au(17)	138.21(10)
S(16)-Au(16)-Au(19)	92.57(8)
S(16)-Au(16)-Au(20)	137.38(9)
S(16)-Au(16)-Au(21)	89.47(10)
S(16)-Au(16)-Au(22)	74.57(8)
Au(1)-Au(17)-Au(2)	62.47(2)
Au(1)-Au(17)-Au(11)	87.45(2)
Au(1)-Au(17)-Au(16)	60.59(2)
Au(1)-Au(17)-Au(18)	77.88(2)
Au(1)-Au(17)-Au(20)	64.21(2)
Au(1)-Au(17)-Au(40)	138.61(3)
Au(2)-Au(17)-Au(11)	53.908(18)
Au(2)-Au(17)-Au(40)	149.98(3)
Au(16)-Au(17)-Au(2)	123.00(2)
Au(16)-Au(17)-Au(11)	124.48(2)
Au(16)-Au(17)-Au(18)	101.87(2)
Au(16)-Au(17)-Au(20)	62.34(2)
Au(16)-Au(17)-Au(40)	82.59(3)
Au(18)-Au(17)-Au(2)	63.42(2)
Au(18)-Au(17)-Au(11)	114.99(3)
Au(18)-Au(17)-Au(40)	131.59(2)
Au(20)-Au(17)-Au(2)	95.04(2)

Au(20)-Au(17)-Au(11)	63.03(2)
Au(20)-Au(17)-Au(18)	142.01(3)
Au(20)-Au(17)-Au(40)	82.97(2)
Au(40)-Au(17)-Au(11)	99.97(2)
S(22)-Au(17)-Au(1)	172.22(11)
S(22)-Au(17)-Au(2)	110.90(11)
S(22)-Au(17)-Au(11)	91.53(9)
S(22)-Au(17)-Au(16)	125.67(11)
S(22)-Au(17)-Au(18)	95.62(10)
S(22)-Au(17)-Au(20)	121.95(10)
S(22)-Au(17)-Au(40)	49.15(11)
Au(2)-Au(18)-Au(6)	71.34(2)
Au(17)-Au(18)-Au(2)	58.758(19)
Au(17)-Au(18)-Au(6)	95.34(2)
S(3)-Au(18)-Au(2)	49.69(9)
S(3)-Au(18)-Au(6)	94.78(9)
S(3)-Au(18)-Au(17)	98.41(9)
S(4)-Au(18)-Au(2)	138.62(10)
S(4)-Au(18)-Au(6)	88.60(10)
S(4)-Au(18)-Au(17)	88.87(10)
S(4)-Au(18)-S(3)	171.63(14)
Au(6)-Au(19)-Au(1)	52.149(18)
Au(6)-Au(19)-Au(16)	104.08(2)
Au(16)-Au(19)-Au(1)	52.352(17)
S(4)-Au(19)-Au(1)	96.38(10)
S(4)-Au(19)-Au(6)	93.92(11)
S(4)-Au(19)-Au(16)	87.09(10)
S(5)-Au(19)-Au(1)	87.13(9)
S(5)-Au(19)-Au(6)	85.81(11)
S(5)-Au(19)-Au(16)	97.48(9)
S(5)-Au(19)-S(4)	175.35(13)
Au(1)-Au(20)-Au(3)	57.895(18)
Au(1)-Au(20)-Au(11)	84.27(2)
Au(1)-Au(20)-Au(14)	61.186(18)
Au(1)-Au(20)-Au(21)	90.64(2)
Au(1)-Au(20)-Au(22)	116.61(2)
Au(3)-Au(20)-Au(11)	54.207(19)
Au(3)-Au(20)-Au(14)	58.332(19)

Au(3)-Au(20)-Au(21)	115.48(2)
Au(3)-Au(20)-Au(22)	169.22(2)
Au(12)-Au(20)-Au(1)	120.60(3)
Au(12)-Au(20)-Au(3)	62.741(19)
Au(12)-Au(20)-Au(11)	58.929(19)
Au(12)-Au(20)-Au(14)	90.05(2)
Au(12)-Au(20)-Au(21)	117.08(2)
Au(12)-Au(20)-Au(22)	122.24(2)
Au(13)-Au(20)-Au(1)	122.52(2)
Au(13)-Au(20)-Au(3)	90.75(2)
Au(13)-Au(20)-Au(11)	116.95(2)
Au(13)-Au(20)-Au(12)	58.45(2)
Au(13)-Au(20)-Au(14)	61.346(18)
Au(13)-Au(20)-Au(16)	120.84(3)
Au(13)-Au(20)-Au(21)	58.714(19)
Au(13)-Au(20)-Au(22)	85.01(2)
Au(14)-Au(20)-Au(11)	112.52(2)
Au(14)-Au(20)-Au(22)	111.08(2)
Au(16)-Au(20)-Au(1)	57.145(19)
Au(16)-Au(20)-Au(3)	114.91(2)
Au(16)-Au(20)-Au(11)	121.33(2)
Au(16)-Au(20)-Au(12)	177.20(3)
Au(16)-Au(20)-Au(14)	87.33(2)
Au(16)-Au(20)-Au(21)	62.124(19)
Au(16)-Au(20)-Au(22)	59.780(19)
Au(17)-Au(20)-Au(1)	57.066(18)
Au(17)-Au(20)-Au(3)	89.27(2)
Au(17)-Au(20)-Au(11)	63.47(2)
Au(17)-Au(20)-Au(12)	122.01(3)
Au(17)-Au(20)-Au(13)	179.46(3)
Au(17)-Au(20)-Au(14)	118.23(2)
Au(17)-Au(20)-Au(16)	58.69(2)
Au(17)-Au(20)-Au(21)	120.81(2)
Au(17)-Au(20)-Au(22)	94.88(2)
Au(17)-Au(20)-Au(25)	121.81(2)
Au(21)-Au(20)-Au(11)	169.62(2)
Au(21)-Au(20)-Au(14)	57.148(18)
Au(21)-Au(20)-Au(22)	53.970(19)

Au(22)-Au(20)-Au(11)	136.39(2)
Au(25)-Au(20)-Au(1)	178.06(3)
Au(25)-Au(20)-Au(3)	121.06(2)
Au(25)-Au(20)-Au(11)	93.81(2)
Au(25)-Au(20)-Au(12)	58.322(19)
Au(25)-Au(20)-Au(13)	58.614(19)
Au(25)-Au(20)-Au(14)	119.96(2)
Au(25)-Au(20)-Au(16)	123.99(3)
Au(25)-Au(20)-Au(21)	91.29(2)
Au(25)-Au(20)-Au(22)	64.71(2)
Au(13)-Au(21)-Au(14)	63.105(19)
Au(13)-Au(21)-Au(16)	115.56(3)
Au(13)-Au(21)-Au(20)	58.415(18)
Au(13)-Au(21)-Au(24)	60.013(19)
Au(13)-Au(21)-Au(30)	60.19(2)
Au(13)-Au(21)-Au(37)	175.01(3)
Au(14)-Au(21)-Au(16)	87.33(2)
Au(14)-Au(21)-Au(20)	62.13(2)
Au(14)-Au(21)-Au(24)	123.11(2)
Au(14)-Au(21)-Au(30)	93.15(2)
Au(14)-Au(21)-Au(37)	120.93(2)
Au(15)-Au(21)-Au(13)	123.30(3)
Au(15)-Au(21)-Au(14)	60.236(19)
Au(15)-Au(21)-Au(16)	60.470(19)
Au(15)-Au(21)-Au(20)	92.51(3)
Au(15)-Au(21)-Au(24)	176.43(3)
Au(15)-Au(21)-Au(30)	124.25(2)
Au(15)-Au(21)-Au(37)	60.933(19)
Au(16)-Au(21)-Au(24)	117.44(2)
Au(16)-Au(21)-Au(37)	68.44(2)
Au(20)-Au(21)-Au(16)	57.147(19)
Au(20)-Au(21)-Au(24)	88.39(2)
Au(20)-Au(21)-Au(37)	125.51(2)
Au(22)-Au(21)-Au(13)	91.13(2)
Au(22)-Au(21)-Au(14)	126.79(3)
Au(22)-Au(21)-Au(15)	121.05(3)
Au(22)-Au(21)-Au(16)	61.69(2)
Au(22)-Au(21)-Au(20)	64.71(2)

Au(22)-Au(21)-Au(24)	56.361(19)
Au(22)-Au(21)-Au(30)	114.12(2)
Au(22)-Au(21)-Au(37)	88.33(2)
Au(23)-Au(21)-Au(13)	115.53(2)
Au(23)-Au(21)-Au(14)	168.79(3)
Au(23)-Au(21)-Au(15)	120.40(3)
Au(23)-Au(21)-Au(16)	102.70(2)
Au(23)-Au(21)-Au(20)	127.66(2)
Au(23)-Au(21)-Au(22)	63.50(2)
Au(23)-Au(21)-Au(24)	56.62(2)
Au(23)-Au(21)-Au(30)	77.34(2)
Au(23)-Au(21)-Au(35)	109.25(3)
Au(23)-Au(21)-Au(37)	59.89(2)
Au(24)-Au(21)-Au(37)	115.83(2)
Au(30)-Au(21)-Au(16)	174.62(2)
Au(30)-Au(21)-Au(20)	118.51(2)
Au(30)-Au(21)-Au(24)	57.992(18)
Au(30)-Au(21)-Au(37)	115.62(3)
Au(35)-Au(21)-Au(13)	94.20(2)
Au(35)-Au(21)-Au(14)	60.63(2)
Au(35)-Au(21)-Au(15)	59.81(2)
Au(35)-Au(21)-Au(16)	120.19(2)
Au(35)-Au(21)-Au(20)	122.74(3)
Au(35)-Au(21)-Au(22)	172.45(3)
Au(35)-Au(21)-Au(24)	122.37(3)
Au(35)-Au(21)-Au(30)	64.44(2)
Au(35)-Au(21)-Au(37)	85.90(2)
Au(16)-Au(22)-Au(20)	56.733(19)
Au(16)-Au(22)-Au(25)	109.92(3)
Au(16)-Au(22)-Au(40)	76.43(2)
Au(20)-Au(22)-Au(25)	53.565(18)
Au(20)-Au(22)-Au(40)	75.46(2)
Au(21)-Au(22)-Au(16)	63.68(2)
Au(21)-Au(22)-Au(20)	61.32(2)
Au(21)-Au(22)-Au(23)	57.40(2)
Au(21)-Au(22)-Au(25)	88.81(2)
Au(21)-Au(22)-Au(40)	132.21(3)
Au(23)-Au(22)-Au(16)	100.05(2)

Au(23)-Au(22)-Au(20)	118.26(2)
Au(23)-Au(22)-Au(25)	117.24(2)
Au(23)-Au(22)-Au(40)	161.42(3)
Au(24)-Au(22)-Au(16)	130.12(3)
Au(24)-Au(22)-Au(20)	92.20(2)
Au(24)-Au(22)-Au(21)	67.24(2)
Au(24)-Au(22)-Au(23)	58.37(2)
Au(24)-Au(22)-Au(25)	60.07(2)
Au(24)-Au(22)-Au(40)	137.35(3)
Au(25)-Au(22)-Au(40)	80.71(2)
S(21)-Au(22)-Au(16)	111.05(10)
S(21)-Au(22)-Au(20)	118.95(11)
S(21)-Au(22)-Au(21)	173.99(9)
S(21)-Au(22)-Au(23)	122.79(11)
S(21)-Au(22)-Au(24)	118.38(9)
S(21)-Au(22)-Au(25)	95.95(9)
S(21)-Au(22)-Au(40)	45.61(11)
Au(21)-Au(23)-Au(22)	59.10(2)
Au(21)-Au(23)-Au(24)	68.04(2)
Au(21)-Au(23)-Au(34)	77.63(2)
Au(21)-Au(23)-Au(37)	68.35(2)
Au(21)-Au(23)-Au(39)	127.51(3)
Au(22)-Au(23)-Au(34)	136.67(3)
Au(22)-Au(23)-Au(37)	90.63(3)
Au(22)-Au(23)-Au(39)	121.76(2)
Au(24)-Au(23)-Au(22)	58.63(2)
Au(24)-Au(23)-Au(34)	109.11(3)
Au(24)-Au(23)-Au(37)	135.35(3)
Au(24)-Au(23)-Au(39)	71.75(2)
Au(34)-Au(23)-Au(39)	85.27(2)
Au(37)-Au(23)-Au(34)	69.94(2)
Au(37)-Au(23)-Au(39)	147.57(3)
S(19)-Au(23)-Au(21)	167.24(10)
S(19)-Au(23)-Au(22)	132.55(10)
S(19)-Au(23)-Au(24)	111.84(9)
S(19)-Au(23)-Au(34)	90.73(10)
S(19)-Au(23)-Au(37)	112.80(9)
S(19)-Au(23)-Au(39)	44.93(9)

Au(13)-Au(24)-Au(21)	56.975(18)
Au(13)-Au(24)-Au(25)	55.69(2)
Au(13)-Au(24)-Au(27)	70.39(2)
Au(21)-Au(24)-Au(27)	127.16(3)
Au(22)-Au(24)-Au(13)	89.18(3)
Au(22)-Au(24)-Au(21)	56.401(19)
Au(22)-Au(24)-Au(25)	66.77(2)
Au(22)-Au(24)-Au(27)	123.00(3)
Au(22)-Au(24)-Au(30)	115.26(2)
Au(23)-Au(24)-Au(13)	111.29(2)
Au(23)-Au(24)-Au(21)	55.34(2)
Au(23)-Au(24)-Au(22)	63.00(2)
Au(23)-Au(24)-Au(25)	128.25(3)
Au(23)-Au(24)-Au(27)	174.00(3)
Au(23)-Au(24)-Au(30)	77.30(2)
Au(25)-Au(24)-Au(21)	86.95(2)
Au(25)-Au(24)-Au(27)	57.619(19)
Au(30)-Au(24)-Au(13)	59.51(2)
Au(30)-Au(24)-Au(21)	59.100(19)
Au(30)-Au(24)-Au(25)	115.17(3)
Au(30)-Au(24)-Au(27)	99.25(2)
S(20)-Au(24)-Au(13)	115.32(10)
S(20)-Au(24)-Au(21)	168.29(9)
S(20)-Au(24)-Au(22)	134.89(9)
S(20)-Au(24)-Au(23)	129.05(10)
S(20)-Au(24)-Au(25)	95.36(10)
S(20)-Au(24)-Au(27)	47.24(10)
S(20)-Au(24)-Au(30)	109.83(9)
Au(12)-Au(25)-Au(13)	60.63(2)
Au(12)-Au(25)-Au(20)	62.04(2)
Au(12)-Au(25)-Au(22)	122.96(2)
Au(12)-Au(25)-Au(24)	122.38(3)
Au(12)-Au(25)-Au(26)	83.91(3)
Au(12)-Au(25)-Au(27)	98.70(2)
Au(13)-Au(25)-Au(20)	61.53(2)
Au(13)-Au(25)-Au(22)	84.85(2)
Au(13)-Au(25)-Au(24)	61.83(2)
Au(13)-Au(25)-Au(26)	139.52(3)

Au(13)-Au(25)-Au(27)	75.91(2)
Au(20)-Au(25)-Au(22)	61.727(19)
Au(20)-Au(25)-Au(24)	93.11(2)
Au(20)-Au(25)-Au(26)	85.71(2)
Au(20)-Au(25)-Au(27)	137.44(3)
Au(24)-Au(25)-Au(22)	53.154(18)
Au(24)-Au(25)-Au(26)	149.33(3)
Au(26)-Au(25)-Au(22)	100.73(2)
Au(27)-Au(25)-Au(22)	116.21(3)
Au(27)-Au(25)-Au(24)	64.26(2)
Au(27)-Au(25)-Au(26)	132.33(2)
S(7)-Au(25)-Au(12)	127.48(11)
S(7)-Au(25)-Au(13)	171.14(11)
S(7)-Au(25)-Au(20)	123.96(9)
S(7)-Au(25)-Au(22)	92.06(9)
S(7)-Au(25)-Au(24)	109.78(11)
S(7)-Au(25)-Au(26)	49.23(11)
S(7)-Au(25)-Au(27)	98.18(10)
Au(25)-Au(26)-Au(11)	84.91(2)
Au(25)-Au(26)-Au(40)	83.69(2)
Au(40)-Au(26)-Au(11)	91.73(2)
S(6)-Au(26)-Au(11)	45.67(9)
S(6)-Au(26)-Au(25)	129.43(10)
S(6)-Au(26)-Au(40)	104.30(9)
S(6)-Au(26)-S(7)	174.09(13)
S(7)-Au(26)-Au(11)	137.23(9)
S(7)-Au(26)-Au(25)	52.47(9)
S(7)-Au(26)-Au(40)	81.26(9)
Au(25)-Au(27)-Au(24)	58.12(2)
Au(25)-Au(27)-Au(29)	97.35(2)
Au(29)-Au(27)-Au(24)	73.02(2)
S(8)-Au(27)-Au(24)	137.72(9)
S(8)-Au(27)-Au(25)	86.94(9)
S(8)-Au(27)-Au(29)	90.61(10)
S(8)-Au(27)-S(20)	172.91(13)
S(20)-Au(27)-Au(24)	48.90(9)
S(20)-Au(27)-Au(25)	97.14(9)
S(20)-Au(27)-Au(29)	94.60(9)

Au(12)-Au(28)-Au(13)	53.162(18)
Au(12)-Au(28)-Au(32)	56.205(17)
Au(13)-Au(28)-Au(32)	56.061(17)
Au(29)-Au(28)-Au(12)	103.90(2)
Au(29)-Au(28)-Au(13)	51.494(18)
Au(29)-Au(28)-Au(32)	89.29(2)
S(8)-Au(28)-Au(12)	83.48(9)
S(8)-Au(28)-Au(13)	94.84(9)
S(8)-Au(28)-Au(29)	93.23(11)
S(8)-Au(28)-Au(32)	138.85(9)
S(9)-Au(28)-Au(12)	103.27(10)
S(9)-Au(28)-Au(13)	89.05(9)
S(9)-Au(28)-Au(29)	84.86(11)
S(9)-Au(28)-Au(32)	47.73(9)
S(9)-Au(28)-S(8)	173.24(14)
Au(13)-Au(29)-Au(27)	75.15(2)
Au(13)-Au(29)-Au(28)	69.74(2)
Au(13)-Au(29)-Au(30)	64.68(2)
Au(13)-Au(29)-Au(31)	60.01(2)
Au(13)-Au(29)-Au(38)	127.39(3)
Au(27)-Au(29)-Au(38)	77.42(2)
Au(28)-Au(29)-Au(27)	72.43(2)
Au(28)-Au(29)-Au(38)	139.31(3)
Au(30)-Au(29)-Au(27)	105.33(2)
Au(30)-Au(29)-Au(28)	133.01(3)
Au(30)-Au(29)-Au(31)	58.69(2)
Au(30)-Au(29)-Au(38)	80.91(2)
Au(31)-Au(29)-Au(27)	135.10(3)
Au(31)-Au(29)-Au(28)	89.16(3)
Au(31)-Au(29)-Au(38)	131.49(3)
S(17)-Au(29)-Au(13)	161.21(10)
S(17)-Au(29)-Au(27)	86.25(10)
S(17)-Au(29)-Au(28)	102.42(11)
S(17)-Au(29)-Au(30)	124.49(11)
S(17)-Au(29)-Au(31)	138.37(10)
S(17)-Au(29)-Au(38)	48.27(11)
Au(13)-Au(30)-Au(21)	58.72(2)
Au(13)-Au(30)-Au(34)	131.00(3)

Au(13)-Au(30)-Au(35)	87.50(2)
Au(21)-Au(30)-Au(34)	72.56(2)
Au(21)-Au(30)-Au(35)	54.708(18)
Au(24)-Au(30)-Au(13)	61.26(2)
Au(24)-Au(30)-Au(21)	62.907(19)
Au(24)-Au(30)-Au(34)	102.19(2)
Au(24)-Au(30)-Au(35)	117.56(2)
Au(29)-Au(30)-Au(13)	57.09(2)
Au(29)-Au(30)-Au(21)	115.54(3)
Au(29)-Au(30)-Au(24)	82.14(2)
Au(29)-Au(30)-Au(31)	62.94(2)
Au(29)-Au(30)-Au(34)	171.89(3)
Au(29)-Au(30)-Au(35)	125.96(3)
Au(31)-Au(30)-Au(13)	58.94(2)
Au(31)-Au(30)-Au(21)	89.16(3)
Au(31)-Au(30)-Au(24)	120.06(3)
Au(31)-Au(30)-Au(34)	119.21(2)
Au(31)-Au(30)-Au(35)	63.89(2)
Au(35)-Au(30)-Au(34)	58.23(2)
S(12)-Au(30)-Au(13)	168.34(9)
S(12)-Au(30)-Au(21)	117.08(9)
S(12)-Au(30)-Au(24)	107.09(9)
S(12)-Au(30)-Au(29)	124.52(9)
S(12)-Au(30)-Au(31)	132.70(9)
S(12)-Au(30)-Au(34)	47.79(9)
S(12)-Au(30)-Au(35)	98.52(9)
Au(13)-Au(31)-Au(14)	61.629(19)
Au(13)-Au(31)-Au(29)	57.14(2)
Au(13)-Au(31)-Au(32)	63.80(2)
Au(13)-Au(31)-Au(33)	130.18(3)
Au(13)-Au(31)-Au(35)	89.06(2)
Au(14)-Au(31)-Au(33)	73.31(2)
Au(14)-Au(31)-Au(35)	55.183(18)
Au(29)-Au(31)-Au(14)	118.73(2)
Au(29)-Au(31)-Au(32)	96.70(2)
Au(29)-Au(31)-Au(33)	158.18(3)
Au(29)-Au(31)-Au(35)	120.18(3)
Au(30)-Au(31)-Au(13)	63.45(2)

Au(30)-Au(31)-Au(14)	93.09(2)
Au(30)-Au(31)-Au(29)	58.36(2)
Au(30)-Au(31)-Au(32)	126.92(3)
Au(30)-Au(31)-Au(33)	142.59(2)
Au(30)-Au(31)-Au(35)	62.59(2)
Au(32)-Au(31)-Au(14)	56.289(19)
Au(32)-Au(31)-Au(33)	74.39(2)
Au(32)-Au(31)-Au(35)	111.19(2)
Au(35)-Au(31)-Au(33)	81.61(2)
S(10)-Au(31)-Au(13)	173.27(11)
S(10)-Au(31)-Au(14)	112.19(10)
S(10)-Au(31)-Au(29)	129.07(10)
S(10)-Au(31)-Au(30)	121.08(10)
S(10)-Au(31)-Au(32)	110.98(11)
S(10)-Au(31)-Au(33)	43.11(10)
S(10)-Au(31)-Au(35)	89.09(9)
Au(3)-Au(32)-Au(9)	67.22(2)
Au(3)-Au(32)-Au(12)	64.067(19)
Au(3)-Au(32)-Au(13)	90.56(2)
Au(3)-Au(32)-Au(14)	62.53(2)
Au(3)-Au(32)-Au(28)	121.35(2)
Au(3)-Au(32)-Au(31)	125.07(2)
Au(4)-Au(32)-Au(3)	59.766(19)
Au(4)-Au(32)-Au(9)	91.49(3)
Au(4)-Au(32)-Au(12)	123.79(3)
Au(4)-Au(32)-Au(13)	121.66(2)
Au(4)-Au(32)-Au(14)	60.64(2)
Au(4)-Au(32)-Au(28)	176.54(3)
Au(4)-Au(32)-Au(31)	100.20(2)
Au(9)-Au(32)-Au(28)	86.17(2)
Au(12)-Au(32)-Au(9)	68.45(2)
Au(12)-Au(32)-Au(13)	55.583(19)
Au(12)-Au(32)-Au(28)	57.576(18)
Au(12)-Au(32)-Au(31)	109.82(3)
Au(13)-Au(32)-Au(9)	123.87(2)
Au(13)-Au(32)-Au(28)	61.799(18)
Au(14)-Au(32)-Au(9)	129.63(3)
Au(14)-Au(32)-Au(12)	92.10(2)

Au(14)-Au(32)-Au(13)	61.123(19)
Au(14)-Au(32)-Au(28)	122.82(3)
Au(14)-Au(32)-Au(31)	63.33(2)
Au(31)-Au(32)-Au(9)	166.29(2)
Au(31)-Au(32)-Au(13)	55.065(19)
Au(31)-Au(32)-Au(28)	81.82(2)
S(9)-Au(32)-Au(3)	161.02(10)
S(9)-Au(32)-Au(4)	132.43(10)
S(9)-Au(32)-Au(9)	95.93(11)
S(9)-Au(32)-Au(12)	102.44(9)
S(9)-Au(32)-Au(13)	92.09(11)
S(9)-Au(32)-Au(14)	134.20(11)
S(9)-Au(32)-Au(28)	45.51(9)
S(9)-Au(32)-Au(31)	70.89(11)
Au(4)-Au(33)-Au(31)	85.77(2)
Au(4)-Au(33)-Au(36)	91.42(2)
Au(36)-Au(33)-Au(31)	90.67(2)
S(10)-Au(33)-Au(4)	130.38(9)
S(10)-Au(33)-Au(31)	44.88(9)
S(10)-Au(33)-Au(36)	93.53(9)
S(10)-Au(33)-S(11)	174.41(13)
S(11)-Au(33)-Au(4)	51.61(9)
S(11)-Au(33)-Au(31)	137.36(9)
S(11)-Au(33)-Au(36)	91.59(9)
Au(23)-Au(34)-Au(30)	70.79(2)
Au(35)-Au(34)-Au(23)	96.04(3)
Au(35)-Au(34)-Au(30)	59.77(2)
S(12)-Au(34)-Au(23)	90.30(9)
S(12)-Au(34)-Au(30)	49.49(11)
S(12)-Au(34)-Au(35)	101.34(10)
S(12)-Au(34)-S(15)	170.17(15)
S(15)-Au(34)-Au(23)	95.18(10)
S(15)-Au(34)-Au(30)	140.22(11)
S(15)-Au(34)-Au(35)	86.22(11)
Au(14)-Au(35)-Au(30)	91.86(2)
Au(14)-Au(35)-Au(31)	62.268(19)
Au(14)-Au(35)-Au(34)	138.73(3)
Au(14)-Au(35)-Au(36)	87.04(2)

Au(15)-Au(35)-Au(14)	61.07(2)
Au(15)-Au(35)-Au(30)	121.86(2)
Au(15)-Au(35)-Au(31)	122.85(3)
Au(15)-Au(35)-Au(34)	104.28(3)
Au(15)-Au(35)-Au(36)	80.54(2)
Au(21)-Au(35)-Au(14)	61.97(2)
Au(21)-Au(35)-Au(15)	61.01(2)
Au(21)-Au(35)-Au(30)	60.849(19)
Au(21)-Au(35)-Au(31)	86.56(2)
Au(21)-Au(35)-Au(34)	77.08(2)
Au(21)-Au(35)-Au(36)	138.54(3)
Au(30)-Au(35)-Au(31)	53.513(18)
Au(34)-Au(35)-Au(30)	62.00(2)
Au(34)-Au(35)-Au(31)	112.96(2)
Au(34)-Au(35)-Au(36)	130.87(3)
Au(36)-Au(35)-Au(30)	153.34(2)
Au(36)-Au(35)-Au(31)	103.48(2)
S(14)-Au(35)-Au(14)	127.89(10)
S(14)-Au(35)-Au(15)	122.92(9)
S(14)-Au(35)-Au(21)	170.07(11)
S(14)-Au(35)-Au(30)	114.37(10)
S(14)-Au(35)-Au(31)	97.28(9)
S(14)-Au(35)-Au(34)	93.00(10)
S(14)-Au(35)-Au(36)	49.44(10)
Au(35)-Au(36)-Au(33)	84.02(2)
S(13)-Au(36)-Au(33)	88.75(9)
S(13)-Au(36)-Au(35)	127.25(9)
S(13)-Au(36)-S(14)	176.67(13)
S(14)-Au(36)-Au(33)	87.92(9)
S(14)-Au(36)-Au(35)	52.47(9)
Au(15)-Au(37)-Au(21)	53.035(17)
Au(23)-Au(37)-Au(15)	104.49(2)
Au(23)-Au(37)-Au(21)	51.763(18)
S(15)-Au(37)-Au(15)	92.09(11)
S(15)-Au(37)-Au(21)	100.85(9)
S(15)-Au(37)-Au(23)	95.29(10)
S(16)-Au(37)-Au(15)	93.92(10)
S(16)-Au(37)-Au(21)	86.87(9)

S(16)-Au(37)-Au(23)	88.17(9)
S(16)-Au(37)-S(15)	172.10(13)
S(17)-Au(38)-Au(29)	48.71(9)
S(18)-Au(38)-Au(29)	138.54(11)
S(18)-Au(38)-S(17)	171.93(14)
S(18)-Au(39)-Au(23)	140.50(12)
S(19)-Au(39)-Au(23)	46.16(11)
S(19)-Au(39)-S(18)	172.85(17)
Au(17)-Au(40)-Au(22)	86.43(2)
Au(17)-Au(40)-Au(26)	85.76(2)
Au(26)-Au(40)-Au(22)	94.87(2)
S(21)-Au(40)-Au(17)	132.85(9)
S(21)-Au(40)-Au(22)	47.91(9)
S(21)-Au(40)-Au(26)	106.22(9)
S(21)-Au(40)-S(22)	171.19(13)
S(22)-Au(40)-Au(17)	50.98(9)
S(22)-Au(40)-Au(22)	137.35(9)
S(22)-Au(40)-Au(26)	81.32(9)
S(24)-Au(41)-Au(10)	47.55(11)
S(25)-Au(41)-Au(10)	141.10(13)
S(25)-Au(41)-S(24)	171.23(17)
S(25)-Au(42)-Au(6)	139.92(11)
S(25)-Au(42)-S(26)	172.92(15)
S(26)-Au(42)-Au(6)	47.05(10)
Au(8)-S(1)-Au(7)	80.22(12)
C(250)-S(1)-Au(7)	110.3(6)
C(250)-S(1)-Au(8)	104.8(5)
Au(9)-S(2)-Au(8)	98.43(14)
C(240)-S(2)-Au(8)	101.2(5)
C(240)-S(2)-Au(9)	107.8(5)
Au(18)-S(3)-Au(2)	82.82(14)
C(1)-S(3)-Au(2)	108.8(5)
C(1)-S(3)-Au(18)	105.9(5)
Au(18)-S(4)-Au(19)	100.82(15)
C(21)-S(4)-Au(18)	101.9(6)
C(21)-S(4)-Au(19)	107.5(6)
Au(19)-S(5)-Au(15)	92.80(13)
C(11)-S(5)-Au(15)	113.3(6)

C(11)-S(5)-Au(19)	110.0(5)
Au(26)-S(6)-Au(11)	90.81(15)
C(31)-S(6)-Au(11)	106.3(5)
C(31)-S(6)-Au(26)	104.5(5)
Au(26)-S(7)-Au(25)	78.30(12)
C(41)-S(7)-Au(25)	115.1(5)
C(41)-S(7)-Au(26)	104.3(5)
Au(27)-S(8)-Au(28)	99.74(14)
C(161)-S(8)-Au(27)	106.4(6)
C(161)-S(8)-Au(28)	106.1(5)
Au(28)-S(9)-Au(32)	86.76(13)
C(51)-S(9)-Au(28)	110.8(5)
C(51)-S(9)-Au(32)	108.0(5)
Au(33)-S(10)-Au(31)	92.01(14)
C(71)-S(10)-Au(31)	110.9(5)
C(71)-S(10)-Au(33)	100.5(5)
Au(33)-S(11)-Au(4)	79.31(11)
C(61)-S(11)-Au(4)	110.9(5)
C(61)-S(11)-Au(33)	108.0(5)
Au(34)-S(12)-Au(30)	82.72(14)
C(121)-S(12)-Au(30)	112.9(6)
C(121)-S(12)-Au(34)	101.4(5)
Au(36)-S(13)-Au(5)	97.45(13)
C(81)-S(13)-Au(5)	100.9(5)
C(81)-S(13)-Au(36)	109.9(6)
Au(36)-S(14)-Au(35)	78.09(13)
C(91)-S(14)-Au(35)	110.7(5)
C(91)-S(14)-Au(36)	109.0(5)
Au(34)-S(15)-Au(37)	94.50(14)
C(111)-S(15)-Au(34)	104.3(5)
C(111)-S(15)-Au(37)	107.5(6)
Au(37)-S(16)-Au(16)	95.09(13)
C(101)-S(16)-Au(16)	109.3(5)
C(101)-S(16)-Au(37)	107.4(5)
Au(38)-S(17)-Au(29)	83.02(12)
C(151)-S(17)-Au(29)	107.1(5)
C(151)-S(17)-Au(38)	104.3(5)
Au(38)-S(18)-Au(39)	113.52(19)

C(141)-S(18)-Au(38)	106.6(5)
C(141)-S(18)-Au(39)	102.9(5)
Au(39)-S(19)-Au(23)	88.91(14)
C(131)-S(19)-Au(23)	103.6(5)
C(131)-S(19)-Au(39)	106.1(4)
Au(27)-S(20)-Au(24)	83.86(14)
C(171)-S(20)-Au(24)	111.4(5)
C(171)-S(20)-Au(27)	106.5(6)
Au(40)-S(21)-Au(22)	86.48(13)
C(191)-S(21)-Au(22)	106.9(5)
C(191)-S(21)-Au(40)	103.4(6)
Au(40)-S(22)-Au(17)	79.87(13)
C(181)-S(22)-Au(17)	108.4(5)
C(181)-S(22)-Au(40)	103.2(6)
Au(9)-S(23)-Au(12)	90.84(13)
C(201)-S(23)-Au(9)	108.9(5)
C(201)-S(23)-Au(12)	114.2(5)
Au(41)-S(24)-Au(10)	86.15(14)
C(230)-S(24)-Au(10)	106.3(6)
C(230)-S(24)-Au(41)	103.5(5)
Au(42)-S(25)-Au(41)	111.7(2)
C(221)-S(25)-Au(41)	102.2(5)
C(221)-S(25)-Au(42)	107.3(5)
Au(42)-S(26)-Au(6)	87.15(13)
C(211)-S(26)-Au(6)	111.5(5)
C(211)-S(26)-Au(42)	97.3(6)
C(2)-C(1)-S(3)	117.9(10)
C(6)-C(1)-S(3)	124.3(11)
C(6)-C(1)-C(2)	117.4(13)
C(1)-C(2)-H(2)	120.2
C(3)-C(2)-C(1)	119.5(14)
C(3)-C(2)-H(2)	120.2
C(2)-C(3)-H(3)	119.6
C(4)-C(3)-C(2)	120.8(16)
C(4)-C(3)-H(3)	119.6
C(3)-C(4)-C(7)	117.7(15)
C(5)-C(4)-C(3)	120.1(16)
C(5)-C(4)-C(7)	122.2(15)

C(4)-C(5)-H(5)	120.1
C(4)-C(5)-C(6)	119.7(15)
C(6)-C(5)-H(5)	120.1
C(1)-C(6)-C(5)	122.0(15)
C(1)-C(6)-H(6)	119.0
C(5)-C(6)-H(6)	119.0
C(8)-C(7)-C(4)	110.3(15)
C(8)-C(7)-C(10)	105.9(19)
C(9)-C(7)-C(4)	107.8(17)
C(9)-C(7)-C(8)	109.6(19)
C(9)-C(7)-C(10)	113(2)
C(10)-C(7)-C(4)	110.6(19)
C(7)-C(8)-H(8A)	109.5
C(7)-C(8)-H(8B)	109.5
C(7)-C(8)-H(8C)	109.5
H(8A)-C(8)-H(8B)	109.5
H(8A)-C(8)-H(8C)	109.5
H(8B)-C(8)-H(8C)	109.5
C(7)-C(9)-H(9A)	109.5
C(7)-C(9)-H(9B)	109.5
C(7)-C(9)-H(9C)	109.5
H(9A)-C(9)-H(9B)	109.5
H(9A)-C(9)-H(9C)	109.5
H(9B)-C(9)-H(9C)	109.5
C(7)-C(10)-H(10A)	109.5
C(7)-C(10)-H(10B)	109.5
C(7)-C(10)-H(10C)	109.5
H(10A)-C(10)-H(10B)	109.5
H(10A)-C(10)-H(10C)	109.5
H(10B)-C(10)-H(10C)	109.5
C(12)-C(11)-S(5)	121.7(14)
C(12)-C(11)-C(16)	118.7(15)
C(16)-C(11)-S(5)	119.2(11)
C(11)-C(12)-H(12)	120.1
C(11)-C(12)-C(13)	119.7(19)
C(13)-C(12)-H(12)	120.1
C(12)-C(13)-H(13)	118.8
C(14)-C(13)-C(12)	122.3(19)

C(14)-C(13)-H(13)	118.8
C(13)-C(14)-C(15)	117(2)
C(13)-C(14)-C(17)	123.4(19)
C(15)-C(14)-C(17)	119(2)
C(14)-C(15)-H(15)	119.8
C(14)-C(15)-C(16)	120.3(19)
C(16)-C(15)-H(15)	119.8
C(11)-C(16)-C(15)	121.4(15)
C(11)-C(16)-H(16)	119.3
C(15)-C(16)-H(16)	119.3
C(18)-C(17)-C(14)	105(2)
C(19)-C(17)-C(14)	108(2)
C(19)-C(17)-C(18)	104(2)
C(19)-C(17)-C(20)	122(3)
C(20)-C(17)-C(14)	117(2)
C(20)-C(17)-C(18)	97(3)
C(17)-C(18)-H(18A)	109.5
C(17)-C(18)-H(18B)	109.5
C(17)-C(18)-H(18C)	109.5
H(18A)-C(18)-H(18B)	109.5
H(18A)-C(18)-H(18C)	109.5
H(18B)-C(18)-H(18C)	109.5
C(17)-C(19)-H(19A)	109.5
C(17)-C(19)-H(19B)	109.5
C(17)-C(19)-H(19C)	109.5
H(19A)-C(19)-H(19B)	109.5
H(19A)-C(19)-H(19C)	109.5
H(19B)-C(19)-H(19C)	109.5
C(17)-C(20)-H(20A)	109.5
C(17)-C(20)-H(20B)	109.5
C(17)-C(20)-H(20C)	109.5
H(20A)-C(20)-H(20B)	109.5
H(20A)-C(20)-H(20C)	109.5
H(20B)-C(20)-H(20C)	109.5
C(22)-C(21)-S(4)	124.4(15)
C(22)-C(21)-C(26)	119.8(18)
C(26)-C(21)-S(4)	115.7(14)
C(21)-C(22)-H(22)	120.1

C(21)-C(22)-C(23)	120(2)
C(23)-C(22)-H(22)	120.1
C(22)-C(23)-H(23)	120.1
C(24)-C(23)-C(22)	120(2)
C(24)-C(23)-H(23)	120.1
C(23)-C(24)-C(27)	117(3)
C(25)-C(24)-C(23)	121(2)
C(25)-C(24)-C(27)	122(3)
C(24)-C(25)-H(25)	119.9
C(24)-C(25)-C(26)	120(2)
C(26)-C(25)-H(25)	119.9
C(21)-C(26)-C(25)	119(2)
C(21)-C(26)-H(26)	120.4
C(25)-C(26)-H(26)	120.4
C(28)-C(27)-C(24)	114(3)
C(28)-C(27)-C(29)	103.2(14)
C(29)-C(27)-C(24)	115(3)
C(30)-C(27)-C(24)	113(3)
C(30)-C(27)-C(28)	106.0(15)
C(30)-C(27)-C(29)	105.5(14)
C(27)-C(28)-H(28A)	109.5
C(27)-C(28)-H(28B)	109.5
C(27)-C(28)-H(28C)	109.5
H(28A)-C(28)-H(28B)	109.5
H(28A)-C(28)-H(28C)	109.5
H(28B)-C(28)-H(28C)	109.5
C(27)-C(29)-H(29A)	109.5
C(27)-C(29)-H(29B)	109.5
C(27)-C(29)-H(29C)	109.5
H(29A)-C(29)-H(29B)	109.5
H(29A)-C(29)-H(29C)	109.5
H(29B)-C(29)-H(29C)	109.5
C(27)-C(30)-H(30A)	109.5
C(27)-C(30)-H(30B)	109.5
C(27)-C(30)-H(30C)	109.5
H(30A)-C(30)-H(30B)	109.5
H(30A)-C(30)-H(30C)	109.5
H(30B)-C(30)-H(30C)	109.5

C(32)-C(31)-S(6)	121.5(12)
C(32)-C(31)-C(36)	119.0(13)
C(36)-C(31)-S(6)	119.5(11)
C(31)-C(32)-H(32)	120.1
C(31)-C(32)-C(33)	119.7(16)
C(33)-C(32)-H(32)	120.1
C(32)-C(33)-H(33)	118.8
C(34)-C(33)-C(32)	122.4(15)
C(34)-C(33)-H(33)	118.8
C(33)-C(34)-C(35)	117.6(15)
C(33)-C(34)-C(37)	124.5(14)
C(35)-C(34)-C(37)	117.8(15)
C(34)-C(35)-H(35)	120.0
C(36)-C(35)-C(34)	120.1(17)
C(36)-C(35)-H(35)	120.0
C(31)-C(36)-H(36)	119.4
C(35)-C(36)-C(31)	121.2(14)
C(35)-C(36)-H(36)	119.4
C(34)-C(37)-C(38)	110.7(14)
C(34)-C(37)-C(39)	112.9(15)
C(38)-C(37)-C(39)	106.7(14)
C(40)-C(37)-C(34)	110.1(15)
C(40)-C(37)-C(38)	108.6(16)
C(40)-C(37)-C(39)	107.8(14)
C(37)-C(38)-H(38A)	109.5
C(37)-C(38)-H(38B)	109.5
C(37)-C(38)-H(38C)	109.5
H(38A)-C(38)-H(38B)	109.5
H(38A)-C(38)-H(38C)	109.5
H(38B)-C(38)-H(38C)	109.5
C(37)-C(39)-H(39A)	109.5
C(37)-C(39)-H(39B)	109.5
C(37)-C(39)-H(39C)	109.5
H(39A)-C(39)-H(39B)	109.5
H(39A)-C(39)-H(39C)	109.5
H(39B)-C(39)-H(39C)	109.5
C(37)-C(40)-H(40A)	109.5
C(37)-C(40)-H(40B)	109.5

C(37)-C(40)-H(40C)	109.5
H(40A)-C(40)-H(40B)	109.5
H(40A)-C(40)-H(40C)	109.5
H(40B)-C(40)-H(40C)	109.5
C(42)-C(41)-S(7)	122.2(12)
C(42)-C(41)-C(46)	117.4(14)
C(46)-C(41)-S(7)	120.1(12)
C(41)-C(42)-H(42)	120.1
C(41)-C(42)-C(43)	119.9(16)
C(43)-C(42)-H(42)	120.1
C(42)-C(43)-H(43)	118.8
C(44)-C(43)-C(42)	122.4(16)
C(44)-C(43)-H(43)	118.8
C(43)-C(44)-C(45)	117.6(16)
C(43)-C(44)-C(47)	120.0(16)
C(45)-C(44)-C(47)	122.2(17)
C(44)-C(45)-H(45)	119.7
C(46)-C(45)-C(44)	120.7(19)
C(46)-C(45)-H(45)	119.7
C(41)-C(46)-H(46)	119.1
C(45)-C(46)-C(41)	121.8(17)
C(45)-C(46)-H(46)	119.1
C(48)-C(47)-C(44)	113.8(18)
C(48)-C(47)-C(49)	107(2)
C(49)-C(47)-C(44)	108.3(17)
C(50)-C(47)-C(44)	109.5(19)
C(50)-C(47)-C(48)	108(2)
C(50)-C(47)-C(49)	110(2)
C(47)-C(48)-H(48A)	109.5
C(47)-C(48)-H(48B)	109.5
C(47)-C(48)-H(48C)	109.5
H(48A)-C(48)-H(48B)	109.5
H(48A)-C(48)-H(48C)	109.5
H(48B)-C(48)-H(48C)	109.5
C(47)-C(49)-H(49A)	109.5
C(47)-C(49)-H(49B)	109.5
C(47)-C(49)-H(49C)	109.5
H(49A)-C(49)-H(49B)	109.5

H(49A)-C(49)-H(49C)	109.5
H(49B)-C(49)-H(49C)	109.5
C(47)-C(50)-H(50A)	109.5
C(47)-C(50)-H(50B)	109.5
C(47)-C(50)-H(50C)	109.5
H(50A)-C(50)-H(50B)	109.5
H(50A)-C(50)-H(50C)	109.5
H(50B)-C(50)-H(50C)	109.5
C(52)-C(51)-S(9)	118.2(12)
C(56)-C(51)-S(9)	126.0(12)
C(56)-C(51)-C(52)	115.8(15)
C(51)-C(52)-H(52)	119.4
C(53)-C(52)-C(51)	121.1(15)
C(53)-C(52)-H(52)	119.4
C(52)-C(53)-H(53)	119.1
C(52)-C(53)-C(54)	121.8(16)
C(54)-C(53)-H(53)	119.1
C(53)-C(54)-C(57)	123.0(15)
C(55)-C(54)-C(53)	116.8(16)
C(55)-C(54)-C(57)	120.1(15)
C(54)-C(55)-H(55)	118.7
C(54)-C(55)-C(56)	122.6(17)
C(56)-C(55)-H(55)	118.7
C(51)-C(56)-H(56)	119.0
C(55)-C(56)-C(51)	121.9(16)
C(55)-C(56)-H(56)	119.0
C(58)-C(57)-C(54)	111.4(16)
C(58)-C(57)-C(59)	106.7(18)
C(58)-C(57)-C(60)	108.4(16)
C(59)-C(57)-C(54)	111.1(15)
C(59)-C(57)-C(60)	110.8(16)
C(60)-C(57)-C(54)	108.5(17)
C(57)-C(58)-H(58A)	109.5
C(57)-C(58)-H(58B)	109.5
C(57)-C(58)-H(58C)	109.5
H(58A)-C(58)-H(58B)	109.5
H(58A)-C(58)-H(58C)	109.5
H(58B)-C(58)-H(58C)	109.5

C(57)-C(59)-H(59A)	109.5
C(57)-C(59)-H(59B)	109.5
C(57)-C(59)-H(59C)	109.5
H(59A)-C(59)-H(59B)	109.5
H(59A)-C(59)-H(59C)	109.5
H(59B)-C(59)-H(59C)	109.5
C(57)-C(60)-H(60A)	109.5
C(57)-C(60)-H(60B)	109.5
C(57)-C(60)-H(60C)	109.5
H(60A)-C(60)-H(60B)	109.5
H(60A)-C(60)-H(60C)	109.5
H(60B)-C(60)-H(60C)	109.5
C(62)-C(61)-S(11)	121.2(13)
C(66)-C(61)-S(11)	118.2(12)
C(66)-C(61)-C(62)	119.7(15)
C(61)-C(62)-H(62)	120.9
C(63)-C(62)-C(61)	118.1(16)
C(63)-C(62)-H(62)	120.9
C(62)-C(63)-H(63)	117.6
C(64)-C(63)-C(62)	124.8(15)
C(64)-C(63)-H(63)	117.6
C(63)-C(64)-C(65)	113.5(15)
C(63)-C(64)-C(67)	126.9(16)
C(65)-C(64)-C(67)	119.5(16)
C(64)-C(65)-H(65)	119.4
C(66)-C(65)-C(64)	121.2(16)
C(66)-C(65)-H(65)	119.4
C(61)-C(66)-C(65)	122.1(15)
C(61)-C(66)-H(66)	118.9
C(65)-C(66)-H(66)	118.9
C(64)-C(67)-C(68)	107.2(14)
C(64)-C(67)-C(69)	109.8(17)
C(69)-C(67)-C(68)	104.8(15)
C(70)-C(67)-C(64)	113.7(16)
C(70)-C(67)-C(68)	111.2(18)
C(70)-C(67)-C(69)	109.7(16)
C(67)-C(68)-H(68A)	109.5
C(67)-C(68)-H(68B)	109.5

C(67)-C(68)-H(68C)	109.5
H(68A)-C(68)-H(68B)	109.5
H(68A)-C(68)-H(68C)	109.5
H(68B)-C(68)-H(68C)	109.5
C(67)-C(69)-H(69A)	109.5
C(67)-C(69)-H(69B)	109.5
C(67)-C(69)-H(69C)	109.5
H(69A)-C(69)-H(69B)	109.5
H(69A)-C(69)-H(69C)	109.5
H(69B)-C(69)-H(69C)	109.5
C(67)-C(70)-H(70A)	109.5
C(67)-C(70)-H(70B)	109.5
C(67)-C(70)-H(70C)	109.5
H(70A)-C(70)-H(70B)	109.5
H(70A)-C(70)-H(70C)	109.5
H(70B)-C(70)-H(70C)	109.5
C(72)-C(71)-S(10)	120.6(11)
C(72)-C(71)-C(76)	116.3(13)
C(76)-C(71)-S(10)	122.9(11)
C(71)-C(72)-H(72)	119.1
C(73)-C(72)-C(71)	121.8(16)
C(73)-C(72)-H(72)	119.1
C(72)-C(73)-H(73)	118.4
C(72)-C(73)-C(74)	123.1(16)
C(74)-C(73)-H(73)	118.4
C(73)-C(74)-C(75)	116.1(15)
C(73)-C(74)-C(77)	122.4(16)
C(75)-C(74)-C(77)	121.1(17)
C(74)-C(75)-H(75)	119.6
C(76)-C(75)-C(74)	120.8(16)
C(76)-C(75)-H(75)	119.6
C(71)-C(76)-H(76)	119.4
C(75)-C(76)-C(71)	121.3(15)
C(75)-C(76)-H(76)	119.4
C(74)-C(77)-C(78)	115.7(18)
C(74)-C(77)-C(79)	109.2(17)
C(78)-C(77)-C(79)	97.9(17)
C(80)-C(77)-C(74)	107.4(17)

C(80)-C(77)-C(78)	115.5(19)
C(80)-C(77)-C(79)	111(2)
C(77)-C(78)-H(78A)	109.5
C(77)-C(78)-H(78B)	109.5
C(77)-C(78)-H(78C)	109.5
H(78A)-C(78)-H(78B)	109.5
H(78A)-C(78)-H(78C)	109.5
H(78B)-C(78)-H(78C)	109.5
C(77)-C(79)-H(79A)	109.5
C(77)-C(79)-H(79B)	109.5
C(77)-C(79)-H(79C)	109.5
H(79A)-C(79)-H(79B)	109.5
H(79A)-C(79)-H(79C)	109.5
H(79B)-C(79)-H(79C)	109.5
C(77)-C(80)-H(80A)	109.5
C(77)-C(80)-H(80B)	109.5
C(77)-C(80)-H(80C)	109.5
H(80A)-C(80)-H(80B)	109.5
H(80A)-C(80)-H(80C)	109.5
H(80B)-C(80)-H(80C)	109.5
C(82)-C(81)-S(13)	115.2(12)
C(86)-C(81)-S(13)	123.9(13)
C(86)-C(81)-C(82)	120.8(14)
C(81)-C(82)-H(82)	119.7
C(83)-C(82)-C(81)	120.6(17)
C(83)-C(82)-H(82)	119.7
C(82)-C(83)-H(83)	120.5
C(82)-C(83)-C(84)	118.9(17)
C(84)-C(83)-H(83)	120.5
C(83)-C(84)-C(87)	117.1(17)
C(85)-C(84)-C(83)	120.5(15)
C(85)-C(84)-C(87)	122.4(17)
C(84)-C(85)-H(85)	119.5
C(84)-C(85)-C(86)	121.0(16)
C(86)-C(85)-H(85)	119.5
C(81)-C(86)-C(85)	118.0(16)
C(81)-C(86)-H(86)	121.0
C(85)-C(86)-H(86)	121.0

C(88)-C(87)-C(84)	111.2(18)
C(88)-C(87)-C(89)	108.8(19)
C(88)-C(87)-C(90)	109.2(19)
C(89)-C(87)-C(84)	107.0(17)
C(89)-C(87)-C(90)	113(2)
C(90)-C(87)-C(84)	107.3(18)
C(87)-C(88)-H(88A)	109.5
C(87)-C(88)-H(88B)	109.5
C(87)-C(88)-H(88C)	109.5
H(88A)-C(88)-H(88B)	109.5
H(88A)-C(88)-H(88C)	109.5
H(88B)-C(88)-H(88C)	109.5
C(87)-C(89)-H(89A)	109.5
C(87)-C(89)-H(89B)	109.5
C(87)-C(89)-H(89C)	109.5
H(89A)-C(89)-H(89B)	109.5
H(89A)-C(89)-H(89C)	109.5
H(89B)-C(89)-H(89C)	109.5
C(87)-C(90)-H(90A)	109.5
C(87)-C(90)-H(90B)	109.5
C(87)-C(90)-H(90C)	109.5
H(90A)-C(90)-H(90B)	109.5
H(90A)-C(90)-H(90C)	109.5
H(90B)-C(90)-H(90C)	109.5
C(92)-C(91)-S(14)	121.9(13)
C(96)-C(91)-S(14)	118.1(11)
C(96)-C(91)-C(92)	119.7(15)
C(91)-C(92)-H(92)	121.5
C(91)-C(92)-C(93)	117.0(17)
C(93)-C(92)-H(92)	121.5
C(92)-C(93)-H(93)	117.4
C(94)-C(93)-C(92)	125.2(16)
C(94)-C(93)-H(93)	117.4
C(93)-C(94)-C(95)	117.1(16)
C(93)-C(94)-C(97)	124.5(16)
C(95)-C(94)-C(97)	118.4(16)
C(94)-C(95)-H(95)	120.6
C(94)-C(95)-C(96)	118.7(17)

C(96)-C(95)-H(95)	120.6
C(91)-C(96)-C(95)	122.3(15)
C(91)-C(96)-H(96)	118.9
C(95)-C(96)-H(96)	118.9
C(94)-C(97)-C(100)	113.2(17)
C(98)-C(97)-C(94)	110.9(17)
C(98)-C(97)-C(99)	111(2)
C(98)-C(97)-C(100)	105.9(17)
C(99)-C(97)-C(94)	111.0(17)
C(99)-C(97)-C(100)	105.0(18)
C(97)-C(98)-H(98A)	109.5
C(97)-C(98)-H(98B)	109.5
C(97)-C(98)-H(98C)	109.5
H(98A)-C(98)-H(98B)	109.5
H(98A)-C(98)-H(98C)	109.5
H(98B)-C(98)-H(98C)	109.5
C(97)-C(99)-H(99A)	109.5
C(97)-C(99)-H(99B)	109.5
C(97)-C(99)-H(99C)	109.5
H(99A)-C(99)-H(99B)	109.5
H(99A)-C(99)-H(99C)	109.5
H(99B)-C(99)-H(99C)	109.5
C(97)-C(100)-H(10D)	109.5
C(97)-C(100)-H(10E)	109.5
C(97)-C(100)-H(10F)	109.5
H(10D)-C(100)-H(10E)	109.5
H(10D)-C(100)-H(10F)	109.5
H(10E)-C(100)-H(10F)	109.5
C(102)-C(101)-S(16)	120.4(12)
C(102)-C(101)-C(106)	122.3(14)
C(106)-C(101)-S(16)	117.2(11)
C(101)-C(102)-H(102)	122.2
C(101)-C(102)-C(103)	115.5(17)
C(103)-C(102)-H(102)	122.2
C(102)-C(103)-H(103)	118.0
C(104)-C(103)-C(102)	124.0(18)
C(104)-C(103)-H(103)	118.0
C(103)-C(104)-C(105)	115.2(16)

C(103)-C(104)-C(107)	119.7(15)
C(105)-C(104)-C(107)	125.0(16)
C(104)-C(105)-H(105)	117.6
C(106)-C(105)-C(104)	124.8(17)
C(106)-C(105)-H(105)	117.6
C(101)-C(106)-H(106)	121.0
C(105)-C(106)-C(101)	118.1(16)
C(105)-C(106)-H(106)	121.0
C(104)-C(107)-C(108)	108.8(13)
C(104)-C(107)-C(109)	111.1(14)
C(109)-C(107)-C(108)	103.4(16)
C(110)-C(107)-C(104)	109.8(16)
C(110)-C(107)-C(108)	112.3(15)
C(110)-C(107)-C(109)	111.4(15)
C(107)-C(108)-H(10G)	109.5
C(107)-C(108)-H(10H)	109.5
C(107)-C(108)-H(10I)	109.5
H(10G)-C(108)-H(10H)	109.5
H(10G)-C(108)-H(10I)	109.5
H(10H)-C(108)-H(10I)	109.5
C(107)-C(109)-H(10J)	109.5
C(107)-C(109)-H(10K)	109.5
C(107)-C(109)-H(10L)	109.5
H(10J)-C(109)-H(10K)	109.5
H(10J)-C(109)-H(10L)	109.5
H(10K)-C(109)-H(10L)	109.5
C(107)-C(110)-H(11A)	109.5
C(107)-C(110)-H(11B)	109.5
C(107)-C(110)-H(11C)	109.5
H(11A)-C(110)-H(11B)	109.5
H(11A)-C(110)-H(11C)	109.5
H(11B)-C(110)-H(11C)	109.5
C(112)-C(111)-S(15)	121.0(15)
C(116)-C(111)-S(15)	114.6(15)
C(116)-C(111)-C(112)	124(2)
C(111)-C(112)-H(112)	121.9
C(113)-C(112)-C(111)	116(2)
C(113)-C(112)-H(112)	121.9

C(112)-C(113)-H(113)	117.1
C(112)-C(113)-C(114)	126(3)
C(114)-C(113)-H(113)	117.1
C(113)-C(114)-C(117)	124(3)
C(115)-C(114)-C(113)	116(3)
C(115)-C(114)-C(117)	119(3)
C(114)-C(115)-H(115)	118.3
C(114)-C(115)-C(116)	123(2)
C(116)-C(115)-H(115)	118.3
C(111)-C(116)-C(115)	113.9(19)
C(111)-C(116)-H(116)	123.1
C(115)-C(116)-H(116)	123.1
C(114)-C(117)-C(119)	111(3)
C(114)-C(117)-C(120)	107(3)
C(118)-C(117)-C(114)	118(3)
C(118)-C(117)-C(119)	115(3)
C(118)-C(117)-C(120)	106(3)
C(120)-C(117)-C(119)	97(3)
C(117)-C(118)-H(11D)	109.5
C(117)-C(118)-H(11E)	109.5
C(117)-C(118)-H(11F)	109.5
H(11D)-C(118)-H(11E)	109.5
H(11D)-C(118)-H(11F)	109.5
H(11E)-C(118)-H(11F)	109.5
C(117)-C(119)-H(11G)	109.5
C(117)-C(119)-H(11H)	109.5
C(117)-C(119)-H(11I)	109.5
H(11G)-C(119)-H(11H)	109.5
H(11G)-C(119)-H(11I)	109.5
H(11H)-C(119)-H(11I)	109.5
C(117)-C(120)-H(12A)	109.5
C(117)-C(120)-H(12B)	109.5
C(117)-C(120)-H(12C)	109.5
H(12A)-C(120)-H(12B)	109.5
H(12A)-C(120)-H(12C)	109.5
H(12B)-C(120)-H(12C)	109.5
C(122)-C(121)-S(12)	124.6(14)
C(122)-C(121)-C(126)	119.9(15)

C(126)-C(121)-S(12)	115.4(13)
C(121)-C(122)-H(122)	118.8
C(121)-C(122)-C(123)	122.5(16)
C(123)-C(122)-H(122)	118.8
C(122)-C(123)-H(123)	120.4
C(122)-C(123)-C(124)	119.2(16)
C(124)-C(123)-H(123)	120.4
C(123)-C(124)-C(127)	123.4(16)
C(125)-C(124)-C(123)	117.4(16)
C(125)-C(124)-C(127)	118.5(17)
C(124)-C(125)-H(125)	118.2
C(124)-C(125)-C(126)	123.7(18)
C(126)-C(125)-H(125)	118.2
C(121)-C(126)-H(126)	121.4
C(125)-C(126)-C(121)	117.3(17)
C(125)-C(126)-H(126)	121.4
C(124)-C(127)-C(128)	109.0(16)
C(129)-C(127)-C(124)	109.7(15)
C(129)-C(127)-C(128)	108.2(16)
C(130)-C(127)-C(124)	108.5(14)
C(130)-C(127)-C(128)	110.5(17)
C(130)-C(127)-C(129)	110.9(18)
C(127)-C(128)-H(12D)	109.5
C(127)-C(128)-H(12E)	109.5
C(127)-C(128)-H(12F)	109.5
H(12D)-C(128)-H(12E)	109.5
H(12D)-C(128)-H(12F)	109.5
H(12E)-C(128)-H(12F)	109.5
C(127)-C(129)-H(12G)	109.5
C(127)-C(129)-H(12H)	109.5
C(127)-C(129)-H(12I)	109.5
H(12G)-C(129)-H(12H)	109.5
H(12G)-C(129)-H(12I)	109.5
H(12H)-C(129)-H(12I)	109.5
C(127)-C(130)-H(13A)	109.5
C(127)-C(130)-H(13B)	109.5
C(127)-C(130)-H(13C)	109.5
H(13A)-C(130)-H(13B)	109.5

H(13A)-C(130)-H(13C)	109.5
H(13B)-C(130)-H(13C)	109.5
C(132)-C(131)-S(19)	116.1(11)
C(136)-C(131)-S(19)	123.5(12)
C(136)-C(131)-C(132)	120.3(14)
C(131)-C(132)-H(132)	121.0
C(131)-C(132)-C(133)	118.1(16)
C(133)-C(132)-H(132)	121.0
C(132)-C(133)-H(133)	119.4
C(134)-C(133)-C(132)	121.2(17)
C(134)-C(133)-H(133)	119.4
C(133)-C(134)-C(135)	120.0(17)
C(133)-C(134)-C(137)	120.3(17)
C(135)-C(134)-C(137)	119.6(16)
C(134)-C(135)-H(135)	120.5
C(136)-C(135)-C(134)	118.9(16)
C(136)-C(135)-H(135)	120.5
C(131)-C(136)-C(135)	121.3(16)
C(131)-C(136)-H(136)	119.3
C(135)-C(136)-H(136)	119.3
C(138)-C(137)-C(134)	110.8(14)
C(139)-C(137)-C(134)	111.4(17)
C(139)-C(137)-C(138)	108.9(18)
C(139)-C(137)-C(140)	111.7(18)
C(140)-C(137)-C(134)	106.2(17)
C(140)-C(137)-C(138)	107.8(19)
C(137)-C(138)-H(13D)	109.5
C(137)-C(138)-H(13E)	109.5
C(137)-C(138)-H(13F)	109.5
H(13D)-C(138)-H(13E)	109.5
H(13D)-C(138)-H(13F)	109.5
H(13E)-C(138)-H(13F)	109.5
C(137)-C(139)-H(13G)	109.5
C(137)-C(139)-H(13H)	109.5
C(137)-C(139)-H(13I)	109.5
H(13G)-C(139)-H(13H)	109.5
H(13G)-C(139)-H(13I)	109.5
H(13H)-C(139)-H(13I)	109.5

C(137)-C(140)-H(14A)	109.5
C(137)-C(140)-H(14B)	109.5
C(137)-C(140)-H(14C)	109.5
H(14A)-C(140)-H(14B)	109.5
H(14A)-C(140)-H(14C)	109.5
H(14B)-C(140)-H(14C)	109.5
C(142)-C(141)-S(18)	118.9(13)
C(146)-C(141)-S(18)	122.4(13)
C(146)-C(141)-C(142)	118.7(16)
C(141)-C(142)-H(142)	121.3
C(141)-C(142)-C(143)	117.4(16)
C(143)-C(142)-H(142)	121.3
C(142)-C(143)-H(143)	119.9
C(144)-C(143)-C(142)	120.3(16)
C(144)-C(143)-H(143)	119.9
C(143)-C(144)-C(145)	121(2)
C(143)-C(144)-C(147)	119.4(16)
C(145)-C(144)-C(147)	119.6(19)
C(144)-C(145)-H(145)	119.7
C(144)-C(145)-C(146)	121(2)
C(146)-C(145)-H(145)	119.7
C(141)-C(146)-C(145)	122.0(19)
C(141)-C(146)-H(146)	119.0
C(145)-C(146)-H(146)	119.0
C(148)-C(147)-C(144)	110.4(16)
C(148)-C(147)-C(149)	106.6(17)
C(148)-C(147)-C(150)	111.8(19)
C(149)-C(147)-C(144)	105.8(17)
C(150)-C(147)-C(144)	108.1(17)
C(150)-C(147)-C(149)	113.9(18)
C(147)-C(148)-H(14D)	109.5
C(147)-C(148)-H(14E)	109.5
C(147)-C(148)-H(14F)	109.5
H(14D)-C(148)-H(14E)	109.5
H(14D)-C(148)-H(14F)	109.5
H(14E)-C(148)-H(14F)	109.5
C(147)-C(149)-H(14G)	109.5
C(147)-C(149)-H(14H)	109.5

C(147)-C(149)-H(14I)	109.5
H(14G)-C(149)-H(14H)	109.5
H(14G)-C(149)-H(14I)	109.5
H(14H)-C(149)-H(14I)	109.5
C(147)-C(150)-H(15A)	109.5
C(147)-C(150)-H(15B)	109.5
C(147)-C(150)-H(15C)	109.5
H(15A)-C(150)-H(15B)	109.5
H(15A)-C(150)-H(15C)	109.5
H(15B)-C(150)-H(15C)	109.5
C(152)-C(151)-S(17)	119.1(11)
C(152)-C(151)-C(156)	118.7(15)
C(156)-C(151)-S(17)	122.2(11)
C(151)-C(152)-H(152)	119.2
C(153)-C(152)-C(151)	121.5(15)
C(153)-C(152)-H(152)	119.2
C(152)-C(153)-H(153)	119.0
C(152)-C(153)-C(154)	122.1(17)
C(154)-C(153)-H(153)	119.0
C(153)-C(154)-C(155)	116.1(17)
C(153)-C(154)-C(157)	123.6(17)
C(155)-C(154)-C(157)	120.2(18)
C(154)-C(155)-H(155)	119.8
C(156)-C(155)-C(154)	120.5(17)
C(156)-C(155)-H(155)	119.8
C(151)-C(156)-C(155)	120.9(15)
C(151)-C(156)-H(156)	119.6
C(155)-C(156)-H(156)	119.6
C(154)-C(157)-C(158)	105.4(18)
C(159)-C(157)-C(154)	113.4(19)
C(159)-C(157)-C(158)	107(2)
C(159)-C(157)-C(160)	110(2)
C(160)-C(157)-C(154)	110(2)
C(160)-C(157)-C(158)	112(2)
C(157)-C(158)-H(15D)	109.5
C(157)-C(158)-H(15E)	109.5
C(157)-C(158)-H(15F)	109.5
H(15D)-C(158)-H(15E)	109.5

H(15D)-C(158)-H(15F)	109.5
H(15E)-C(158)-H(15F)	109.5
C(157)-C(159)-H(15G)	109.5
C(157)-C(159)-H(15H)	109.5
C(157)-C(159)-H(15I)	109.5
H(15G)-C(159)-H(15H)	109.5
H(15G)-C(159)-H(15I)	109.5
H(15H)-C(159)-H(15I)	109.5
C(157)-C(160)-H(16A)	109.5
C(157)-C(160)-H(16B)	109.5
C(157)-C(160)-H(16C)	109.5
H(16A)-C(160)-H(16B)	109.5
H(16A)-C(160)-H(16C)	109.5
H(16B)-C(160)-H(16C)	109.5
C(162)-C(161)-S(8)	122.6(11)
C(166)-C(161)-S(8)	118.2(11)
C(166)-C(161)-C(162)	119.2(13)
C(161)-C(162)-H(162)	120.6
C(161)-C(162)-C(163)	118.8(15)
C(163)-C(162)-H(162)	120.6
C(162)-C(163)-H(163)	118.8
C(164)-C(163)-C(162)	122.5(16)
C(164)-C(163)-H(163)	118.8
C(163)-C(164)-C(165)	117.6(15)
C(163)-C(164)-C(167)	120.2(15)
C(165)-C(164)-C(167)	122.0(15)
C(164)-C(165)-H(165)	120.6
C(166)-C(165)-C(164)	118.9(16)
C(166)-C(165)-H(165)	120.6
C(161)-C(166)-C(165)	122.9(14)
C(161)-C(166)-H(166)	118.6
C(165)-C(166)-H(166)	118.6
C(164)-C(167)-C(168)	106.0(15)
C(164)-C(167)-C(169)	108.9(15)
C(168)-C(167)-C(169)	111.8(16)
C(170)-C(167)-C(164)	112.8(14)
C(170)-C(167)-C(168)	109.2(17)
C(170)-C(167)-C(169)	108.2(16)

C(167)-C(168)-H(16D)	109.5
C(167)-C(168)-H(16E)	109.5
C(167)-C(168)-H(16F)	109.5
H(16D)-C(168)-H(16E)	109.5
H(16D)-C(168)-H(16F)	109.5
H(16E)-C(168)-H(16F)	109.5
C(167)-C(169)-H(16G)	109.5
C(167)-C(169)-H(16H)	109.5
C(167)-C(169)-H(16I)	109.5
H(16G)-C(169)-H(16H)	109.5
H(16G)-C(169)-H(16I)	109.5
H(16H)-C(169)-H(16I)	109.5
C(167)-C(170)-H(17A)	109.5
C(167)-C(170)-H(17B)	109.5
C(167)-C(170)-H(17C)	109.5
H(17A)-C(170)-H(17B)	109.5
H(17A)-C(170)-H(17C)	109.5
H(17B)-C(170)-H(17C)	109.5
C(172)-C(171)-S(20)	116.2(11)
C(172)-C(171)-C(176)	120.6(15)
C(176)-C(171)-S(20)	122.9(13)
C(171)-C(172)-H(172)	120.6
C(171)-C(172)-C(173)	118.8(15)
C(173)-C(172)-H(172)	120.6
C(172)-C(173)-H(173)	118.9
C(172)-C(173)-C(174)	122.3(17)
C(174)-C(173)-H(173)	118.9
C(173)-C(174)-C(177)	123.8(16)
C(175)-C(174)-C(173)	115.9(17)
C(175)-C(174)-C(177)	120.1(16)
C(174)-C(175)-H(175)	117.7
C(174)-C(175)-C(176)	124.5(16)
C(176)-C(175)-H(175)	117.7
C(171)-C(176)-C(175)	117.8(15)
C(171)-C(176)-H(176)	121.1
C(175)-C(176)-H(176)	121.1
C(178)-C(177)-C(174)	116.4(17)
C(178)-C(177)-C(179)	105.4(19)

C(178)-C(177)-C(180)	114(2)
C(179)-C(177)-C(174)	105.9(18)
C(180)-C(177)-C(174)	107.5(17)
C(180)-C(177)-C(179)	107(2)
C(177)-C(178)-H(17D)	109.5
C(177)-C(178)-H(17E)	109.5
C(177)-C(178)-H(17F)	109.5
H(17D)-C(178)-H(17E)	109.5
H(17D)-C(178)-H(17F)	109.5
H(17E)-C(178)-H(17F)	109.5
C(177)-C(179)-H(17G)	109.5
C(177)-C(179)-H(17H)	109.5
C(177)-C(179)-H(17I)	109.5
H(17G)-C(179)-H(17H)	109.5
H(17G)-C(179)-H(17I)	109.5
H(17H)-C(179)-H(17I)	109.5
C(177)-C(180)-H(18D)	109.5
C(177)-C(180)-H(18E)	109.5
C(177)-C(180)-H(18F)	109.5
H(18D)-C(180)-H(18E)	109.5
H(18D)-C(180)-H(18F)	109.5
H(18E)-C(180)-H(18F)	109.5
C(182)-C(181)-S(22)	119.6(13)
C(182)-C(181)-C(186)	116.6(14)
C(186)-C(181)-S(22)	123.8(12)
C(181)-C(182)-H(182)	119.0
C(181)-C(182)-C(183)	122.0(19)
C(183)-C(182)-H(182)	119.0
C(182)-C(183)-H(183)	119.6
C(184)-C(183)-C(182)	120.8(19)
C(184)-C(183)-H(183)	119.6
C(183)-C(184)-C(185)	119.0(16)
C(183)-C(184)-C(187)	124.4(17)
C(185)-C(184)-C(187)	116.2(16)
C(184)-C(185)-H(185)	120.8
C(186)-C(185)-C(184)	118.3(17)
C(186)-C(185)-H(185)	120.8
C(181)-C(186)-H(186)	118.5

C(185)-C(186)-C(181)	123.0(17)
C(185)-C(186)-H(186)	118.5
C(184)-C(187)-C(190)	109.4(17)
C(188)-C(187)-C(184)	111.4(15)
C(188)-C(187)-C(189)	108.3(18)
C(188)-C(187)-C(190)	107.2(16)
C(189)-C(187)-C(184)	112.4(16)
C(189)-C(187)-C(190)	108.0(16)
C(187)-C(188)-H(18G)	109.5
C(187)-C(188)-H(18H)	109.5
C(187)-C(188)-H(18I)	109.5
H(18G)-C(188)-H(18H)	109.5
H(18G)-C(188)-H(18I)	109.5
H(18H)-C(188)-H(18I)	109.5
C(187)-C(189)-H(18J)	109.5
C(187)-C(189)-H(18K)	109.5
C(187)-C(189)-H(18L)	109.5
H(18J)-C(189)-H(18K)	109.5
H(18J)-C(189)-H(18L)	109.5
H(18K)-C(189)-H(18L)	109.5
C(187)-C(190)-H(19D)	109.5
C(187)-C(190)-H(19E)	109.5
C(187)-C(190)-H(19F)	109.5
H(19D)-C(190)-H(19E)	109.5
H(19D)-C(190)-H(19F)	109.5
H(19E)-C(190)-H(19F)	109.5
C(192)-C(191)-S(21)	121.3(12)
C(196)-C(191)-S(21)	121.8(12)
C(196)-C(191)-C(192)	116.8(14)
C(191)-C(192)-H(192)	119.4
C(191)-C(192)-C(193)	121.3(16)
C(193)-C(192)-H(192)	119.4
C(192)-C(193)-H(193)	118.6
C(194)-C(193)-C(192)	122.8(18)
C(194)-C(193)-H(193)	118.6
C(193)-C(194)-C(195)	115.8(18)
C(193)-C(194)-C(197)	121.9(19)
C(195)-C(194)-C(197)	122.2(18)

C(194)-C(195)-H(195)	119.0
C(194)-C(195)-C(196)	122.0(17)
C(196)-C(195)-H(195)	119.0
C(191)-C(196)-C(195)	121.2(16)
C(191)-C(196)-H(196)	119.4
C(195)-C(196)-H(196)	119.4
C(198)-C(197)-C(194)	109.1(18)
C(198)-C(197)-C(200)	109(2)
C(199)-C(197)-C(194)	111.2(19)
C(199)-C(197)-C(198)	103(2)
C(199)-C(197)-C(200)	112(2)
C(200)-C(197)-C(194)	112(2)
C(197)-C(198)-H(19G)	109.5
C(197)-C(198)-H(19H)	109.5
C(197)-C(198)-H(19I)	109.5
H(19G)-C(198)-H(19H)	109.5
H(19G)-C(198)-H(19I)	109.5
H(19H)-C(198)-H(19I)	109.5
C(197)-C(199)-H(19J)	109.5
C(197)-C(199)-H(19K)	109.5
C(197)-C(199)-H(19L)	109.5
H(19J)-C(199)-H(19K)	109.5
H(19J)-C(199)-H(19L)	109.5
H(19K)-C(199)-H(19L)	109.5
C(197)-C(200)-H(20D)	109.5
C(197)-C(200)-H(20E)	109.5
C(197)-C(200)-H(20F)	109.5
H(20D)-C(200)-H(20E)	109.5
H(20D)-C(200)-H(20F)	109.5
H(20E)-C(200)-H(20F)	109.5
C(202)-C(201)-S(23)	117.5(11)
C(206)-C(201)-S(23)	124.6(11)
C(206)-C(201)-C(202)	117.9(13)
C(201)-C(202)-H(202)	119.1
C(201)-C(202)-C(203)	121.9(15)
C(203)-C(202)-H(202)	119.1
C(202)-C(203)-H(203)	120.0
C(202)-C(203)-C(204)	120.1(15)

C(204)-C(203)-H(203)	120.0
C(203)-C(204)-C(207)	123.0(14)
C(205)-C(204)-C(203)	118.0(13)
C(205)-C(204)-C(207)	119.0(13)
C(204)-C(205)-H(205)	119.6
C(204)-C(205)-C(206)	120.8(14)
C(206)-C(205)-H(205)	119.6
C(201)-C(206)-C(205)	121.4(15)
C(201)-C(206)-H(206)	119.3
C(205)-C(206)-H(206)	119.3
C(208)-C(207)-C(204)	107.7(14)
C(208)-C(207)-C(209)	111.3(15)
C(208)-C(207)-C(210)	108.7(14)
C(209)-C(207)-C(204)	107.7(13)
C(209)-C(207)-C(210)	110.5(15)
C(210)-C(207)-C(204)	111.1(13)
C(207)-C(208)-H(20G)	109.5
C(207)-C(208)-H(20H)	109.5
C(207)-C(208)-H(20I)	109.5
H(20G)-C(208)-H(20H)	109.5
H(20G)-C(208)-H(20I)	109.5
H(20H)-C(208)-H(20I)	109.5
C(207)-C(209)-H(20J)	109.5
C(207)-C(209)-H(20K)	109.5
C(207)-C(209)-H(20L)	109.5
H(20J)-C(209)-H(20K)	109.5
H(20J)-C(209)-H(20L)	109.5
H(20K)-C(209)-H(20L)	109.5
C(207)-C(210)-H(21A)	109.5
C(207)-C(210)-H(21B)	109.5
C(207)-C(210)-H(21C)	109.5
H(21A)-C(210)-H(21B)	109.5
H(21A)-C(210)-H(21C)	109.5
H(21B)-C(210)-H(21C)	109.5
C(212)-C(211)-S(26)	123.5(12)
C(216)-C(211)-S(26)	117.1(12)
C(216)-C(211)-C(212)	119.3(15)
C(211)-C(212)-H(212)	118.6

C(213)-C(212)-C(211)	122.8(15)
C(213)-C(212)-H(212)	118.6
C(212)-C(213)-H(213)	119.9
C(212)-C(213)-C(214)	120.1(16)
C(214)-C(213)-H(213)	119.9
C(213)-C(214)-C(215)	117.0(17)
C(213)-C(214)-C(217)	118.3(16)
C(215)-C(214)-C(217)	124.7(17)
C(214)-C(215)-H(215)	118.9
C(214)-C(215)-C(216)	122.1(17)
C(216)-C(215)-H(215)	118.9
C(211)-C(216)-C(215)	118.4(16)
C(211)-C(216)-H(216)	120.8
C(215)-C(216)-H(216)	120.8
C(218)-C(217)-C(214)	111(2)
C(218)-C(217)-C(219)	108(2)
C(218)-C(217)-C(220)	112(2)
C(219)-C(217)-C(214)	109.0(18)
C(220)-C(217)-C(214)	109.3(19)
C(220)-C(217)-C(219)	107(2)
C(217)-C(218)-H(21D)	109.5
C(217)-C(218)-H(21E)	109.5
C(217)-C(218)-H(21F)	109.5
H(21D)-C(218)-H(21E)	109.5
H(21D)-C(218)-H(21F)	109.5
H(21E)-C(218)-H(21F)	109.5
C(217)-C(219)-H(21G)	109.5
C(217)-C(219)-H(21H)	109.5
C(217)-C(219)-H(21I)	109.5
H(21G)-C(219)-H(21H)	109.5
H(21G)-C(219)-H(21I)	109.5
H(21H)-C(219)-H(21I)	109.5
C(217)-C(220)-H(22A)	109.5
C(217)-C(220)-H(22B)	109.5
C(217)-C(220)-H(22C)	109.5
H(22A)-C(220)-H(22B)	109.5
H(22A)-C(220)-H(22C)	109.5
H(22B)-C(220)-H(22C)	109.5

C(222)-C(221)-S(25)	121.8(15)
C(226)-C(221)-S(25)	120.0(12)
C(226)-C(221)-C(222)	118.2(17)
C(221)-C(222)-H(222)	120.4
C(223)-C(222)-C(221)	119(2)
C(223)-C(222)-H(222)	120.4
C(222)-C(223)-H(223)	118.8
C(224)-C(223)-C(222)	122(2)
C(224)-C(223)-H(223)	118.8
C(223)-C(224)-C(225)	121(2)
C(223)-C(224)-C(227)	119(2)
C(225)-C(224)-C(227)	120(2)
C(224)-C(225)-H(225)	122.0
C(226)-C(225)-C(224)	116(2)
C(226)-C(225)-H(225)	122.0
C(221)-C(226)-C(225)	123.5(18)
C(221)-C(226)-H(226)	118.3
C(225)-C(226)-H(226)	118.3
C(228)-C(227)-C(224)	117(2)
C(228)-C(227)-C(229)	104(2)
C(228)-C(227)-C(260)	109(2)
C(229)-C(227)-C(224)	113(2)
C(260)-C(227)-C(224)	109(2)
C(260)-C(227)-C(229)	105(2)
C(227)-C(228)-H(22D)	109.5
C(227)-C(228)-H(22E)	109.5
C(227)-C(228)-H(22F)	109.5
H(22D)-C(228)-H(22E)	109.5
H(22D)-C(228)-H(22F)	109.5
H(22E)-C(228)-H(22F)	109.5
C(227)-C(229)-H(22G)	109.5
C(227)-C(229)-H(22H)	109.5
C(227)-C(229)-H(22I)	109.5
H(22G)-C(229)-H(22H)	109.5
H(22G)-C(229)-H(22I)	109.5
H(22H)-C(229)-H(22I)	109.5
C(231)-C(230)-S(24)	119.2(11)
C(235)-C(230)-S(24)	123.5(11)

C(235)-C(230)-C(231)	116.1(14)
C(230)-C(231)-H(231)	118.9
C(232)-C(231)-C(230)	122.3(15)
C(232)-C(231)-H(231)	118.9
C(231)-C(232)-H(232)	119.0
C(231)-C(232)-C(233)	122.0(17)
C(233)-C(232)-H(232)	119.0
C(232)-C(233)-C(234)	116.7(16)
C(232)-C(233)-C(236)	123.0(16)
C(234)-C(233)-C(236)	120.3(15)
C(233)-C(234)-H(234)	119.9
C(235)-C(234)-C(233)	120.2(16)
C(235)-C(234)-H(234)	119.9
C(230)-C(235)-H(235)	118.8
C(234)-C(235)-C(230)	122.3(15)
C(234)-C(235)-H(235)	118.8
C(233)-C(236)-C(237)	106.7(19)
C(238)-C(236)-C(233)	112.2(19)
C(238)-C(236)-C(237)	106.4(19)
C(238)-C(236)-C(239)	112(2)
C(239)-C(236)-C(233)	112.3(18)
C(239)-C(236)-C(237)	107(2)
C(236)-C(237)-H(23A)	109.5
C(236)-C(237)-H(23B)	109.5
C(236)-C(237)-H(23C)	109.5
H(23A)-C(237)-H(23B)	109.5
H(23A)-C(237)-H(23C)	109.5
H(23B)-C(237)-H(23C)	109.5
C(236)-C(238)-H(23D)	109.5
C(236)-C(238)-H(23E)	109.5
C(236)-C(238)-H(23F)	109.5
H(23D)-C(238)-H(23E)	109.5
H(23D)-C(238)-H(23F)	109.5
H(23E)-C(238)-H(23F)	109.5
C(236)-C(239)-H(23G)	109.5
C(236)-C(239)-H(23H)	109.5
C(236)-C(239)-H(23I)	109.5
H(23G)-C(239)-H(23H)	109.5

H(23G)-C(239)-H(23I)	109.5
H(23H)-C(239)-H(23I)	109.5
C(241)-C(240)-S(2)	125.2(13)
C(241)-C(240)-C(245)	115.5(16)
C(245)-C(240)-S(2)	119.2(13)
C(240)-C(241)-H(241)	119.3
C(240)-C(241)-C(242)	121.5(16)
C(242)-C(241)-H(241)	119.3
C(241)-C(242)-H(242)	119.1
C(243)-C(242)-C(241)	121.8(17)
C(243)-C(242)-H(242)	119.1
C(242)-C(243)-C(244)	117.0(18)
C(242)-C(243)-C(246)	121.0(17)
C(244)-C(243)-C(246)	122.0(17)
C(243)-C(244)-H(244)	119.5
C(245)-C(244)-C(243)	121.0(18)
C(245)-C(244)-H(244)	119.5
C(240)-C(245)-H(245)	118.5
C(244)-C(245)-C(240)	123.1(17)
C(244)-C(245)-H(245)	118.5
C(243)-C(246)-C(247)	109(2)
C(243)-C(246)-C(248)	112.0(19)
C(247)-C(246)-C(248)	109(2)
C(249)-C(246)-C(243)	111(2)
C(249)-C(246)-C(247)	110(2)
C(249)-C(246)-C(248)	106(2)
C(246)-C(247)-H(24A)	109.5
C(246)-C(247)-H(24B)	109.5
C(246)-C(247)-H(24C)	109.5
H(24A)-C(247)-H(24B)	109.5
H(24A)-C(247)-H(24C)	109.5
H(24B)-C(247)-H(24C)	109.5
C(246)-C(248)-H(24D)	109.5
C(246)-C(248)-H(24E)	109.5
C(246)-C(248)-H(24F)	109.5
H(24D)-C(248)-H(24E)	109.5
H(24D)-C(248)-H(24F)	109.5
H(24E)-C(248)-H(24F)	109.5

C(246)-C(249)-H(24G)	109.5
C(246)-C(249)-H(24H)	109.5
C(246)-C(249)-H(24I)	109.5
H(24G)-C(249)-H(24H)	109.5
H(24G)-C(249)-H(24I)	109.5
H(24H)-C(249)-H(24I)	109.5
C(251)-C(250)-S(1)	122.8(13)
C(255)-C(250)-S(1)	119.3(14)
C(255)-C(250)-C(251)	117.7(15)
C(250)-C(251)-H(251)	120.2
C(252)-C(251)-C(250)	119.7(15)
C(252)-C(251)-H(251)	120.2
C(251)-C(252)-H(252)	118.8
C(251)-C(252)-C(253)	122.4(16)
C(253)-C(252)-H(252)	118.8
C(252)-C(253)-C(256)	123.2(16)
C(254)-C(253)-C(252)	116.6(15)
C(254)-C(253)-C(256)	120.0(17)
C(253)-C(254)-H(254)	118.4
C(253)-C(254)-C(255)	123.3(17)
C(255)-C(254)-H(254)	118.4
C(250)-C(255)-C(254)	120.3(17)
C(250)-C(255)-H(255)	119.9
C(254)-C(255)-H(255)	119.9
C(257)-C(256)-C(253)	108.9(17)
C(258)-C(256)-C(253)	113(2)
C(258)-C(256)-C(257)	110(2)
C(258)-C(256)-C(259)	114(2)
C(259)-C(256)-C(253)	110.0(17)
C(259)-C(256)-C(257)	99.7(19)
C(256)-C(257)-H(25A)	109.5
C(256)-C(257)-H(25B)	109.5
C(256)-C(257)-H(25C)	109.5
H(25A)-C(257)-H(25B)	109.5
H(25A)-C(257)-H(25C)	109.5
H(25B)-C(257)-H(25C)	109.5
C(256)-C(258)-H(25D)	109.5
C(256)-C(258)-H(25E)	109.5

C(256)-C(258)-H(25F)	109.5
H(25D)-C(258)-H(25E)	109.5
H(25D)-C(258)-H(25F)	109.5
H(25E)-C(258)-H(25F)	109.5
C(256)-C(259)-H(25G)	109.5
C(256)-C(259)-H(25H)	109.5
C(256)-C(259)-H(25I)	109.5
H(25G)-C(259)-H(25H)	109.5
H(25G)-C(259)-H(25I)	109.5
H(25H)-C(259)-H(25I)	109.5
C(227)-C(260)-H(26A)	109.5
C(227)-C(260)-H(26B)	109.5
C(227)-C(260)-H(26C)	109.5
H(26A)-C(260)-H(26B)	109.5
H(26A)-C(260)-H(26C)	109.5
H(26B)-C(260)-H(26C)	109.5

Symmetry transformations used to generate equivalent atoms:

Table 4. Anisotropic displacement parameters ($Å^2$x 10^3) for $Au_{42}(TBBT)_{26}$. The anisotropic displacement factor exponent takes the form: $-2\pi^2[h^2 a^{*2}U^{11} + ... + 2 h k a^* b^* U^{12}]$

	U^{11}	U^{22}	U^{33}	U^{23}	U^{13}	U^{12}
Au(1)	13	22	10	-2	-1	-3
Au(2)	16	28	15	-6	-1	0
Au(3)	15	19	12	-4	-2	-2
Au(4)	15	22	13	-4	-1	-4
Au(5)	18	27	11	-5	-1	-3
Au(6)	21	26	20	-1	-7	0
Au(7)	16	25	14	-5	0	-4
Au(8)	21	28	18	-9	-3	-4
Au(9)	22	25	21	-7	-8	-1
Au(10)	20	25	23	-9	-5	-2
Au(11)	19	26	13	-5	-3	1
Au(12)	18	23	14	-5	-6	-1
Au(13)	14	19	13	-4	-3	0
Au(14)	13	19	11	-3	-3	-3
Au(15)	16	24	14	-4	-5	-2
Au(16)	14	26	15	-5	-4	-2
Au(17)	14	26	14	-6	-1	0
Au(18)	22	33	17	-7	-5	3
Au(19)	20	31	19	-5	-5	-3
Au(20)	13	20	12	-4	-4	-1
Au(21)	14	20	12	-3	-2	-4
Au(22)	15	28	14	-4	-1	-1
Au(23)	20	24	17	-4	-4	-5
Au(24)	19	29	17	-7	-5	1
Au(25)	17	23	12	-3	-3	-2
Au(26)	21	28	17	-6	-3	0
Au(27)	21	28	21	-7	-8	1
Au(28)	22	23	22	-5	-9	0
Au(29)	24	22	20	-5	-9	3
Au(30)	21	23	16	-7	-3	-3
Au(31)	16	26	17	-7	-1	-2
Au(32)	16	22	17	-5	-5	-1
Au(33)	21	31	19	-9	-2	-2

Au(34)	22	27	20	-7	-4	-3
Au(35)	18	24	13	-7	-1	-2
Au(36)	22	27	14	-6	-1	-2
Au(37)	21	27	21	-3	-8	-3
Au(38)	24	27	24	-8	-6	2
Au(39)	20	28	24	-6	-1	-3
Au(40)	20	31	17	-5	-2	-2
Au(41)	33	34	19	-10	-3	-1
Au(42)	31	36	20	-4	-4	-2
S(1)	24	23	15	-9	-3	0
S(2)	26	29	26	-10	-5	-4
S(3)	23	28	16	-5	-2	2
S(4)	23	29	22	-6	-1	-2
S(5)	20	32	18	-6	-5	-3
S(6)	27	32	16	-8	-4	2
S(7)	19	32	19	-7	1	-7
S(8)	22	24	21	-5	-10	3
S(9)	20	35	22	-10	-7	2
S(10)	22	28	21	-10	-1	-7
S(11)	18	28	16	-5	2	-6
S(12)	23	23	18	-3	-2	-6
S(13)	19	34	11	-6	0	-4
S(14)	21	32	19	-10	-2	-3
S(15)	27	27	24	-8	-8	-5
S(16)	18	34	13	-1	-6	-6
S(17)	28	24	20	-5	-9	3
S(18)	26	40	24	-11	-4	2
S(19)	20	30	26	-10	-4	-4
S(20)	22	28	24	-15	-9	6
S(21)	19	35	14	-4	-2	0
S(22)	18	35	19	-10	1	-3
S(23)	17	32	21	-10	-7	-2
S(24)	22	33	23	-10	1	-5
S(25)	37	36	19	-9	-2	0
S(26)	26	31	27	-4	-6	1
C(1)	14	18	14	-5	-1	-2
C(2)	26	22	29	-11	-5	2
C(3)	41	43	42	-14	-7	4

C(4)	33	35	30	-12	-5	1
C(5)	32	36	31	-12	-5	-2
C(6)	32	33	28	-13	-6	-3
C(7)	51	50	50	-22	-7	1
C(8)	48	61	40	-15	-2	-2
C(9)	70	90	70	-31	-16	-14
C(10)	126	115	114	-49	-21	-2
C(11)	25	27	24	-12	-10	-5
C(12)	41	44	39	-16	-11	-1
C(13)	55	56	54	-21	-16	1
C(14)	55	57	54	-22	-11	1
C(15)	37	43	39	-12	-10	-2
C(16)	28	27	26	-4	-9	0
C(17)	78	78	79	-32	-17	1
C(18)	96	103	98	-34	-28	-5
C(19)	97	98	83	-29	-26	-14
C(20)	172	163	167	-64	-32	-5
C(21)	30	32	34	-12	-10	-2
C(22)	55	56	54	-19	-14	1
C(23)	70	70	68	-25	-18	-3
C(24)	141	143	143	-54	-32	-2
C(25)	57	60	60	-21	-15	1
C(26)	62	61	63	-21	-13	3
C(27)	154	155	156	-56	-32	-1
C(28)	169	169	175	-55	-32	0
C(29)	165	163	168	-56	-46	-7
C(30)	179	176	173	-61	-32	12
C(31)	22	20	21	-10	-5	0
C(32)	29	25	24	-7	-9	-3
C(33)	30	31	32	-8	-10	-1
C(34)	24	30	24	-9	-6	-3
C(35)	35	38	35	-12	-7	-4
C(36)	22	21	21	-4	-6	-4
C(37)	36	40	36	-12	-9	-1
C(38)	41	36	40	-19	-7	-7
C(39)	48	34	40	-9	-13	-5
C(40)	31	47	45	-14	-1	1
C(41)	24	17	18	-8	0	-2

C(42)	30	33	29	-12	-7	-1
C(43)	28	32	29	-9	-6	-3
C(44)	31	32	30	-10	-8	-4
C(45)	49	44	44	-16	-9	-4
C(46)	39	37	35	-14	-8	-2
C(47)	55	56	49	-20	-14	-1
C(48)	86	81	70	-36	-17	14
C(49)	92	105	86	-36	-25	4
C(50)	102	95	88	-27	-27	-4
C(51)	18	17	21	-6	-5	3
C(52)	34	38	31	-12	-11	-4
C(53)	32	34	31	-12	-7	-5
C(54)	22	25	27	-7	-3	1
C(55)	34	36	35	-12	-9	-2
C(56)	33	37	33	-15	-8	0
C(57)	44	45	45	-17	-9	2
C(58)	49	50	57	-19	-3	-10
C(59)	56	52	55	-26	-11	2
C(60)	59	57	68	-24	-21	8
C(61)	23	24	24	-7	-5	-2
C(62)	27	28	27	-9	-8	2
C(63)	29	28	26	-7	-5	-2
C(64)	31	31	27	-13	-8	-4
C(65)	23	26	25	-7	-4	-1
C(66)	29	30	29	-10	-6	0
C(67)	43	46	44	-16	-9	-2
C(68)	41	58	45	-9	-14	0
C(69)	38	54	50	-15	-18	4
C(70)	53	60	61	-19	-16	-9
C(71)	20	20	21	-8	-1	-1
C(72)	34	37	36	-14	-5	-1
C(73)	25	29	26	-11	0	3
C(74)	43	46	47	-19	-10	-1
C(75)	35	37	37	-13	-6	-1
C(76)	23	23	22	-6	0	1
C(77)	57	60	61	-22	-16	-2
C(78)	79	85	90	-16	-24	-3
C(79)	86	99	91	-32	-18	3

C(80)	96	101	109	-26	-30	-4
C(81)	21	24	16	-7	-4	2
C(82)	33	32	30	-14	-8	3
C(83)	38	37	36	-14	-10	-2
C(84)	28	30	28	-9	-11	0
C(85)	26	31	26	-9	-9	-1
C(86)	27	32	24	-7	-5	-1
C(87)	62	65	62	-22	-18	1
C(88)	57	54	57	-26	-26	-3
C(89)	62	71	74	-26	-24	6
C(90)	77	69	72	-20	-25	-2
C(91)	22	23	19	-10	-1	0
C(92)	33	34	31	-13	-9	1
C(93)	39	36	35	-13	-8	-1
C(94)	44	43	39	-15	-13	-1
C(95)	36	31	33	-17	-7	1
C(96)	29	28	31	-10	-4	-2
C(97)	57	53	53	-22	-12	-2
C(98)	87	89	92	-38	-21	-20
C(99)	119	113	112	-49	-22	-10
C(100)	97	99	87	-37	-29	-2
C(101)	18	18	19	-8	-5	2
C(102)	38	40	38	-13	-9	3
C(103)	43	47	46	-18	-10	1
C(104)	32	35	30	-10	-10	-3
C(105)	32	33	31	-11	-9	0
C(106)	34	35	36	-10	-8	0
C(107)	32	38	34	-11	-10	-1
C(108)	43	55	39	-7	-19	2
C(109)	34	55	49	-17	-15	15
C(110)	43	62	61	-21	-17	-4
C(111)	32	36	35	-9	-11	-3
C(112)	76	72	76	-25	-19	-1
C(113)	81	80	81	-30	-20	-4
C(114)	84	85	85	-33	-21	-3
C(115)	48	51	52	-20	-13	-3
C(116)	48	51	50	-17	-13	0
C(117)	99	101	102	-37	-23	-3

C(118)	124	130	128	-41	-36	-14
C(119)	123	128	122	-41	-30	-14
C(120)	128	122	131	-50	-21	0
C(121)	28	28	26	-11	-6	4
C(122)	24	23	21	-8	-4	0
C(123)	32	28	30	-10	-5	-3
C(124)	34	32	33	-11	-3	3
C(125)	38	38	37	-16	-8	-2
C(126)	33	34	31	-11	-5	2
C(127)	43	41	43	-15	-4	0
C(128)	61	72	66	-25	-1	-4
C(129)	68	47	56	-27	5	5
C(130)	69	62	50	-30	0	-10
C(131)	16	18	20	-5	-5	-1
C(132)	31	32	35	-10	-3	-6
C(133)	32	35	33	-9	-6	-1
C(134)	37	37	38	-13	-6	0
C(135)	35	35	36	-15	-5	1
C(136)	31	31	29	-10	-8	1
C(137)	37	40	39	-11	-6	1
C(138)	61	61	51	-15	-8	2
C(139)	60	79	69	-23	-7	-15
C(140)	85	78	87	-30	0	12
C(141)	26	21	25	-10	-4	2
C(142)	23	27	22	-8	-5	3
C(143)	31	35	33	-12	-8	0
C(144)	36	37	36	-13	-1	1
C(145)	57	53	55	-22	-6	-1
C(146)	45	45	46	-18	-5	-2
C(147)	51	50	51	-19	-5	-2
C(148)	74	60	65	-33	-13	3
C(149)	68	58	52	-26	1	-9
C(150)	68	76	74	-29	-24	8
C(151)	21	16	21	-5	-8	-1
C(152)	26	27	29	-9	-8	-2
C(153)	40	39	41	-10	-10	-2
C(154)	43	43	44	-15	-6	2
C(155)	41	41	37	-14	-10	-1

C(156)	36	31	34	-11	-7	-4
C(157)	57	59	56	-22	-7	1
C(158)	75	71	69	-22	1	-1
C(159)	78	83	77	-34	-11	-1
C(160)	95	89	98	-41	-7	2
C(161)	20	19	23	-7	-2	0
C(162)	27	31	29	-9	-8	1
C(163)	39	42	36	-14	-10	1
C(164)	33	35	31	-9	-7	0
C(165)	42	41	42	-15	-9	1
C(166)	23	24	24	-7	-6	5
C(167)	38	37	39	-8	-10	2
C(168)	59	56	57	-22	-6	8
C(169)	47	60	57	-11	-25	-9
C(170)	45	49	41	-3	-23	2
C(171)	29	27	28	-13	-1	0
C(172)	27	25	24	-9	-4	2
C(173)	48	44	45	-17	-8	-1
C(174)	38	38	39	-14	-9	-2
C(175)	29	30	30	-9	-5	0
C(176)	35	35	33	-11	-9	-4
C(177)	47	50	45	-16	-8	-2
C(178)	71	76	68	-22	-2	5
C(179)	79	84	80	-28	-6	-8
C(180)	102	110	95	-44	-6	-2
C(181)	19	21	23	-9	-3	-1
C(182)	36	40	41	-15	-8	-2
C(183)	42	41	45	-15	-8	-1
C(184)	32	32	34	-12	-5	0
C(185)	34	35	33	-14	-9	2
C(186)	30	32	31	-12	-8	-1
C(187)	42	43	46	-15	-10	3
C(188)	44	53	55	-22	-1	6
C(189)	60	67	70	-26	-25	1
C(190)	47	58	69	-21	-15	4
C(191)	19	26	19	-7	-3	-2
C(192)	29	33	33	-10	-9	-2
C(193)	27	30	30	-9	-10	0

C(194)	45	48	47	-16	-12	0
C(195)	40	40	39	-13	-9	0
C(196)	23	27	27	-11	-2	1
C(197)	57	60	59	-22	-14	0
C(198)	80	86	74	-23	-22	-3
C(199)	88	92	100	-25	-27	-14
C(200)	91	100	102	-32	-19	3
C(201)	19	14	16	-7	-5	0
C(202)	28	30	27	-11	-4	-1
C(203)	27	26	22	-11	-8	0
C(204)	27	27	25	-10	-11	-5
C(205)	24	30	29	-8	-8	0
C(206)	30	27	29	-8	-8	3
C(207)	31	32	31	-9	-7	0
C(208)	43	34	35	-10	-16	-3
C(209)	39	44	42	-11	-16	9
C(210)	49	44	37	-10	-15	-5
C(211)	28	29	29	-8	-6	-1
C(212)	31	30	32	-14	-4	2
C(213)	33	31	32	-12	-5	-1
C(214)	45	45	44	-15	-7	-1
C(215)	51	49	47	-15	-11	2
C(216)	32	33	34	-16	-5	0
C(217)	63	64	61	-24	-11	-2
C(218)	88	81	76	-32	-3	7
C(219)	99	101	86	-31	-14	1
C(220)	93	85	80	-33	-4	-10
C(221)	29	28	23	-10	-3	-3
C(222)	58	57	55	-18	-12	-2
C(223)	62	57	58	-23	-9	-2
C(224)	91	90	89	-33	-21	-2
C(225)	53	53	51	-21	-8	-4
C(226)	41	39	37	-16	-4	-1
C(227)	103	102	102	-39	-21	-4
C(228)	125	126	121	-34	-30	-11
C(229)	132	121	124	-52	-12	-7
C(230)	21	23	23	-9	-5	-1
C(231)	34	32	31	-11	-6	-2

C(232)	43	44	44	-15	-10	-1
C(233)	38	32	32	-16	-4	1
C(234)	43	44	41	-16	-9	-4
C(235)	27	28	27	-12	-4	-1
C(236)	59	59	53	-22	-11	2
C(237)	80	79	77	-37	-6	-2
C(238)	102	102	99	-49	-20	-3
C(239)	106	105	102	-38	-20	8
C(240)	22	24	23	-7	-5	0
C(241)	31	32	35	-13	-4	0
C(242)	30	30	28	-11	-7	-2
C(243)	36	39	38	-15	-8	-3
C(244)	39	43	39	-14	-11	-1
C(245)	38	39	37	-16	-8	-4
C(246)	52	53	53	-17	-14	-4
C(247)	126	123	128	-43	-37	-7
C(248)	103	105	101	-33	-31	-18
C(249)	135	129	136	-52	-29	-5
C(250)	27	31	29	-10	-3	2
C(251)	13	18	15	-2	-1	-2
C(252)	25	25	25	-9	-3	1
C(253)	26	28	26	-8	-4	3
C(254)	30	28	30	-10	-4	1
C(255)	32	32	31	-12	-6	-2
C(256)	44	47	45	-15	-6	1
C(257)	81	67	70	-31	-11	-9
C(258)	152	148	145	-54	-25	6
C(259)	108	122	111	-42	-27	-1
C(260)	112	116	115	-38	-17	-3

Table 5. Hydrogen coordinates (x 10^4) and isotropic displacement parameters ($Å^2$ x 10^3) for $Au_{42}(TBBT)_{26}$.

	x	y	z	U(eq)
H(2)	601	7024	3033	30
H(3)	129	7049	3739	52
H(5)	1128	5799	4373	40
H(6)	1648	5814	3668	36
H(8A)	353	5666	5067	78
H(8B)	344	6187	5231	78
H(8C)	870	6084	4884	78
H(9A)	-519	6466	4396	113
H(9B)	-585	6228	4930	113
H(9C)	-321	5830	4655	113
H(10A)	565	7151	4598	176
H(10B)	-59	7055	4877	176
H(10C)	63	7318	4340	176
H(12)	738	4752	1100	48
H(13)	338	4404	667	65
H(15)	1558	3208	708	48
H(16)	1950	3548	1149	34
H(18A)	112	2835	954	147
H(18B)	-212	3087	550	147
H(18C)	-174	3452	846	147
H(19A)	147	4184	124	138
H(19B)	226	3786	-164	138
H(19C)	727	4166	-203	138
H(20A)	1226	3024	110	250
H(20B)	628	2776	183	250
H(20C)	907	2653	604	250
H(22)	446	5711	1420	66
H(23)	-99	6464	993	82
H(25)	-962	6650	2143	70
H(26)	-374	5953	2573	75
H(28A)	-451	7751	774	261
H(28B)	-896	7586	559	261
H(28C)	-356	7195	646	261

H(29A)	-1292	6466	1081	247
H(29B)	-1556	7089	880	247
H(29C)	-1733	6713	1404	247
H(30A)	-1437	7295	1756	268
H(30B)	-948	7735	1466	268
H(30C)	-1485	7749	1269	268
H(32)	2219	3993	5182	31
H(33)	2844	4079	5599	38
H(35)	3407	5582	4600	43
H(36)	2735	5529	4210	27
H(38A)	3537	5776	5214	56
H(38B)	3860	5498	5617	56
H(38C)	3205	5427	5709	56
H(39A)	3274	4354	6048	61
H(39B)	3917	4450	5999	61
H(39C)	3732	4023	5803	61
H(40A)	4304	4537	5055	65
H(40B)	4500	4977	5234	65
H(40C)	4233	5208	4798	65
H(42)	2293	1772	5285	36
H(43)	2528	1394	6005	36
H(45)	1889	2789	6254	54
H(46)	1598	3137	5577	44
H(48A)	2409	2558	6771	117
H(48B)	2457	2002	7205	117
H(48C)	1888	2151	7032	117
H(49A)	3247	1529	6401	139
H(49B)	3244	1711	6817	139
H(49C)	3245	2189	6330	139
H(50A)	1874	1209	6907	143
H(50B)	2324	1092	7212	143
H(50C)	2472	960	6760	143
H(52)	5497	3957	2398	40
H(53)	6241	4493	2342	39
H(55)	5931	4019	3688	42
H(56)	5184	3486	3757	41
H(58A)	7157	4707	2370	79
H(58B)	7228	5248	2491	79

H(58C)	6686	5191	2324	79
H(59A)	6267	5414	3003	78
H(59B)	6794	5279	3232	78
H(59C)	6234	4936	3494	78
H(60A)	6931	4065	3555	90
H(60B)	7425	4456	3200	90
H(60C)	7203	3949	3107	90
H(62)	5568	4356	1559	33
H(63)	6439	4723	1460	34
H(65)	5826	6331	978	31
H(66)	4965	5964	1099	35
H(68A)	7044	5818	340	75
H(68B)	7314	6346	371	75
H(68C)	6668	6355	373	75
H(69A)	7200	5076	1443	70
H(69B)	7660	5496	1068	70
H(69C)	7316	5105	936	70
H(70A)	6624	6532	1165	87
H(70B)	7274	6401	1112	87
H(70C)	6855	6032	1558	87
H(72)	5674	3366	1448	43
H(73)	6557	3244	1553	33
H(75)	6055	2155	2843	44
H(76)	5152	2215	2721	29
H(78A)	6948	2213	2981	132
H(78B)	7532	2129	2684	132
H(78C)	6996	1820	2691	132
H(79A)	7276	2176	1913	140
H(79B)	7777	2479	1968	140
H(79C)	7392	2838	1625	140
H(80A)	6942	3560	2098	157
H(80B)	7553	3326	2153	157
H(80C)	7078	3259	2588	157
H(82)	2886	5881	566	37
H(83)	2236	6286	107	44
H(85)	2085	4747	7	33
H(86)	2783	4334	455	34
H(88A)	1686	5162	-520	78

H(88B)	1173	5582	-640	78
H(88C)	1139	5120	-143	78
H(89A)	1046	5741	331	102
H(89B)	828	6156	-106	102
H(89C)	1277	6375	73	102
H(90A)	2035	6619	-628	110
H(90B)	1660	6479	-911	110
H(90C)	2245	6164	-862	110
H(92)	2988	3270	685	38
H(93)	2546	2812	341	43
H(95)	2937	1247	1177	39
H(96)	3355	1697	1545	36
H(98A)	1980	1234	1052	130
H(98B)	1922	1034	657	130
H(98C)	1627	1611	688	130
H(99A)	3283	1418	260	168
H(99B)	2874	1318	-16	168
H(99C)	2853	905	494	168
H(10D)	1951	2320	85	137
H(10E)	2232	1913	-178	137
H(10F)	2586	2403	-170	137
H(102)	423	3412	1941	47
H(103)	-314	3945	1579	54
H(105)	-880	4503	2596	38
H(106)	-212	3990	2976	43
H(10G)	-600	5299	1284	72
H(10H)	-1128	5226	1108	72
H(10I)	-623	4781	1124	72
H(10J)	-1608	4879	2294	70
H(10K)	-1713	5283	1809	70
H(10L)	-1162	5348	1960	70
H(11A)	-1347	4002	1599	82
H(11B)	-1849	4425	1669	82
H(11C)	-1653	3952	2095	82
H(112)	1773	1124	2199	90
H(113)	1169	507	2174	96
H(115)	297	1759	1444	59
H(116)	865	2475	1500	60

H(11D)	-206	1281	1424	190
H(11E)	-418	652	1549	190
H(11F)	90	906	1138	190
H(11G)	340	60	2404	187
H(11H)	-115	-104	2197	187
H(11I)	-239	396	2391	187
H(12A)	963	448	1322	190
H(12B)	458	19	1486	190
H(12C)	856	-25	1819	190
H(122)	4044	1656	2328	27
H(123)	4728	1491	1767	36
H(125)	3773	255	1804	44
H(126)	3068	447	2348	40
H(12D)	5247	1461	1086	102
H(12E)	5596	934	1002	102
H(12F)	5452	961	1493	102
H(12G)	5024	-67	1748	87
H(12H)	5171	2	1231	87
H(12I)	4553	-145	1515	87
H(13A)	4178	655	973	88
H(13B)	4750	927	655	88
H(13C)	4318	1298	879	88
H(132)	91	852	3530	40
H(133)	-686	722	4139	41
H(135)	274	1040	4873	42
H(136)	1057	1104	4293	37
H(13D)	-474	183	5434	90
H(13E)	-984	497	5647	90
H(13F)	-396	800	5437	90
H(13G)	-1456	707	4688	106
H(13H)	-1520	345	5214	106
H(13I)	-1085	146	4858	106
H(14A)	-758	1627	4968	131
H(14B)	-1382	1414	5102	131
H(14C)	-1069	1643	4587	131
H(142)	3715	150	3329	29
H(143)	4087	-514	2952	40
H(145)	2733	-1484	3571	66

H(146)	2402	-919	3998	55
H(14D)	3057	-2069	3306	96
H(14E)	3387	-2138	2851	96
H(14F)	2946	-1631	2832	96
H(14G)	3573	-816	2361	89
H(14H)	3944	-1329	2273	89
H(14I)	4204	-887	2413	89
H(15A)	4442	-1569	3164	107
H(15B)	4333	-2079	3019	107
H(15C)	4018	-2062	3496	107
H(152)	5433	941	3888	33
H(153)	6164	740	3388	49
H(155)	5096	795	2578	47
H(156)	4383	1095	3061	40
H(15D)	5933	1201	2024	113
H(15E)	6571	1032	1907	113
H(15F)	6387	1443	2183	113
H(15G)	6869	824	2732	118
H(15H)	7013	338	2523	118
H(15I)	6696	178	3035	118
H(16A)	5937	-260	2824	142
H(16B)	6342	-78	2341	142
H(16C)	5712	139	2387	142
H(162)	4786	2112	4365	36
H(163)	5390	1524	4828	47
H(165)	4121	1195	5944	50
H(166)	3538	1786	5478	29
H(16D)	5174	259	5483	88
H(16E)	5690	151	5722	88
H(16F)	5727	619	5227	88
H(16G)	5923	1481	5381	82
H(16H)	5991	1075	5876	82
H(16I)	5566	1603	5807	82
H(17A)	4882	895	6360	70
H(17B)	5231	327	6374	70
H(17C)	4638	422	6242	70
H(172)	2261	411	5343	31
H(173)	1598	301	6010	55

H(175)	681	1590	5320	37
H(176)	1348	1756	4653	41
H(17D)	367	1733	6040	113
H(17E)	-59	1288	6430	113
H(17F)	-3	1352	5919	113
H(17G)	141	348	6119	125
H(17H)	30	322	6625	125
H(17I)	579	38	6428	125
H(18D)	1132	514	6630	154
H(18E)	689	925	6802	154
H(18F)	1216	1189	6413	154
H(182)	106	5706	3797	47
H(183)	-791	5994	3691	52
H(185)	-942	4568	3425	40
H(186)	-76	4267	3587	36
H(18G)	-1879	4643	3923	79
H(18H)	-2366	5110	3857	79
H(18I)	-1961	5074	4183	79
H(18J)	-1502	5756	2800	95
H(18K)	-2099	5491	3060	95
H(18L)	-1573	5079	3031	95
H(19D)	-1771	6077	3816	88
H(19E)	-2119	6192	3435	88
H(19F)	-1479	6355	3297	88
H(192)	261	2153	3737	38
H(193)	-556	2120	3504	35
H(195)	-1254	3305	4005	48
H(196)	-448	3317	4267	31
H(19G)	-1345	3223	2892	122
H(19H)	-1702	2689	2968	122
H(19I)	-1059	2607	2982	122
H(19J)	-1504	1844	3631	141
H(19K)	-2122	2095	3673	141
H(19L)	-1810	1943	4080	141
H(20D)	-2093	3025	3983	149
H(20E)	-2306	3110	3538	149
H(20F)	-1826	3505	3516	149
H(202)	3388	3537	4842	34

H(203)	3890	3044	5403	29
H(205)	5286	3859	4566	33
H(206)	4769	4351	4012	35
H(20G)	5403	3841	5353	55
H(20H)	5325	3377	5853	55
H(20I)	4808	3756	5683	55
H(20J)	5442	2625	5060	64
H(20K)	5757	2647	5427	64
H(20L)	5768	3187	4970	64
H(21A)	4336	2769	5966	65
H(21B)	4906	2447	6066	65
H(21C)	4594	2322	5736	65
H(212)	2726	6735	876	37
H(213)	3210	7226	158	38
H(215)	1742	7733	-240	60
H(216)	1256	7184	496	39
H(21D)	3519	7288	-622	126
H(21E)	3410	7651	-1114	126
H(21F)	3003	7146	-778	126
H(21G)	2257	7876	-962	146
H(21H)	2703	8365	-1251	146
H(21I)	2252	8430	-839	146
H(22A)	2990	8594	-516	130
H(22B)	3448	8526	-923	130
H(22C)	3491	8150	-415	130
H(222)	2780	7784	1022	69
H(223)	3438	8403	451	71
H(225)	3872	8961	1346	62
H(226)	3169	8370	1913	47
H(22D)	3529	9684	32	188
H(22E)	4156	9755	-241	188
H(22F)	3823	9193	-137	188
H(22G)	3910	9842	688	189
H(22H)	4389	10012	245	189
H(22I)	4519	9579	714	189
H(231)	3906	6673	3719	39
H(232)	3487	6850	4349	53
H(234)	1955	6794	4067	51

H(235)	2398	6665	3406	32
H(23A)	1784	6328	4953	117
H(23B)	1923	6468	5353	117
H(23C)	2352	6105	5118	117
H(23D)	3143	6987	4842	146
H(23E)	2651	6983	5253	146
H(23F)	2802	7560	4826	146
H(23G)	2079	7803	4365	158
H(23H)	1764	7541	4879	158
H(23I)	1632	7314	4520	158
H(241)	4758	5699	3252	40
H(242)	5260	6421	3276	35
H(244)	6140	6664	2014	48
H(245)	5687	5921	2008	44
H(24A)	6317	6984	3278	187
H(24B)	6822	6867	2922	187
H(24C)	6353	6398	3196	187
H(24D)	6814	7132	2176	152
H(24E)	6652	7731	2231	152
H(24F)	6310	7501	1975	152
H(24G)	5569	7750	2513	198
H(24H)	5928	7774	2853	198
H(24I)	5419	7348	3032	198
H(251)	3615	5929	1033	20
H(252)	4138	6082	320	30
H(254)	4884	7420	325	36
H(255)	4343	7311	1034	38
H(25A)	4843	6003	-113	107
H(25B)	5210	6328	-601	107
H(25C)	4556	6440	-503	107
H(25D)	4546	7422	-632	225
H(25E)	5193	7426	-843	225
H(25F)	4968	7700	-469	225
H(25G)	5727	7065	-161	170
H(25H)	5785	6621	-409	170
H(25I)	5570	6411	123	170
H(26A)	4826	8803	582	175
H(26B)	4866	9114	53	175

H(26C)	4560	8516	318	175
